# Lysyl oxidase inhibition disrupts mitochondrial homeostasis to create vulnerability to ferroptosis in TNBC

**DOI:** 10.1016/j.xcrm.2026.103015

**Published:** 2026-08-31

**Authors:** Ozge Saatci, Burge Ulukan, Metin Cetin, Hellen Kuasne, Constanza Martinez, Elif Percin, Natalia Oleinik, Matthew T. Savoca, Ali Nehme, Aldo Hernández-Corchado, Evelyn Zavacky, Kukkamudi Sreenivas, Abdol H. Rezaeian, Jennifer R. Bethard, Jean-Sebastien Anoma, Ozlem Sener Sahin, Adriana Aguilar-Mahecha, Marguerite Buchanan, Mertkaya Aras, Genevieve DeBlois, Mark Basik, Elizabeth G. Hill, Lauren E. Ball, Chintada Nageswara Rao, Campbell McInnes, Yasser Riazalhosseini, Hamed S. Najafabadi, John J. Lemasters, Besim Ogretmen, Morag Park, Ozgur Sahin

**Affiliations:** 1 Department of Biochemistry and Molecular Biology, Hollings Cancer Center, Medical University of South Carolina, Charleston, SC 29425, USA; 2 Rosalind and Morris Goodman Cancer Institute, McGill University, Montréal, QC H3A 1A3, Canada; 3 Department of Radiation Oncology, McGill University Health Centre, Montréal, QC H4A 3J1, Canada; 4 Department of Drug Discovery and Biomedical Sciences, Medical University of South Carolina, Charleston, SC 29425, USA; 5 Department of Human Genetics, McGill University, Montréal, QC H3A 0G1, Canada; 6 Department of Drug Discovery and Biomedical Sciences, University of South Carolina, Columbia, SC 29208, USA; 7 Department of Pharmacology & Immunology, Proteomics Center, Medical University of South Carolina, Charleston, SC 29425, USA; 8 Department of Public Health Sciences, Medical University of South Carolina, Charleston, SC 29425, USA; 9 Lady Davis Institute, Jewish General Hospital, McGill University, Montréal, QC H3T 1E2, Canada; 10 Institute for Research in Immunology and Cancer (IRIC), Université de Montréal, Montréal, QC H3T 1J4, Canada; 11 Faculty of Pharmacy, Université de Montréal, Montréal, QC H3T 1J4, Canada; 12 Victor Phillip Dahdaleh Institute of Genomic Medicine at McGill University, Montréal, QC H3A 0G1, Canada; 13 Department of Biochemistry, McGill University, Montréal, QC H3A 0G1, Canada; 14 Department of Oncology, McGill University, Montréal, QC H3A 0G1, Canada

**Keywords:** lysyl oxidase, LOX, glucose metabolism, mitophagy, mitochondria-ER contacts, MERCS, DHODH, ferroptosis, TNBC

## Abstract

High metabolic heterogeneity and plasticity of triple-negative breast cancer (TNBC) contribute to therapy resistance, necessitating identification of therapeutic vulnerabilities. Here, we identify non-canonical functions of the extracellular matrix (ECM) remodeler, lysyl oxidase (LOX), in regulating glucose metabolism and mitochondrial homeostasis and show that inhibiting LOX generates targetable vulnerability to ferroptosis. Mechanistically, LOX interacts with PARKIN and its upstream kinase PINK1, which we identified as a substrate of LOX. LOX-mediated PINK1 oxidation suppresses PARKIN phosphorylation, stabilizing hypoxia-inducible factor 1-alpha (HIF-1α) and increasing glycolysis. Concomitantly, LOX inhibits PARKIN-mediated mitophagy and maintains mitochondria-ER contacts through VDAC1 stabilization, while the LOX-HSP90 complex promotes mitochondrial Ca^2+^ transport and ATP production. Inhibiting LOX suppresses glycolysis, disrupts mitochondrial dynamics, reduces OXPHOS and GPX4/FSP1, and induces compensatory DHODH activity. Our “one-two punch” approach combining LOX inhibition with clinical DHODH inhibitor suppresses tumor growth *in vivo* in chemo-free setting. Notably, LOX protein correlates with HIF-1α/GLUT1/GPX4 in TNBC patient tumors, supporting its clinical relevance.

## Highlights

• LOX oxidizes PINK1 and suppresses PARKIN, promoting HIF-1α-mediated glycolysis
• LOX inhibits PARKIN-mediated mitophagy, stabilizes VDAC1, and promotes MERCs
• LOX-HSP90-IP_3_R sustains MERCs, mitochondrial Ca^2+^ transfer, and ATP
• Inhibiting LOX generates a targetable vulnerability to DHODH inhibition in TNBC

## Graphical abstract

**Figure:**
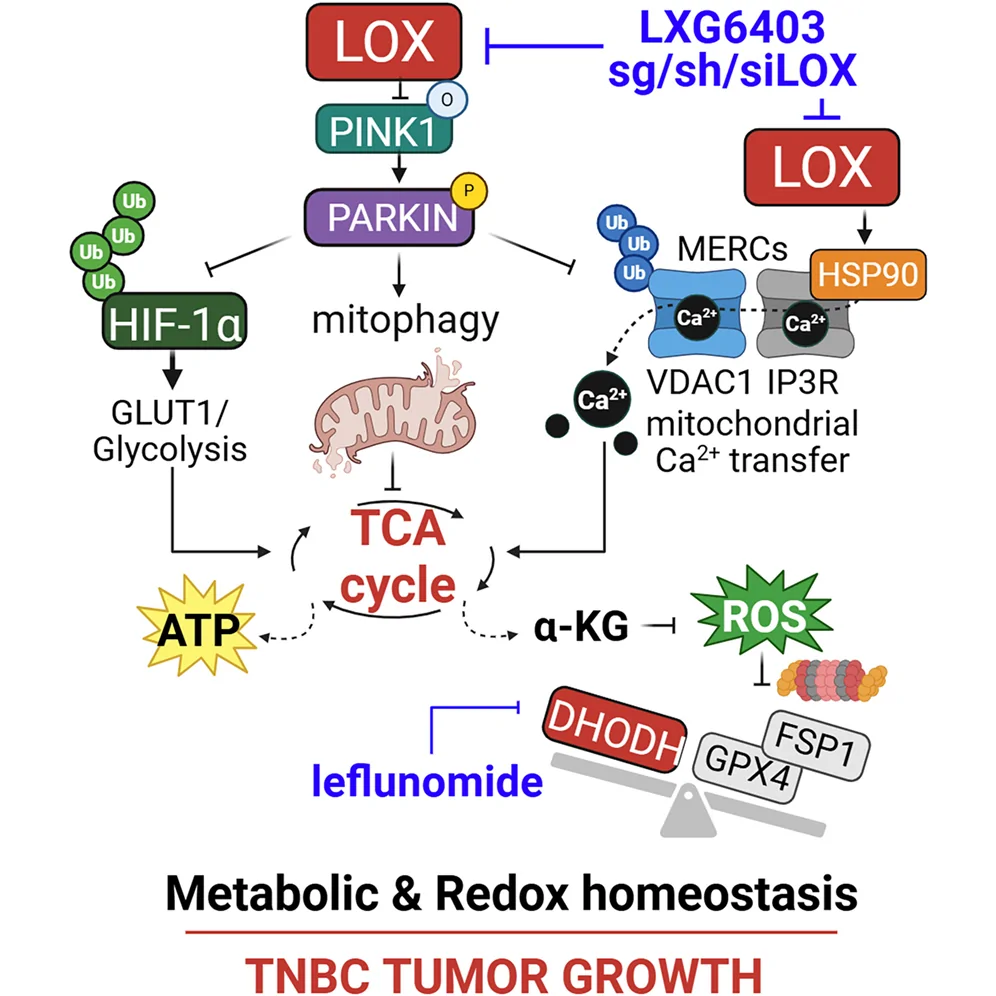

## Teaser

Saatci-Ulukan et al. show that lysyl oxidase (LOX) has non-canonical intracellular functions in promoting glucose metabolism, inhibiting mitophagy, and sustaining redox homeostasis and that inhibiting LOX creates a targetable vulnerability to DHODH-inhibition-mediated ferroptosis in chemo-free setting in triple-negative breast cancer, the deadliest subtype.

## Introduction

Ferroptosis is an iron-mediated cell death^1^ characterized by aberrant cellular redox control and distinct from other regulated cell death mechanisms, as it is constantly suppressed without an activating signal.^2^ Ferroptosis induction has been increasingly recognized as a highly attractive therapeutic strategy that might provide therapeutic opportunities in treating cancers that are refractory to conventional therapies^3^^,^^4^ due to its crosstalk with other hallmarks of cancer.^2^ Ferroptosis regulation is multifaceted and includes different redox defense mechanisms to survive under metabolic and oxidative stress. These include the glutathione peroxidase 4 (GPX4)-reduced glutathione (GSH) system,^5^ the ferroptosis suppressor protein 1 (FSP1)-ubiquinol (CoQH2) system,^6^ and the dihydroorotate dehydrogenase (DHODH)-CoQH2 system.^7^ Consequently, targeting those defense mechanisms in isolation has been tested pre-clinically with limited success due to metabolic/redox plasticity of the cancer cells^8^ and activation of compensatory defense mechanisms.^9^ Thus, despite substantial advances in understanding how cancer cells evade ferroptosis, its translational potential still remains speculative, and little is known how to exploit the metabolic/redox plasticity to generate vulnerability to ferroptosis-mediated cell death to treat highly aggressive cancers.^2^

Mitochondria are the centers for cellular energy generation, metabolic regulation, and signal transduction that are critical for tumorigenesis.^10^ Furthermore, mitochondria are integral part of adaptive responses that not only respond to cellular energy demands by hosting oxidative phosphorylation (OXPHOS) and tricarboxylic acid (TCA) cycle^11^ but also maintain redox homeostasis and intracellular Ca^2+^ levels through coupling with endoplasmic reticulum (ER).^12^ Given its central roles in cell survival and proliferation, disruption of mitochondrial biogenesis is detrimental.^13^ Mitochondrial fusion enhances mitochondrial integrity and is mediated by mitofusins 1 and 2 (MFN1/2)^14^ and OPA1,^15^ which coordinate outer and inner membrane fusion, respectively, to ensure efficient ATP production. Upon loss of mitochondrial integrity under stress, dysfunctional mitochondria are eliminated by a process called mitophagy.^16^^,^^17^ However, excessive activation of mitophagy due to persistent insults or oxidative stress may result in cell death. Furthermore, given that key mediators of the antioxidant defense are also localized to mitochondria, e.g., GPX4 and DHODH,^18^ there is an intricate interplay between mitochondrial dysfunction and ferroptosis.^19^ However, the underlying mechanisms of the crosstalk between mitochondrial biogenesis, mitophagy, and ferroptosis, and whether it can be therapeutically exploited to generate vulnerability to ferroptosis-mediated cell death, are poorly understood.

Lysyl oxidase (LOX) is a copper-dependent amine oxidase that is strongly induced under hypoxic conditions and plays a central role in extracellular matrix (ECM) remodeling.^20^^,^^21^ LOX promotes collagen crosslinking and matrix stiffening, which contributes to tumor progression, invasion, and the establishment of pre-metastatic niche.^22^^,^^23^ High LOX expression has been associated with poor prognosis,^24^^,^^25^ chemoresistance,^26^ and metastasis^27^ across multiple cancer types. Despite these well-established extracellular functions, whether LOX regulates energy metabolism and mitochondrial homeostasis in cancer cells remains poorly understood.

In this study, we find that the ECM remodeling enzyme LOX has non-canonical functions in promoting glucose metabolism, inhibiting mitophagy, and sustaining redox homeostasis. We show that genomic or pharmacologic inhibition of LOX, on one hand, triggers PINK1-mediated phosphorylation and activation of PARKIN, leading to ubiquitination and degradation of the glycolysis regulator hypoxia-inducible factor 1-alpha (HIF-1α) and the mitochondria-ER contact site (MERC)-associated protein VDAC1, thereby reducing glucose metabolism and disrupting MERCs. On the other hand, LOX inhibition blocks mitochondrial Ca^2+^ transfer and OXPHOS by disrupting the LOX-HSP90 complex. Furthermore, LOX inhibition disrupts redox homeostasis by downregulating the antioxidant defense elements GPX4 and FSP1 while triggering compensatory DHODH activity in a mitophagy-dependent manner. These lead to increased vulnerability to DHODH targeting upon LOX inhibition in multiple clinically relevant mouse models in chemo-free settings.

## Results

### LOX regulates glucose metabolism in TNBC

**Figure 1:**
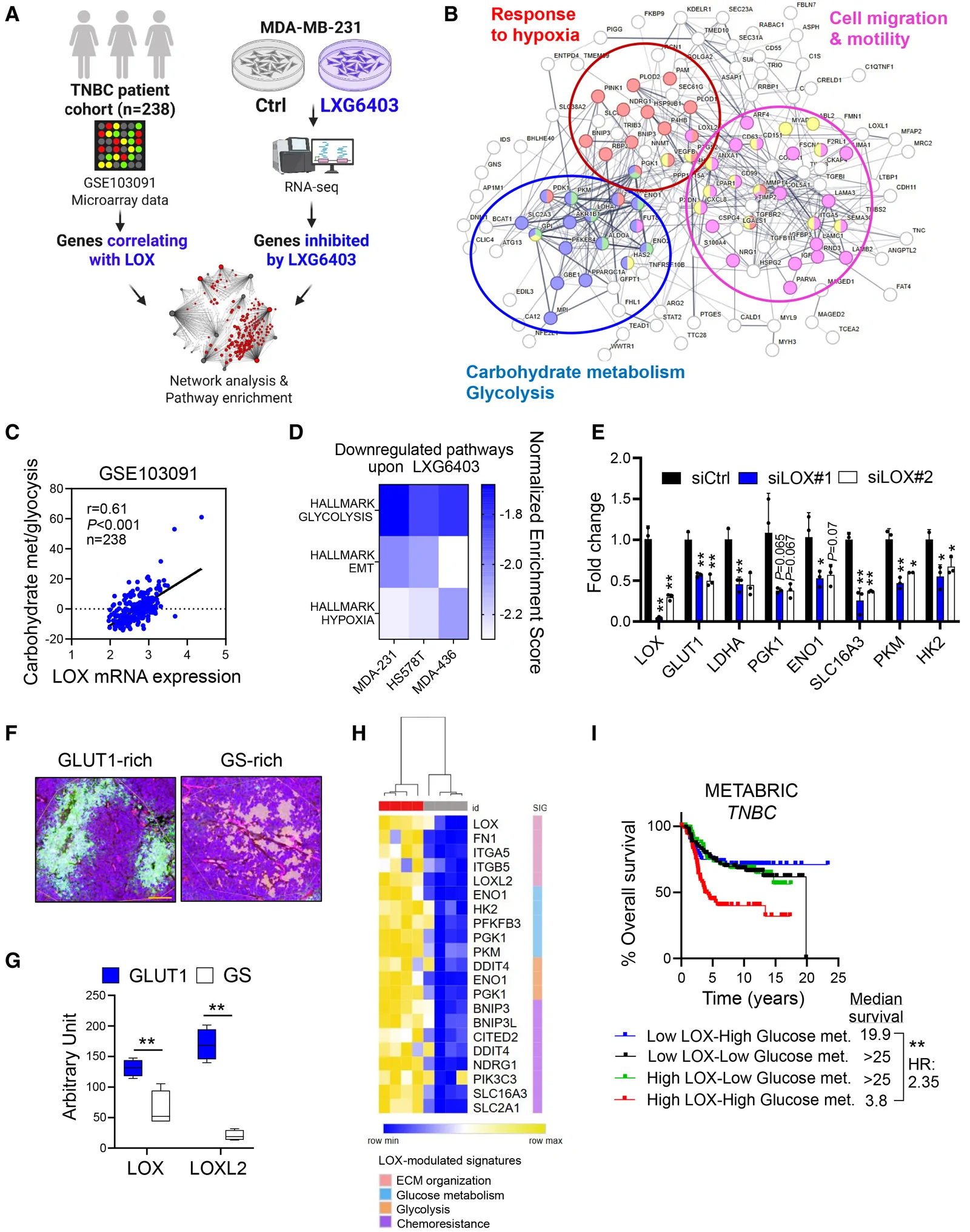
LOX regulates glucose metabolism in TNBC (A) Schematic of the analysis pipeline to determine clinically relevant LOX-regulated mRNAs. (B) STRING network of clinically relevant LOX-regulated genes. (C) Correlation of carbohydrate metabolism/glycolysis score with LOX mRNA in TNBC patient tumors (*n* = 238 patient samples). (D) Heatmap of pathways enriched in downregulated mRNAs upon LOX inhibition using LXG6403 in TNBC cell lines (*n* = 2 biological replicates). (E) RT-qPCR of glycolytic genes upon siLOX in BT549 cells (*n* = 3 biological replicates). (F and G) GeoMx spatial profiling of GLUT1 and GS-rich regions of TNBC PDX tumors (*n* = 4 regions). Scale bars: 100 μm. (H) Heatmap of LOX-regulated gene signatures in GLUT1-rich vs. GS-rich regions of TNBC PDX tumors. (I) Survival analysis in TNBC patients from METABRIC based on LOX expression and glucose metabolism score (*n* = 254 patients). Data are presented as mean values ± SD. *p* values were calculated with unpaired, two-tailed Student’s *t* test (E and G) and the log rank (Mantel-Cox) test for Kaplan-Meier analysis (I). ∗*p* < 0.05; ∗∗*p* < 0.01.

To identify clinically relevant networks regulated by LOX in TNBC, we integrated LOX-correlating genes in TNBC patients (GSE103091) with LOX-regulated genes from high LOX-expressing MDA-MB-231 TNBC cells treated with the LOX inhibitor LXG6403^28^ (Figures 1A, S1A, and S1B). Pathway enrichment and network analyses of LOX-regulated genes revealed enrichment of three major modules: hypoxia, cell migration/motility, and carbohydrate/glucose metabolism (Figures 1B, 1C, and S1C–S1E). Consistently, glycolysis, epithelial-mesenchymal transition (EMT), and hypoxia were among the most strongly enriched pathways within genes downregulated by LXG6403 in three different TNBC cell lines (Figures 1D, S1A, and S1B). Silencing LOX reduced glycolytic gene expression in high LOX-expressing BT549 cells (Figures 1E, S1A, and S1F), suggesting that LOX regulates glucose metabolism. Since TNBC is a heterogeneous disease, we analyzed LOX expression and related processes in different TNBC subtypes. LOX expression was higher in basal-like 2 (BL2) and mesenchymal (MES) subtypes of TNBC both in cell lines (Figure S1A) and in patient tumors (Figure S1H). Notably, glucose-metabolism-related processes, e.g., pyruvate metabolism and oxidative phosphorylation, are enriched among LOX-correlated genes in BL2 and MES TNBC tumors (Figures S1I and S1J).

GeoMx spatial profiling in TNBC patient-derived xenograft (PDX) tumors revealed that LOX and its most oncogenic relative, LOXL2, were significantly higher in glucose transporter type 1 (GLUT1)-rich regions compared to glutamine synthetase (GS)-rich regions (Figures 1F and 1G). Pathway enrichment analysis showed enrichment of hypoxia, glycolysis, OXPHOS, and EMT processes in high GLUT1 regions (Figure S1G). Notably, genes related to ECM, the canonical LOX downstream,^26^^,^^29^ glucose metabolism, and glycolysis that are downregulated by LXG6403 (Figure 1A) were enriched in GLUT1-rich regions (Figure 1H). Furthermore, TNBC patient tumors expressing high levels of LOX and glucose-metabolism signature exhibit worse survival compared to all other groups (Figure 1I). Collectively, robust integration of bulk RNA sequencing (RNA-seq), spatial profiling, and network analysis revealed a functional and clinically relevant link between LOX and glucose metabolism in TNBC.

### LOX inhibition limits glucose uptake and reduces glycolysis and OXPHOS

**Figure 2:**
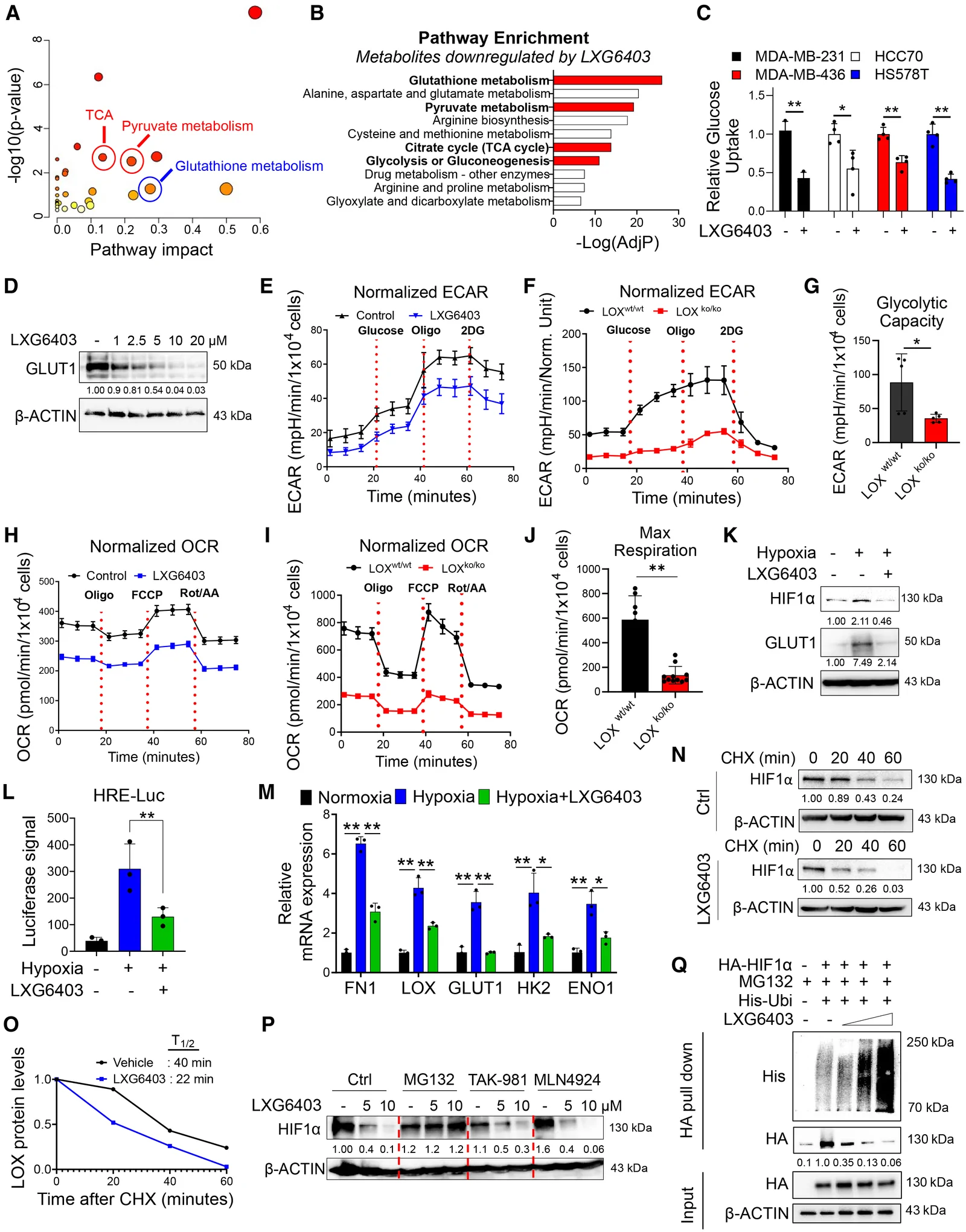
LOX inhibition limits glucose uptake and reduces glycolysis and OXPHOS (A and B) Pathway impact (A) and pathway enrichment (B) analyses of metabolites downregulated upon LXG6403 (15 μM) for 24 h in MDA-MB-231 (*n* = 3 biological replicates). (C) Glucose uptake levels upon LXG6403 (*n* = 3–4 technical replicates). (D) Western blot (WB) of GLUT1 in MDA-MB-231 cells treated with increasing doses of LXG6403. β-ACTIN was used as the loading control here and in all figures. The relative band intensities normalized to loading control based on densitometry analysis are provided below the respective blots here and in all WB subfigures. (E–G) Seahorse analyses in MDA-MB-231 cells upon LXG6403 (15 μM) for 24 h (E) (*n* = 5–6 technical replicates) and analyses in MEF cells obtained from LOX knockout mice (LOX^ko/ko^) showing glycolytic capacity (ECAR) (F and G) (*n* = 5–6 technical replicates). (H–J) Seahorse analyses in MDA-MB-231 cells upon LXG6403 (15 μM) for 24 h (H) (*n* = 12–15 technical replicates) and in LOX^ko/ko^ MEF cells showing maximal respiration (OCR) (I and J) (*n* = 8–17 technical replicates). (K and L) WB of HIF-1α and GLUT1 (K) and HRE reporter assay (L) in LXG6403-treated cells under hypoxia (*n* = 3 technical replicates). (M) RT-qPCR of HIF-1α targets upon LXG6403 under hypoxia (*n* = 3 technical replicates). (N and O) Cycloheximide chase assay of HIF-1α upon LOX inhibition (N) and its quantification (O). (P) WB of HIF-1α in BT549 cells upon LXG6403 and MG132, or TAK-981, or MLN4924 for 12 h under hypoxia. (Q) HIF-1α ubiquitination upon treatment with LXG6403 (5, 10, and 20 μM) for 18 h. Data are presented as mean values ± SD. *p* values were calculated with unpaired, two-tailed Student’s *t* test (C, G, J, L, and M), and one-way ANOVA with Dunnett’s post-hoc test (E, F, H, and I) ∗*p* < 0.05; ∗∗*p* < 0.01.

To test if LOX functionally regulates glucose utilization and bioenergetics in TNBC, we performed bulk metabolomics that revealed enrichment of pyruvate metabolism, glycolysis, TCA cycle, and glutathione metabolism among metabolites downregulated by LXG6403 (Figures 2A and 2B). Inhibiting LOX using either LXG6403 or single guide RNAs (sgRNAs) significantly reduced glucose uptake as well as lactate secretion (Figures 2C and S2A–S2C). LXG6403 reduced GLUT1 protein levels in a dose-dependent manner in TNBC cells (Figure 2D), similar to the mRNA downregulation of GLUT1 with siLOX (Figure 1E) and shLOX (Figure S2D). Seahorse analysis showed reduced glycolytic capacity (ECAR) upon LXG6403 in MDA-MB-231 cells (Figures 2E and S2E), and this effect was recapitulated in LOX-knockout mouse embryonic fibroblasts (MEFs) (Figures 2F, 2G, S2G, and S2H) obtained from LOX^ko/ko^ mice.^30^ Similarly, mitochondrial respiration (OCR) decreased upon LOX inhibition in MDA-MB-231 cells (Figures 2H and S2F) and in LOX-deficient MEFs (Figures 2I and 2J). Together, these data indicate that LOX inhibition diminishes glucose uptake and represses both glycolysis and OXPHOS in TNBC.

### LOX prevents HIF-1α protein degradation through inhibiting PINK1-PARKIN-dependent HIF-1α ubiquitination

To determine upstream regulators of LOX-mediated glycolytic gene transcription, we performed upstream analysis among genes downregulated by LXG6403. This identified HIF-1α (Figure S2I), the major hypoxia-induced transcription factor known to transcribe glycolysis genes.^31^ LOX inhibition under hypoxia using LXG6403 or siRNAs reduced HIF-1α protein level (Figures 2K and S2J). These ultimately reduced HIF-1α transcriptional activity (Figure 2L) and the mRNA and protein expression of canonical HIF-1α targets involved in glycolysis, including GLUT1 (Figures 2K, 2M, and S2J). LXG6403 reduced HIF-1α protein stability (Figures 2N and 2O), which was rescued upon proteasome inhibition with MG132 (Figure 2P), indicating proteasome-mediated HIF-1α degradation. Of note, inhibiting SUMOylation (TAK-981) or NEDDylation (MLN4924) did not rescue LOX-inhibition-mediated HIF-1α downregulation (Figure 2P). Furthermore, LXG6403 increased HIF-1α polyubiquitination in a dose-dependent manner (Figure 2Q).

**Figure 3:**
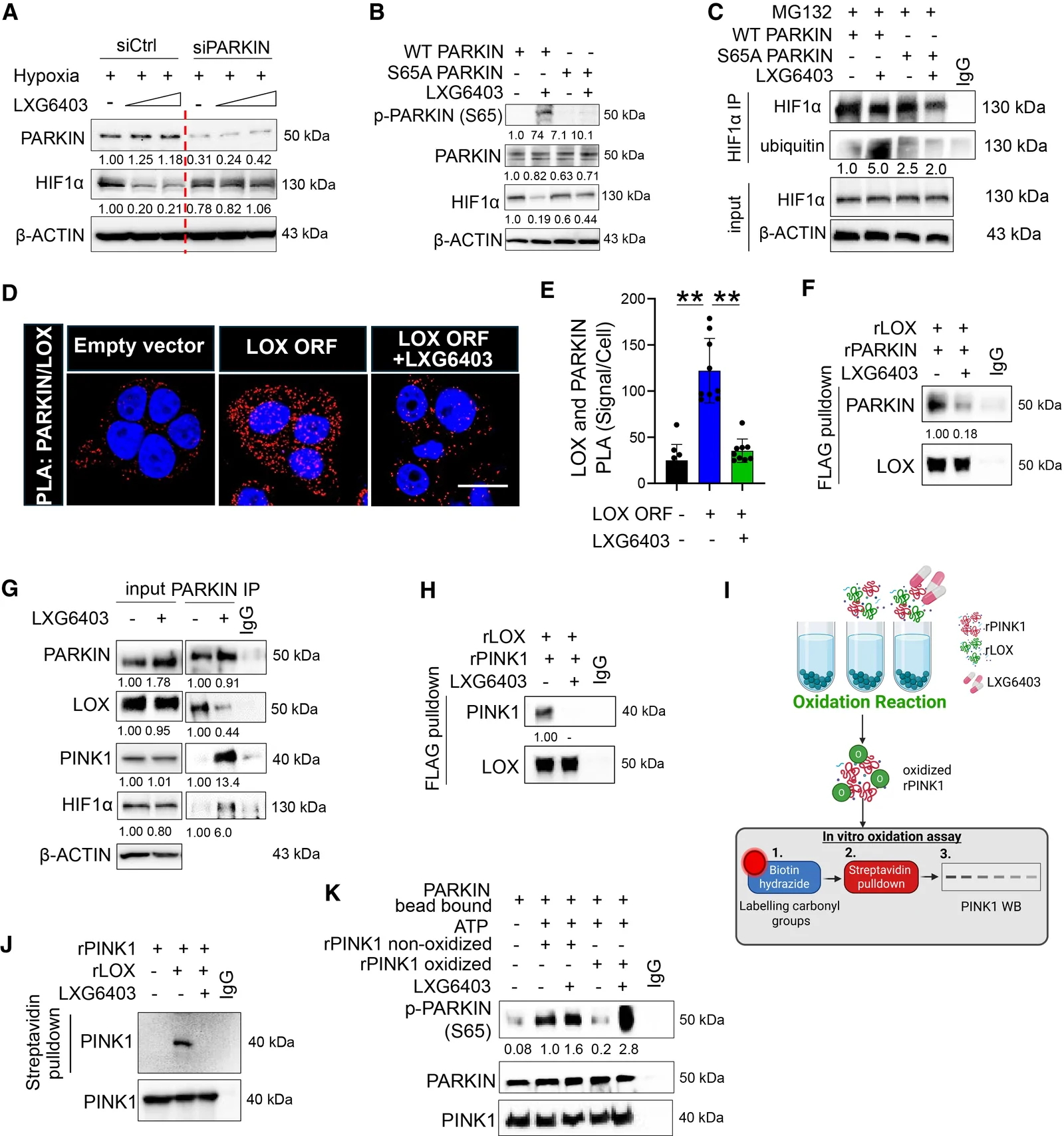
LOX prevents HIF-1α protein degradation by inhibiting PINK1 kinase activity and PARKIN-dependent ubiquitination (A) WB of PARKIN and HIF-1α upon siPARKIN and LXG6403 in MDA-MB-231 cells under hypoxia. (B) WB of PARKIN, p-PARKIN (S65), and HIF-1α in cells expressing WT or S65A PARKIN and treated with LXG6403. (C) HIF-1α ubiquitination in cells expressing WT or S65A PARKIN and treated with LXG6403 (30 μM) and MG132 (10 μM) for 6 h. (D) PLA of LOX and PARKIN in MDA-MB-468 cells overexpressing LOX and treated with LXG6403 (15 μM) for 12 h. Scale bars: 25 μm. (E) Quantification of PLA in (D) (*n* = 9 images). (F) Recombinant protein pull-down of LOX and PARKIN upon LXG6403. (G) Co-immunoprecipitation of PARKIN in MDA-MB-231 cells upon LXG6403. (H) Recombinant protein pull-down of LOX and PINK1 upon LXG6403. (I) Schematic overview of the cell-free *in vitro* oxidation assay. (J) Streptavidin pulldown of oxidized PINK1. (K) *In vitro* kinase assay with PARKIN and oxidized or non-oxidized recombinant PINK1 proteins upon LXG6403. Data are presented as mean values ±SD. *p* values were calculated with unpaired, two-tailed Student’s *t* test (E). ∗*p* < 0.05; *p* value < 0.01.

Among HIF-1α-ubiquitinating E3 ligases, VHL^32^ knockdown did not restore HIF-1α protein levels upon LOX inhibition (Figure S2K), whereas silencing PARKIN, another E3 ubiquitin ligase^33^ known to ubiquitinate HIF-1α,^34^ prevented LXG6403-mediated HIF-1α degradation (Figure 3A). Furthermore, LOX inhibition triggered PARKIN activation, as shown by induction of p-PARKIN (S65) (Figure 3B), while overexpressing the phospho-defective S65A mutant (Figure 3B) reverses HIF-1α ubiquitination by LXG6403 (Figure 3C) or genomic LOX knockdown (Figure S2L), thus rescuing HIF-1α levels (Figure 3B) and its transcriptional activity (Figure S2M). Importantly, we showed LOX interaction with PARKIN, which was abrogated upon LXG6403 (Figures 3D–3F). This further increases PARKIN association with PINK1, its upstream regulator and HIF-1α (Figure 3G), suggesting that LOX sequesters PARKIN away from PINK1, preventing its activation and HIF-1α ubiquitination. As an underlying mechanism, we showed that LOX interacts with PINK1, and this interaction was reduced by LXG6403 (Figure 3H). To test if PINK1 is a direct LOX substrate, which canonically oxidizes lysine residues,^35^ we performed *in vitro* biotin-hydrazide labeling followed by streptavidin pulldown (Figure 3I). We demonstrated that LOX directly oxidizes PINK1, which is completely abolished upon LXG6403 (Figure 3J). To assess the functionality of LOX-mediated PINK1 oxidation in PARKIN activation, we incubated recombinant PARKIN with PINK1 that had been pre-oxidized by LOX and observed reduced p-PARKIN (S65) (Figure 3K). On the contrary, incubation with PINK1, whose oxidation was inhibited by LXG6403, restored p-PARKIN (Figure 3K). As a control, we showed LOX-dependent oxidation of VGLL3, a known intracellular LOX substrate,^36^ under the same conditions (Figure S2N). These findings suggest that LOX oxidizes and inhibits PINK1 kinase activity, leading to PARKIN inactivation and thereby promoting HIF-1α protein stability.

### LOX inhibition impairs mitochondrial dynamics, leading to PARKIN-mediated mitophagy

**Figure 4:**
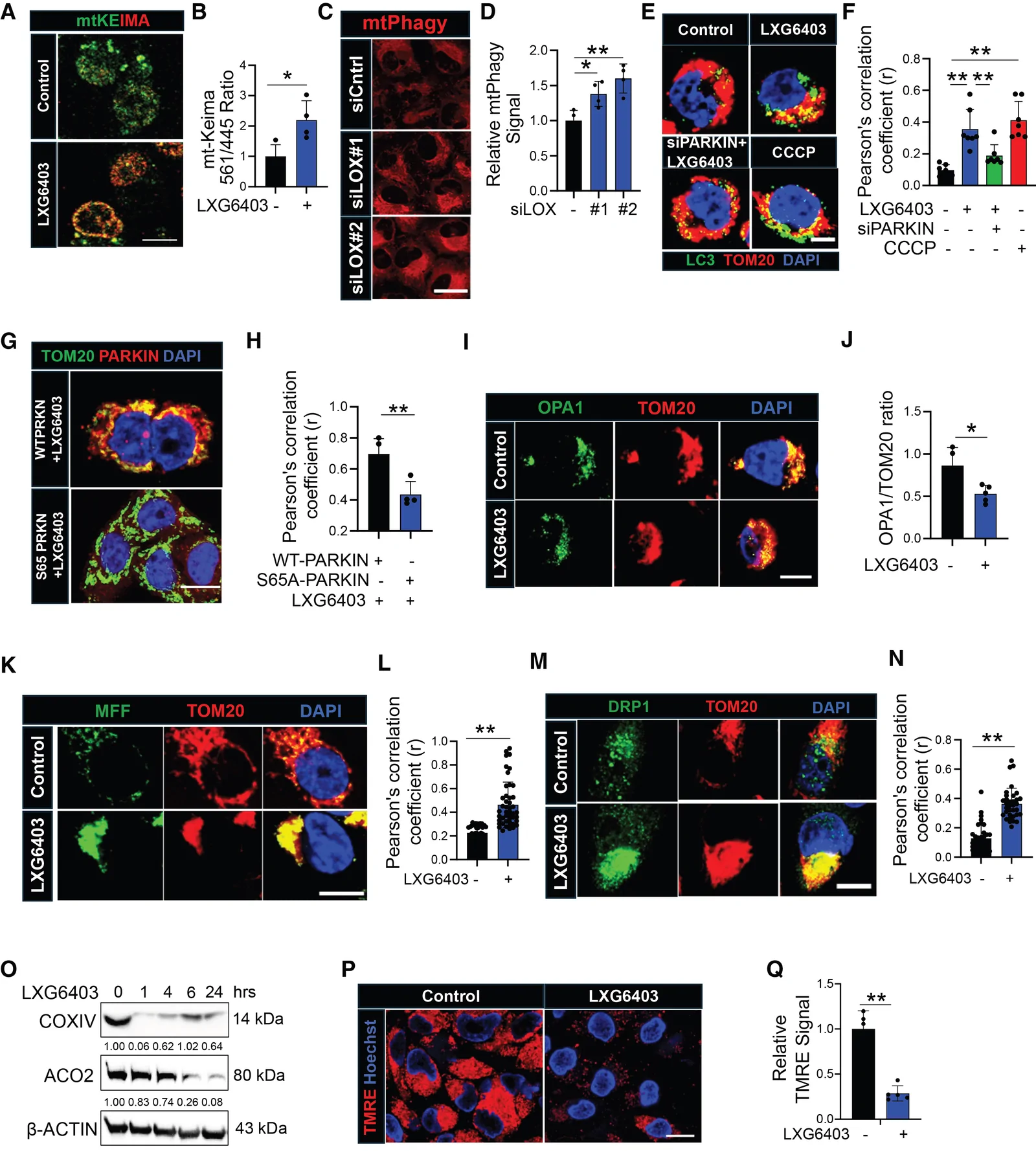
LOX inhibition impairs mitochondrial dynamics, leading to PARKIN-mediated mitophagy (A and B) Representative images of BT549 cells transfected with the mt-Keima vector and treated with LXG6403 (15 μM) (A) and its quantification (B); the shift of red (acidic) over green (neutral) signal denotes elevated mitophagy flux (*n* = 4 images). Scale bars: 25 μm. (C and D) Representative images of mtPhagy from siLOX-transfected BT549 cells (C) and its quantification (D) (*n* = 4 images). Scale bars: 25 μm. (E) IF staining of LC3, TOM20, and DAPI in MDA-MB-231 cells upon LXG6403 (15 μM) for 1 h with or without siPARKIN. Scale bars: 10 μm. (F) Quantification of the co-localization between LC3 and TOM20 from (E) (*n* = 7 images). CCCP is used as a positive control. (G) IF of TOM20, PARKIN, and DAPI showing mitochondrial localization of WT and S65A PARKIN upon LXG6403. Scale bars: 25 μm. (H) Quantification of (G) (*n* = 4 images). (I) IF of OPA1, TOM20, and DAPI in MDA-MB-231 cells treated with LXG6403 (15 μM) for 18 h. Scale bars: 10 μm. (J) Intensity quantification of OPA1 from (I) (*n* = 4–5 images). (K) IF of MFF, TOM20, and DAPI in MDA-MB-231 cells treated with LXG6403 (15 μM) for 18 h. Scale bars: 10 μm. (L) Quantification of co-localization between MFF and TOM20 from (K) (*n* = 46–51 cells). (M) IF of DRP1, TOM20, and DAPI in MDA-MB-231 cells treated with LXG6403 (15 μM) for 18 h. Scale bars: 10 μm. (N) Quantification of co-localization between DRP1 and TOM20 from (M) (*n* = 34 cells). (O) WB of ACO2 and COXIV upon LXG6403 in a time-dependent manner. (P and Q) Representative TMRE images (P) and its quantification (Q) in MDA-MB-231 cells treated with LXG6403 (15 μM) (*n* = 5 images). Scale bars: 25 μm. Data are presented as mean values ± SD. *p* values were calculated with unpaired, two-tailed Student’s *t* test (B, D, F, H, J, L, N, and Q). ∗*p* < 0.05; ∗∗*p* < 0.01.

To test if LOX inhibition impacts mitochondrial organization and mitophagy, regulated by PARKIN,^37^^,^^38^ we assessed mitophagy flux with mt-Keima and MtPhagy^39^ and showed an increase in LXG6403-treated (Figures 4A and 4B) and siLOX-transfected (Figures 4C and 4D) cells. LC3/p62 turnover is increased upon LXG6403 (Figure S3A). LXG6403 promoted PARKIN redistribution to TOM20-positive mitochondria (Figures S3B and S3C) and increased co-localization of LC3-TOM20, which is attenuated by siPARKIN (Figures 4E and 4F), indicative of PARKIN-mediated mitophagy. Wild-type (WT) PARKIN was recruited to mitochondria upon LOX inhibition, whereas the S65A mutant failed to do so (Figures 4G, 4H, S3D, and S3E), suggesting that LOX-inhibition-induced mitophagy depends on PINK1-mediated PARKIN phosphorylation.

Mitochondrial fusion is crucial for mitochondrial integrity.^14^ LOX inhibition decreased OPA1 and increased MFF and DRP1 distribution and co-localization with mitochondria, indicative of reduced fusion and increased fission (Figures 4I–4N). As a result of excessive mitophagy and reduced mitochondrial fusion, LOX inhibition blocked OXPHOS, as determined by reduced protein levels of the TCA cycle protein ACO2 and a member of the electron transport chain COXIV over time (Figure 4O) and reduction of mitochondrial membrane potential (Figures 4P, 4Q, S3F, and S3G). Of note, PARKIN recruitment to mitochondria and mitophagy were unaffected upon overexpressing a degradation-resistant ODD mutant of HIF-1α^40^ (HIF1αΔODD) (Figures S3H–S3M), suggesting that LOX triggers mitophagy independent of the LOX-HIF-1α-glycolysis axis. Taken together, we showed that LOX inhibition activates PARKIN-mediated mitophagy, impairs mitochondrial fusion, and reduces OXPHOS.

### LOX promotes MERCs by preventing PARKIN-mediated VDAC1 degradation and increases mitochondrial Ca^2+^ transport by interacting with HSP90

**Figure 5:**
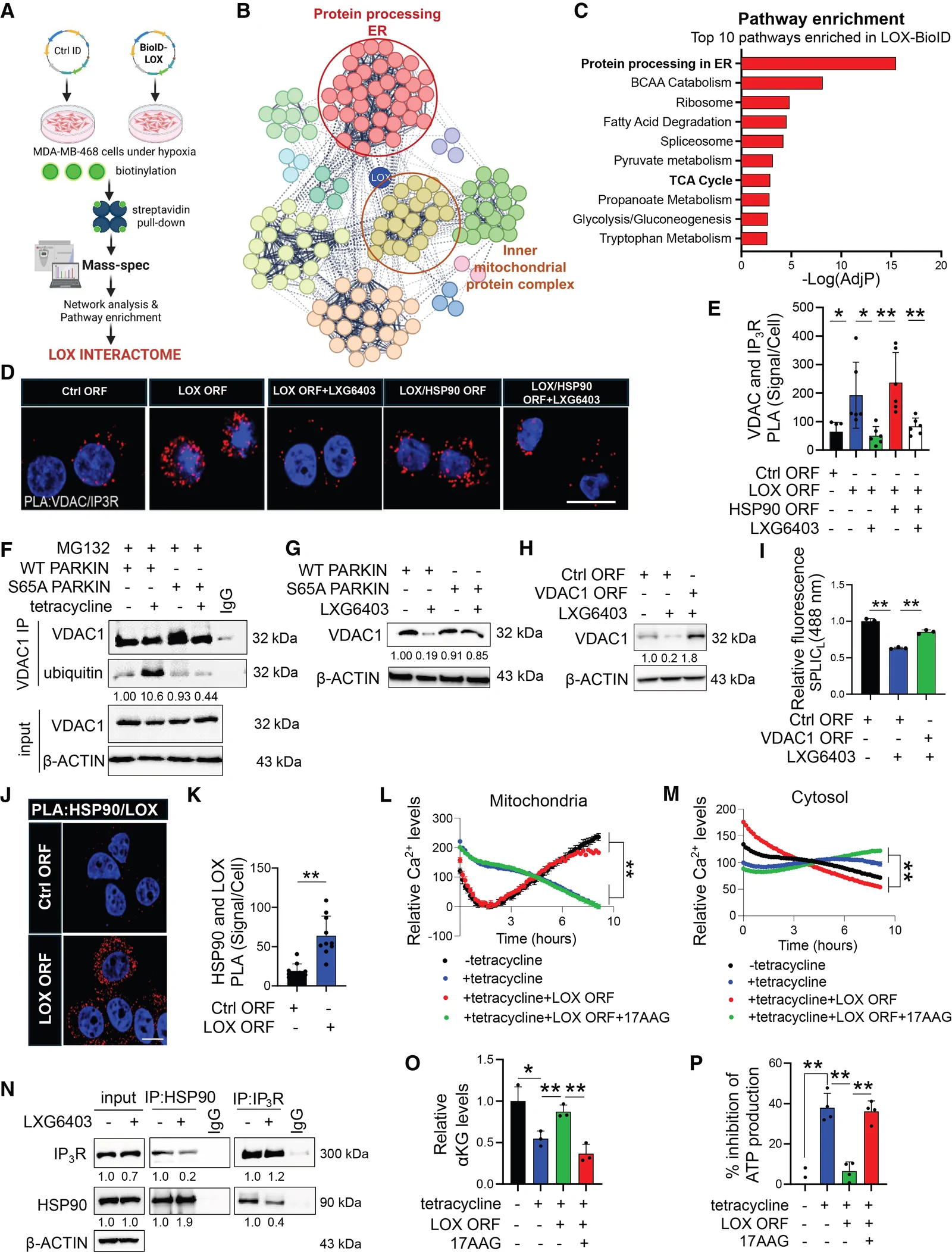
LOX promotes MERCs by preventing PARKIN-mediated VDAC1 degradation and increases mitochondrial Ca^2+^ transport by interacting with HSP90 (A) Experimental pipeline of LOX-BioID in MDA-MB-468 cells (*n* = 3 biological replicates). (B) Network analysis of the LOX interactome identified by BioID followed by mass spectrometry. (C) Pathway enrichment analysis of LOX-interactome. (D) PLA of VDAC1 and IP_3_R in MDA-MB-468 cells overexpressing LOX and/or HSP90 upon LXG6403. Scale bars: 25 μm. (E) Quantification of PLA in (D) (*n* = 6 images). (F) VDAC1 ubiquitination in cells expressing WT or S65A PARKIN upon tetracycline and MG132. (G) WB of VDAC1 in WT and S65A PARKIN-expressing cells upon LXG6403. (H) WB of VDAC1 in Ctrl or VDAC1 ORF-transfected Hs578T cells treated with LXG6403. (I) Quantification of MERC formation in Ctrl or VDAC1 ORF-transfected Hs578T cells treated with LXG6403 (*n* = 3 technical replicates). (J) PLA of LOX and HSP90 in LOX-overexpressing MDA-MB-468 cells. Scale bars: 25 μm. (K) Quantification of PLA in (H) (*n* = 8 images). (L and M) Mitochondrial (L) and cytosolic (M) Ca^2+^ levels upon tetracycline inducible LOX knockdown with or without LOX reconstitution or 17-AAG. (*n* = 3 technical replicates). (N) Reciprocal co-IP showing the interaction between HSP90 and IP_3_R in BT549 cells upon LXG6403. (O) Relative αKG levels in cells from (L) (*n* = 3 technical replicates). (P) % inhibition of ATP production in cells from (L) (*n* = 4 technical replicates). Data are presented as mean values ± SD. *p* values were calculated with unpaired, two-tailed Student’s *t* test (E, I, K, L, M, O, and P). ∗*p* < 0.05; ∗∗*p* < 0.01.

Unbiased LOX interactome analysis by BioID (Figures 5A, 5B, and S4A) revealed enrichment of ER protein processing and inner mitochondrial protein complex components among LOX interactors (Figures 5C and S4B), suggesting MERC regulation, which are essential for lipids and Ca^2+^ transport between the ER and mitochondria, thereby fueling OXPHOS.^12^ In addition, oxidoreductase-related ECM and cell-surface-associated processes were enriched within the LOX interactome, consistent with both redox regulation and canonical LOX functions. Utilizing SPLICS^41^ system demonstrated an increase in both short- and long-distance MERCs upon LOX overexpression, which was reversed by LXG6403 (Figures S4C–S4F), suggesting LOX regulates MERCs. This was further validated by increased interaction between IP_3_R and VDAC1 (MERC mediators) upon LOX overexpression, which was inhibited by LXG6403 (Figures 5D, 5E, S4G, and S4H).

As an underlying mechanism of LOX-regulated MERCs, we showed that LOX inhibition induces ubiquitin-mediated degradation of VDAC1 (Figures 5F, 5G, S5A, and S5B). Of note, other MERC-related proteins were not affected by LXG6403 (Figure S5C). Overexpressing LOX rescues VDAC1 expression and MERCs, while re-inducing mitophagy using CCCP restores VDAC1 degradation and MERC disruption (Figures S5B and S5D). Importantly, overexpressing VDAC1 recovered MERCs in the presence of LOX inhibition (Figures 5H, 5I, and S5E), demonstrating its functionality. MERC signature levels were higher in TNBC patient tumors with high LOX expression (Figure S5F), supporting its clinical relevance. Among the ER-mitochondria-related LOX interactors, we validated the interaction between LOX and HSP90, which was reduced by LXG6403 (Figures 5J, 5K, and S5G). Of note, we showed that the interaction of HSP90 with LOX is not affected by PARKIN (Figure S5H), suggesting no competition among interactors.

To determine which form of LOX protein—i.e., full-length proLOX or the C-terminal mature LOX generated upon cleavage by the BMP1 enzyme^42^—interacts with the identified partners, we overexpressed V5-tagged proLOX, mature LOX, and a BMP1 cleavage-resistant LOX (BM1PR LOX) mutant (Figure S5I) in HEK293T cells. ProLOX and the BMP1R LOX, but not mature LOX, bind to HSP90, PARKIN, and PINK1 (Figure S5J), indicating that these interactions occur at region/s within the proLOX, likely the N-terminal domain that is absent in the mature LOX. Notably, proLOX and BMP1R LOX increased the expressions of HIF-1α, GLUT1, and ACO2, while mature LOX did not (Figure S5K). To identify the binding region on LOX, we generated a series of N-terminal deletion mutants of proLOX and performed co-immunoprecipitation. While the 112-168^th^ amino acid (aa) region of LOX was required for PINK1 and PARKIN, the 1-56^th^ aa region was required for HSP90 binding (Figure S5L), in line with the lack of competition between PARKIN and HSP90 for LOX binding (Figure S5H). Of note, all the truncated vectors retain cellular enzymatic activity (Figure S5M).

Since LOX is canonically a secreted protein, we tested if the intracellular proLOX is secreted and then internalized. Conditioned media from HEK293T cells overexpressing proLOX, mature LOX, BMP1R LOX, and the signal peptide-deficient LOX construct (SP LOX), which is retained intracellularly and not secreted (Figure S6A), were transferred to low LOX-expressing MDA-MB-468 cells (Figure S1A). We showed that proLOX, mature LOX, and BMP1R LOX are internalized, while SP LOX is not (Figures S6B and S6C), suggesting that cells can secrete and internalize proLOX. To uncover the internalization mechanisms, we used endocytosis inhibitors. Dynasore, a dynamin inhibitor,^43^ did not alter the uptake of proLOX, BMP1R LOX, or mature LOX (Figures S6D and S6E). Pitstop2, a clathrin/AP2-mediated endocytosis inhibitor,^44^ significantly reduced proLOX and BMP1R LOX internalization but not mature LOX uptake (Figures S6F and S6G), while cytochalasin D, a macropinocytosis inhibitor, blocked mature LOX internalization, but not proLOX and BMP1R LOX uptake (Figures S6H and S6I). Further validating these, both the recombinant proLOX and mature LOX were efficiently internalized (Figures S7A and S7B). Of note, we validated the expected size and structural integrity of recombinant proteins (Figure S7C) and showed increased lysyl oxidase activity in recipient cell pellets (Figure S7D). Furthermore, internalized proLOX was detected in close proximity to the endosomal and lysosomal compartments and mitochondria (Figures S7E–S7H), consistent with intracellular trafficking of proLOX to engage with the PINK1/PARKIN pathway. Overall, proLOX is the major form interacting with the intracellular binding partners to facilitate metabolic/redox reprogramming, and it is primarily internalized via clathrin/AP2-mediated endocytosis.

To test if LOX fuels mitochondrial Ca^2+^ transfer through MERCs via interacting with HSP90, involved in mitochondrial Ca^2+^ transfer,^45^ we utilized the ER and mitochondria-specific reporters.^46^^,^^47^ Tetracycline-inducible LOX knockdown increased ER Ca^2+^ and reduced mitochondrial Ca^2+^ levels, while reconstitution of LOX fully restored mitochondrial and inhibited cytoplasmic Ca^2+^ levels (Figures 5L, 5M, and S8A). Notably, restoring MERCs via overexpressing VDAC1 in the presence of LOX inhibition (Figures 5H and 5I) partially restores mitochondrial Ca^2+^ (Figure S8B), while inhibiting HSP90 blocks it (Figures 5L, 5M, and S8A), suggesting that LOX facilitates HSP90-driven mitochondrial Ca^2+^ transfer. LXG6403 inhibits the interaction of HSP90 with the ER Ca^2+^ release channel IP_3_R as a potential mechanism of LOX-mediated mitochondrial Ca^2+^ transfer (Figure 5N). Supporting this, mitochondrial Ca^2+^ signature score was higher in high LOX-expressing TNBC tumors (Figure S8C). LOX inhibition further reduced the TCA intermediate alpha-ketoglutarate (α-KG) (Figure 5O) that was among the downregulated metabolites in metabolic profiling (Figure 2A). This ultimately reduced ATP production, which was reversed by LOX reconstitution and restored upon HSP90 inhibition with 17-AAG (Figure 5P). Furthermore, in line with the partial rescue of mitochondrial Ca^2+^ (Figure S8B), restoring MERCs via VDAC1 overexpression partially rescued αKG (Figure S8D) and ATP production upon LOX inhibition (Figure S8E). Of note, overexpression of HSP90 or its pharmacologic inhibition alone or together with LOX ORF (Figures S4C–S4F) or inhibitor (Figures S8F–S8I) does not alter MERCs, suggesting independency from HSP90. Overall, LOX triggers MERCs via stabilizing VDAC1 in a PARKIN-dependent manner while it induces ER-to-mitochondria Ca^2+^ transfer via interacting with HSP90-IP_3_R.

### LOX inhibition generates vulnerability to targeting DHODH-mediated ferroptosis defense in TNBCs

**Figure 6:**
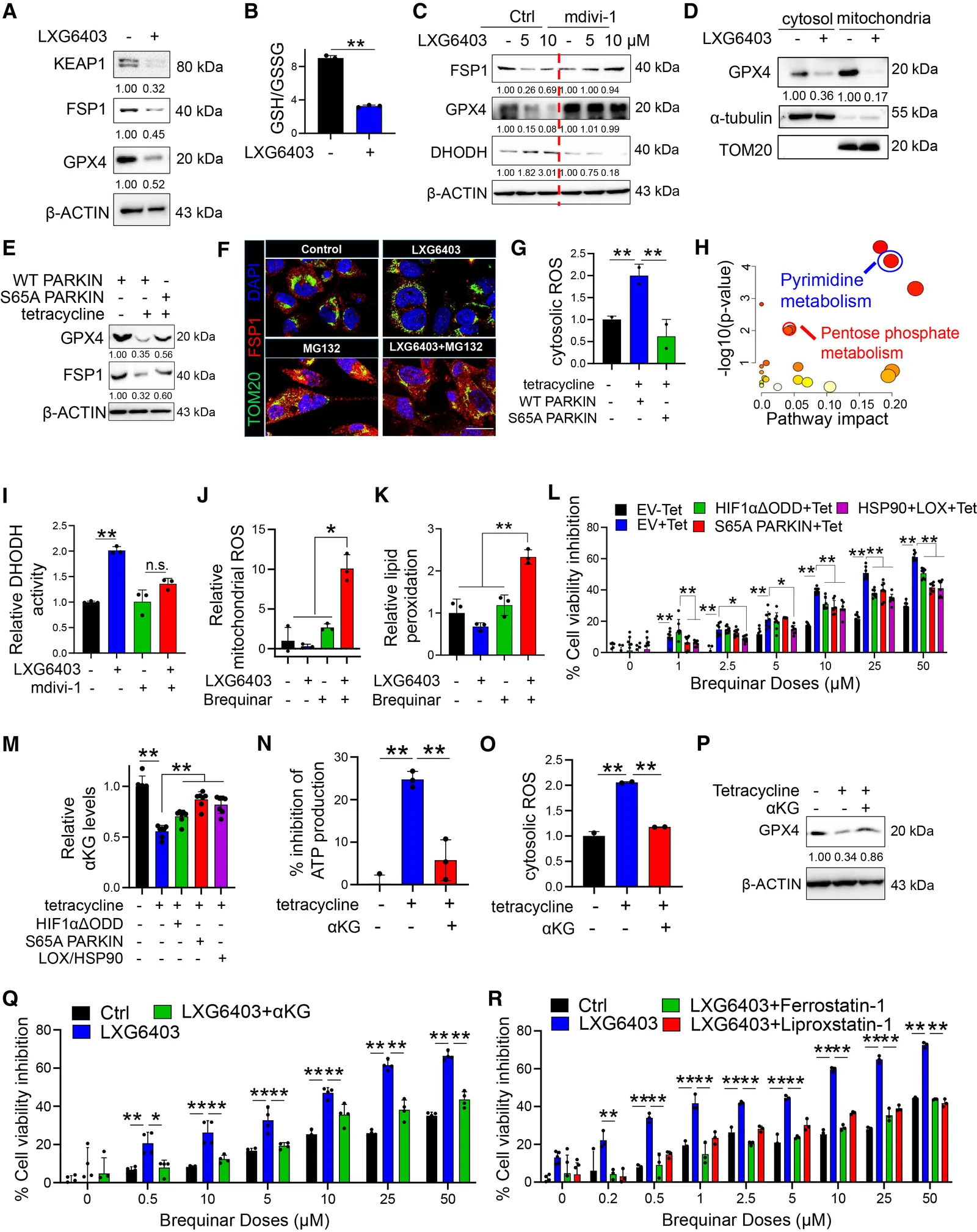
LOX inhibition generates vulnerability to targeting DHODH-mediated ferroptosis defense in TNBCs (A) WB of KEAP1, FSP1, and GPX4 in MDA-MB-231 cells upon LXG6403. (B) Intracellular GSH/GSSG ratio upon LXG6403 (*n* = 3 technical replicates). (C) WB of FSP1, GPX4, and DHODH in MDA-MB-231 cells upon LXG6403 (5 and 10 μM) with or without mdivi-1 (10 μM). (D) WB of GPX4 in cytosolic/mitochondrial fractions upon LXG6403. (E) WB of GPX4 and FSP1 in shLOX cells expressing WT or S65A PARKIN. (F) IF images of FSP1 and TOM20 upon LXG6403 treatment with or without MG132. Scale bars: 25 μm. (G) Cytosolic ROS in shLOX expressing MDA-MB-231 cells transfected with WT or S65A mutant PARKIN (*n* = 2 technical replicates). (H) Network of metabolites upregulated upon LXG6403 (15 μM) for 24 h in MDA-MB-231 cells (*n* = 3 biological replicates). (I) Relative DHODH activity in BT549 cells treated with LXG6403 (15 μM for 24 h), mdivi-1 (10 μM), or both (*n* = 3 technical replicates). (J and K) Relative mitochondrial ROS (J) and lipid peroxidation (K) in MDA-MB-231 cells treated with the combination of LXG6403 (15 μM, 24 h) and brequinar (50 μM, 24 h) (*n* = 3 technical replicates). (L) Percentage viability inhibition upon treatment with multiple doses of brequinar and overexpression of HIF1αΔODD, S65A PARKIN, or LOX/HSP90 in shLOX cells (*n* = 2 biological and 8 technical replicates). (M) Relative αKG levels upon tetracycline-inducible LOX knockdown followed by overexpressing HIF1αΔODD, S65A PARKIN, or LOX/HSP90 (*n* = 2 biological and 6 technical replicates). (N–P) Percentage ATP production inhibition (N) (*n* = 3 technical replicates), cytosolic ROS (O) (*n* = 2 technical replicates), and WB of GPX4 (P) upon tetracycline inducible LOX knockdown in MDA-MB-231 cells. (Q) Percentage viability inhibition following treatment with multiple doses of brequinar in combination with LXG6403 with or without αKG supplementation (*n* = 4–6 technical replicates). (R) Percentage viability inhibition in BT549 cells treated with multiple doses of brequinar in combination with LXG6403 with or without ferroptosis inhibitors ferrostatin-1 (Fer-1, 100 nM) or liproxstatin-1 (Lip-1, 200 nM) for 3 days (*n* = 3–6 technical replicates). Data are presented as mean values ± SD. *p* values were calculated with unpaired, two-tailed Student’s *t* test (B, G, I, J, K, L, M, N, O, Q, and R). ∗*p* < 0.05; ∗∗*p* < 0.01.

Given the enrichment of glutathione metabolism among metabolites downregulated by LOX inhibition (Figures 2A and 2B), we tested whether LOX modulates the antioxidant defense mechanisms. LOX inhibition decreased KEAP1, FSP1, and GPX4 protein levels (Figure 6A), without affecting their mRNA (Figure S9A) and disrupted redox homeostasis, which was rescued by LOX overexpression (Figures 6A, 6B, and S9B). Furthermore, overexpressing proLOX or BMP1R LOX increased GPX4 expression (Figure S5K). Redox homeostasis signature score was higher in high LOX-expressing TNBCs, supporting the clinical relevance (Figure S9C). Notably, the mitophagy inhibitor mdivi-1 rescued their expression in the presence of LXG6403, suggesting mitophagy-dependent degradation (Figure 6C). Furthermore, tetracycline-inducible LOX knockdown also reduced GPX4 and FSP1, which was reversed by LOX reconstitution (Figure S9D). Re-activating mitophagy using CCCP restored GPX4 and FSP1 degradation (Figure S9D). GPX4 can be found in both cytosol and mitochondria. Mitochondrial fractionation showed that LOX inhibition reduces both the cytosolic and mitochondrial GPX4 (Figure 6D), arguing against mitochondrial clearance. GPX4 is ubiquitinated upon LOX inhibition in a PARKIN-dependent manner (Figure S9E). Inhibiting proteasome with MG132, but not lysosome with bafilomycin A1 (Figure S9F), or overexpressing PARKIN S65A (Figure 6E) restored GPX4 levels, indicating PARKIN-mediated proteasomal turnover.

We observed no mitochondrial recruitment of FSP1 upon LXG6403 (Figure 6F), arguing against degradation via mitophagy. Similar to GPX4, FSP1 is also ubiquitinated in a PARKIN-dependent manner (Figure S9G) and degraded via proteasome in a reactive oxygen species (ROS)-dependent manner upon LXG6403 (Figure S9H), suggesting proteasomal degradation due to cytosolic ROS spillover. Indeed, cytosolic ROS increased, while mitochondrial ROS reduced upon LOX inhibition (Figures S9I and S9J), changes that were reversed by LOX overexpression (Figure S9K). Inducing mitophagy partially restored cytosolic ROS (Figure S9K). Consistently, inducible LOX knockdown increased cytosolic ROS in the presence of WT, but not PARKIN, S65A (Figure 6G). Furthermore, mdivi-1 treatment or PARKIN S65A overexpression rescues FSP1 levels upon LOX inhibition (Figures 6C and 6E). Overall, LOX inhibition disrupts redox homeostasis in part by cytosolic ROS spillover and proteasomal degradation of mitochondrial and cytosolic mediators of antioxidant defense.

While LOX inhibition reduced glucose metabolism (Figures 2A and 2B), it increased pentose phosphate and the mitochondria-linked *de novo* pyrimidine biosynthetic pathways (Figure 6H) as potential compensatory responses. Notably, dihydroorotate dehydrogenase (DHODH) is the only mitochondrial enzyme that couples pyrimidine production to mitochondrial redox/respiratory activity, making it highly sensitive to mitochondrial stress and hypoxia.^48^^,^^49^ Of note, pyrimidine salvage enzymes (e.g., UCK1/2, TK1/2, and TYMP), unrelated to mitochondria,^50^ were not enriched among LOX-regulated metabolites. Intriguingly, LOX inhibition significantly increased DHODH activity (Figure 6I) and expression (Figure 6C), reversed by mdivi-1. Combining LOX inhibition with the DHODH inhibitor brequinar^51^ increased mitochondrial ROS and cytosolic iron accumulation (Figures 6J and S9L). These ultimately increased lipid peroxidation (Figure 6K) and caused synergistic cell viability inhibition (Figure S9M). Next, we tested the relative contribution of different LOX-modulated processes to tumor cell fitness. We showed that (1) restoring glycolysis upon overexpressing HIF1αΔODD or (2) blocking PARKIN activation by overexpressing S65A mutant or (3) restoring mitochondrial Ca^2+^ by reconstituting the LOX/HSP90 complex partially rescued cell viability in the presence of LXG6403 and DHODH inhibitor combination (Figure 6L) that correlates with partial rescue of αKG (Figure 6M), known to act as an ROS scavenger,^52^ which is the convergence point of these three arms. Importantly, supplementing LOX-inhibited cells with αKG (Figures S9N and S9O) fully rescued ATP production (Figures 6N and S9P), reduced cytosolic ROS accumulation (Figures 6O and S9Q), and restored GPX4 levels (Figures 6P and S9R) that ultimately restored cell viability (Figure 6Q). Reactivating GPX4 using ferrostatin-1 or liproxstatin-1 also rescued cell viability in the presence of combination therapy (Figure 6R). Together, LOX inhibition compromises canonical ferroptosis defenses by disrupting αKG production upon simultaneous blockage of glycolysis, PARKIN-mediated mitophagy, and disruption of mitochondrial Ca^2+^ transfer, while inducing compensatory DHODH pathway activation, creating a targetable vulnerability to ferroptosis.

### LOX inhibition in combination with a clinical DHODH inhibitor leads to strong tumor growth inhibition in multiple clinically relevant *in vivo* models

**Figure 7:**
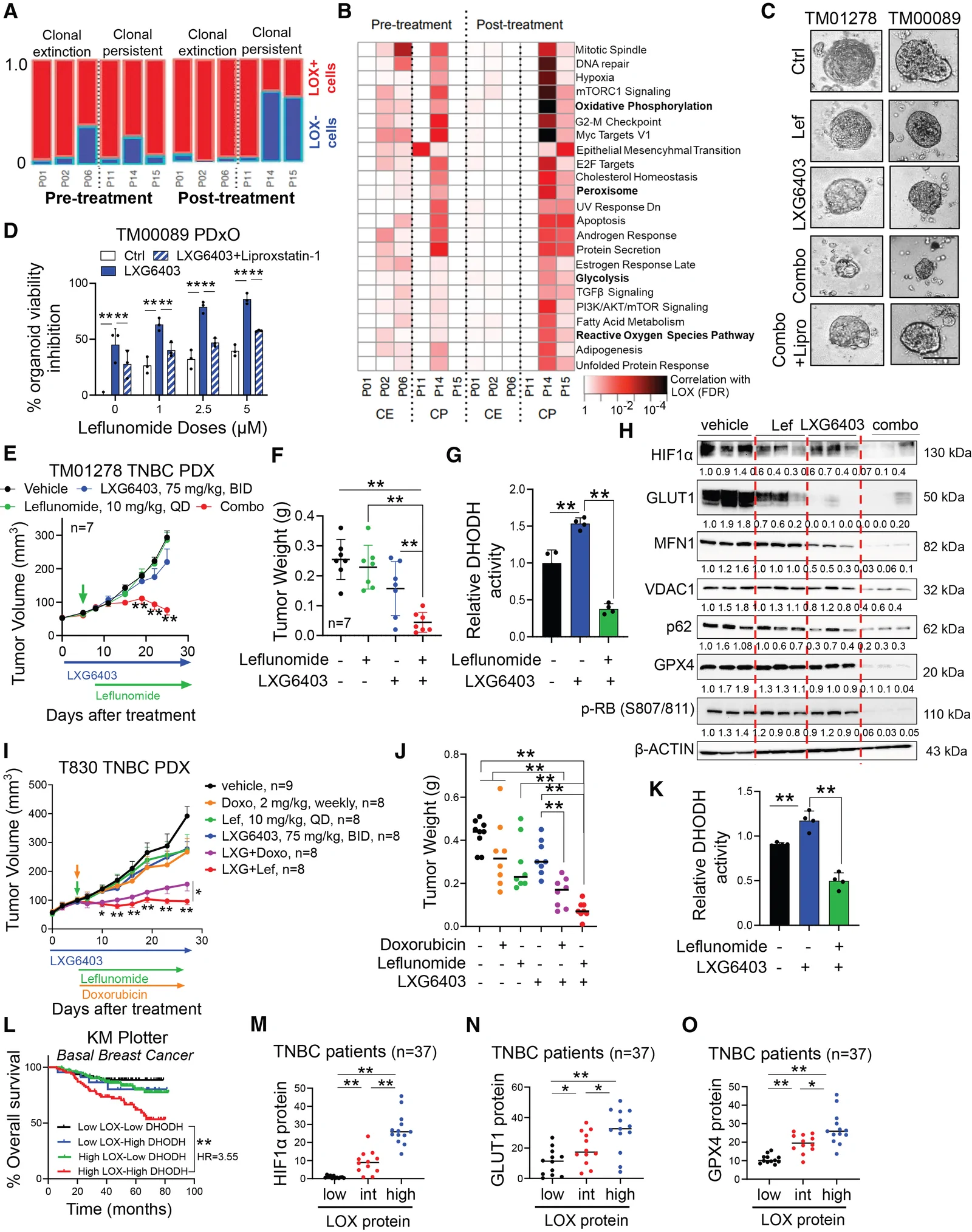
LOX inhibition in combination with a clinical DHODH inhibitor leads to strong tumor growth inhibition in multiple clinically relevant *in vivo* models (A and B) Fraction of LOX+ cancer cells before and after treatment in chemo-responsive (CE) vs. resistant (CP) TNBC patients determined by scRNA-seq data (A) and the pathways enriched among LOX correlating genes (B) (*n* = 6 patients). (C and D) Representative images (C) and % TM00089 organoid viability inhibition upon leflunomide (1, 2.5, and 5 μM) and LXG6403 (10 μM) for 5 days (D) (*n* = 3 technical replicates). Scale bars: 25 μm. (E and F) Tumor volume (E) and weight (F) in TM01278 PDXs treated with vehicle, leflunomide (10 mg/kg, daily, p.o.), LXG6403 (75 mg/kg, bidaily, p.o.), or the combination for 25 days (*n* = 7 mice per group). (G) DHODH activity in tumor lysates from (E) (*n* = 4 tumors). (H) WB of HIF-1α, GLUT1, MFN1, VDAC1, p62, GPX4, and p-RB (S807/811) in tumors from (E) (*n* = 3 tumors). (I and J) Tumor volume (I) and weights (J) of T830 PDX tumors treated with vehicle, LXG6403 (75 mg/kg, bidaily, p.o), leflunomide (10 mg/kg, daily, p.o.), doxorubicin (2 mg/kg, weekly, i.v.), or their combination with LXG6403 for 28 days (*n* = 8–9 mice per group). (K) DHODH activity in tumor lysates from (I) (*n* = 4 tumors). (L) Survival analysis of basal breast cancer patients from the KM Plotter database stratified based on low vs. high LOX and DHODH expression (*n* = 309 patients). (M–O) The mean intensities of HIF-1α (M), GLUT1 (N), and GPX4 (O) proteins in TNBC patient tumors (*n* = 37 patients) stratified based on LOX expression (low, intermediate, and high). Data are presented as mean values ± SD for (D, F, G, J, and K) and as mean values ± SEM for (E and I). *p* values for the bar graphs and dot plots were calculated with the unpaired, two-tailed Student’s *t* test (D, F, G, J, K, M, N, and O). Significance for the tumor volume was calculated using the Wilcoxon rank-sum test (E and I) and the log rank (Mantel-Cox) test for Kaplan-Meier survival analysis (L). ∗*p* < 0.05; ∗∗*p* < 0.01.

High levels of a ferroptosis-inducing gene signature^53^ are associated with better overall survival in chemotherapy-treated TNBC patients (Figure S10A). Importantly, analysis of a published single-cell RNA-seq (scRNA-seq) dataset from chemo-treated TNBC patients^54^ revealed enrichment of glycolysis and OXPHOS in persister subclones with increased LOX expression (Figures 7A and 7B). Notably, ferroptosis-related pathways were also enriched in LOX+ persister cells (Figures 7A and 7B). Integrating genes upregulated in chemoresistant TNBC xenografts that we previously generated^26^ with the list of genes downregulated upon LOX inhibition (Figures 1A and S10B) revealed enrichment of carbon metabolism, HIF-1 signaling, and mitophagy processes (Figure S10C). Importantly, these LOX-regulated chemoresistance-related genes were enriched in LOX-high/GLUT1-rich regions of TNBC PDXs (Figures 1H and S10D), suggesting LOX-dependent alterations in glucose and redox metabolism in chemoresistance.

To test if LOX inhibition confers sensitivity to DHODH-inhibition-mediated ferroptosis *in vivo*, we selected two chemoresistant TNBC PDXs, TM01278 and TM00089,^26^^,^^55^ expressing high-moderate levels of the glucose metabolism and ferroptosis signature scores (Figures S10E and S10F). LOX inhibition combined with clinical DHODH inhibitor leflunomide^56^ in PDX-derived organoids (PDxOs) reduced organoid viability, which was reversed by liproxstatin-1 (Figures 7C, 7D, and S11A). Combination of LXG6403 and leflunomide in chemoresistant TM01278 PDX induced tumor growth inhibition in a chemo-free setting without any significant body weight change (Figures 7E, 7F, and S11B–S11D). Notably, assessments of kidney and liver indices showed no major organ toxicity (Figures S11E and S11F). There was an increase in DHODH activity in tumors upon LXG6403 (Figure 7G), confirming its compensatory activation, which was reversed by combination therapy. Furthermore, combination therapy reduced protein levels of HIF-1α, GLUT1, p62, MFN1, VDAC1, and the G1/S progression marker p-RB (Figure 7H). These were recapitulated in another TNBC PDX model, T830, derived from a patient with recurrent disease following multi-agent chemotherapy, including doxorubicin. In this setting, combination of LXG6403 with leflunomide resulted in better tumor growth inhibition compared to combination of LXG6403 with doxorubicin without major body weight change (Figures 7I, 7J, and S11G). Furthermore, DHODH activity was increased by LXG6403 and reduced upon combination therapy (Figure 7K). Notably, tetracycline-inducible LOX knockdown recapitulated induction of DHODH activity (Figure S11H) and downregulation of protein levels of GLUT1, VDAC1, and GPX4 in tumors (Figure S11I), resulting in significant tumor growth inhibition in combination with leflunomide without major body weight loss (Figures S11J–S11M). Next, to test if LOX inhibition creates vulnerability to DHODH targeting also in an immunocompetent setting, we combined LXG6403 with leflunomide, which resulted in a significant tumor growth inhibition in the 4T1 TNBC model (Figures S12A–S12D).

High levels of both LOX and DHODH is associated with worse overall survival in TNBC patients (Figure 7L). Additionally, mRNA levels of *HIF-1A*, *GLUT1*, and *GPX4* were higher in TNBC patients expressing higher *LOX* (Figures S13A–S13C). Immunofluorescence (IF) staining of TNBC patient tumors (*n* = 37) showed higher expressions of HIF-1α, GLUT1, and GPX4 proteins in LOX-high patient tumors (Figures 7M–7O and S13D). On the other hand, there was no association of LOX between mRNA (Figures S14A–S14C) and protein (Figures S14D–S14G) levels of other potential regulators: TFAP2A, the HIF-1α cofactor^57^; GLUT4, a glucose transporter; and SLC7A11 (xCT), a ferroptosis inhibitor. Together, LOX inhibition generates DHODH dependency in TNBCs, and the combination of LOX and DHODH inhibition presents an attractive therapeutic strategy to block TNBC tumor growth in a chemo-free setting.

## Discussion

TNBCs are characterized by high metabolic heterogeneity and plasticity, contributing to their aggressiveness and therapy resistance. Thus, identification of therapeutic vulnerabilities to improve clinical outcomes is urgently needed. Here, we identify non-canonical functions of LOX in promoting glucose metabolism, inhibiting mitophagy, and sustaining redox homeostasis and show that inhibiting LOX generates a targetable vulnerability to DHODH-inhibition-mediated ferroptosis. Importantly, we found that LOX inhibition (1) disrupts the interaction between LOX and PINK/PARKIN complex, leading to (2) PINK-mediated phosphorylation and activation of PARKIN. (3) Activated PARKIN, on one hand, causes ubiquitination and degradation of HIF-1α, thus reducing glycolysis, and (4) on the other hand, it is recruited to mitochondria to induce mitophagy, indicating coordination between metabolic regulation and mitochondrial quality control. (5) Activated PARKIN on the mitochondria ubiquitinates and degrades VDAC1, disrupting MERCs. (6) At the same time, disruption of the LOX-HSP90-IP_3_R complex impairs mitochondrial Ca^2+^ transfer. (7) Disruption of the downstream glycolysis, mitophagy, and mitochondrial Ca^2+^ transfer arms contribute to αKG reduction, resulting in reduced OXPHOS and ATP production. (8) Decrease in αKG further leads to increased cytosolic ROS and ubiquitin-mediated proteasomal degradation of GPX4 and FSP1, culminating in disrupted redox homeostasis. (9) This redox stress induces compensatory DHODH dependency, which can be therapeutically targeted. Notably, pharmacologic or genomic LOX inhibition combined with a clinical DHODH inhibitor using a “one-two punch” approach inhibited tumor growth in chemo-free setting without major toxicity *in vivo*. Importantly, LOX expression strongly correlates with HIF-1α, GLUT1, and GPX4 in TNBC patients, supporting the clinical relevance of LOX-driven metabolic redox reprogramming.

A tight coordination exists between mitophagy and mitochondrial homeostasis contributing to cancer.^58^ However, little is known about the molecular mechanisms of mitophagy-regulated mitochondrial dynamics and their contribution to energy metabolism. Our data provide key insights into the dynamics of mitochondrial homeostasis in TNBC through LOX-mediated suppression of mitophagy that not only preserves TCA intermediates but also promotes mitochondrial fusion and protects mitochondrial membrane integrity. Mitochondrial fusion may serve as a compensatory rescue mechanism incorporating dysfunctional mitochondria into mitochondrial pool; thus, inhibiting mitophagy may ultimately lead to therapy resistance. Here, we showed a reciprocal regulation of mitochondrial fusion by mitophagy where LOX inhibition-mediated mitophagy reduces mitochondrial fusion, which may further contribute to mitophagy induction in a feedforward loop. This may represent a way to inhibit the growth of highly aggressive tumors, including chemoresistant TNBCs.

MERCs are highly dynamic structures controlling lipid and Ca^2+^ homeostasis and mitochondria metabolism.^12^ Key tethering factors like IP_3_R-VDAC1^59^ and MFN2^60^ bring domains of mitochondria and ER in close proximity, forming MERCs to allow efficient transport of substances, including lipids and Ca^2+^ between the two organelles. However, little is known about the regulation and function of MERCs during metabolic stress. Our findings suggest that LOX facilitates MERC formation and Ca^2+^ transfer from ER to mitochondria via promoting HSP90-IP_3_R interaction to produce energy. During PARKIN-mediated mitophagy, tethering factors including VDAC1 are ubiquitinated and degraded, thus keeping ER and mitochondria apart to allow engulfment of damaged mitochondria.^61^ Indeed, we showed that LOX inhibition triggers PARKIN-mediated ubiquitination and degradation of VDAC1, thus disrupting MERC formation. Notably, we found that the LOX-HSP90-IP_3_R complex promotes mitochondrial Ca^2+^ transfer without affecting the LOX-MERC axis, suggesting that LOX sustains mitochondrial homeostasis at multiple layers from MERC formation to ion transport across mitochondria to robustly facilitate energy production.

Mitochondrial GPX4 and DHODH are the two main defense mechanisms suppressing mitochondrial lipid peroxidation and ROS accumulation, while FSP1 acts in parallel to GPX4 in cytoplasm.^62^ However, little is known about how mitophagy regulates these compensatory arms to generate vulnerability to ferroptosis. Here, we show that LOX inhibition reduces glutathione metabolism and downregulates both the GPX4 and FSP1 arms of the redox defense, while triggering DHODH activity as a potential compensatory mechanism, thereby creating a targetable vulnerability. DHODH inhibition can induce mitochondrial lipid peroxidation and ferroptosis in cancer cells with low levels of GPX4,^63^ while inactivation of both the GPX4 and DHODH arms is required when GPX4 levels are high.^64^ Our findings suggest that by downregulating GPX4 and FSP1, LOX inhibition generates vulnerability to DHODH-inhibition-mediated ferroptosis. This “one-two punch” strategy represents an effective approach for ferroptosis induction in highly aggressive chemoresistant TNBCs.

Given the importance of energy metabolism and antioxidant defense in tumorigenesis, proteins/enzymes having roles in metabolic pathways are prime candidates for therapeutic targeting. For instance, the RB1-GLUT1 metabolic axis was identified as a targetable pathway in TNBC with modest tumor growth inhibition upon GLUT1 targeting using BAY-876.^65^ Furthermore, dose-limiting toxicities exist for BAY-876, and the potential effects of GLUT1 inhibitors in humans are uncertain. Notably, due to the absence of approved ferroptosis-based drugs, its translational potential remains unknown.^2^ For instance, GPX4 inhibitors have shown some preclinical efficacy in TNBC; however, poor pharmacological properties pose challenges for clinical translation.^8^ DHODH has recently become an attractive anti-cancer drug target. The DHODH inhibitor leflunomide, clinically approved for rheumatoid arthritis, is currently being tested in blood cancers (NCT06923488) and solid cancers, e.g., pancreatic cancer (NCT06454383) in combination with standard therapies. We demonstrate that targeting LOX generates vulnerability to DHODH inhibition, suggesting that repurposing leflunomide could be a clinically viable and safe approach to target highly aggressive, chemoresistant TNBCs.

LOX is crucial for chemoresistance in desmoplastic tumors, e.g., pancreatic tumors^66^^,^^67^ known to be dependent on mitochondrial metabolism^68^ and *de novo* pyrimidine biosynthesis.^69^ However, single-agent DHODH inhibitors have shown only partial responses in PDAC.^70^ Thus, targeting LOX may serve as a generalizable therapeutic approach increasing dependency to DHODH inhibitors in desmoplastic tumors. Indeed, there have been efforts to develop safer inhibitors targeting LOX family members. These include the pan-lysyl oxidase inhibitor, PXS-5505, which was well tolerated in a phase I study in healthy volunteers, demonstrating the safety of targeting LOX enzymes. PXS-5505 has also shown clinical efficacy in patients with advanced myelofibrosis in phase I/IIa trial (NCT04676529).^71^ However, there are also a few limitations reported for the clinical use of LOX family inhibitors. For instance, simtuzumab, a humanized monoclonal antibody targeting LOXL2, showed no clinical benefit in the first- and second-line settings in solid tumors (NCT01672866, NCT01672879, and NCT01769196). However, it was recently reported that this may not be related to the target but instead could be attributed to the inability of the antibody to effectively inhibit LOXL2 catalytic activity.^72^ LXG6403 potently inhibits LOX activity *in vitro* and in solid tumors, has favorable drug-like features, is well-tolerated,^28^ and has potential to become a feasible strategy to target highly aggressive desmoplastic tumors in combination with clinical DHOD inhibitor.

In summary, our study identified non-canonical functions of LOX orchestrating glucose metabolism, mitophagy, and ferroptosis in TNBC. We demonstrated the therapeutic potential of targeting LOX in combination with DHODH inhibitors to eradicate highly aggressive TNBC tumors that could pave the way for clinical testing of LOX inhibitors in combination with DHODH inhibitors.

### Limitations of the study

Our study highlights the role of LOX in promoting glucose metabolism, inhibiting mitophagy, and sustaining redox homeostasis. Since the canonical function of LOX is collagen crosslinking, future studies may investigate whether metabolic changes driven by LOX also regulate ECM. Although we demonstrated that PINK1 is a direct substrate of LOX and that LOX-mediated oxidation suppresses PINK1 kinase activity, the precise lysine residue(s) targeted by LOX remains to be determined by future proteomic and structural studies. Although we demonstrated LOX-mediated disruption of mitochondrial homeostasis creates vulnerability to ferroptosis in TNBC, future studies may determine if this is applicable to other LOX-high tumor types.

## Resource availability

### Lead contact

Requests for further information and resources should be directed to and will be fulfilled by the lead contact, Ozgur Sahin (sahin@musc.edu).

### Materials availability

This study did not generate new unique reagents. Plasmids generated in this study are available from the lead contact with a completed Materials Transfer Agreement.

### Data and code availability

All data relevant to the study are included in the article or uploaded as supplemental information. RNA-seq and spatial transcriptomics data generated during this study have been deposited at the National Center for Biotechnology Information (NCBI) Gene Expression Omnibus (GEO) Databases: GSE312563 and GSE338674, respectively. Mass spectrometry proteomics data have been deposited at the ProteomeXchange Consortium via the PRIDE^73^ partner repository (Database: PXD071741). Metabolic profiling data have been deposited to NIH Common Fund’s National Metabolomics Data Repository (NMDR) website, the Metabolomics Workbench, https://www.metabolomicsworkbench.org,^74^ with Study ID: ST005017. These data are publicly available. This paper analyzes existing, publicly available data from Databases: METABRIC,^75^ TCGA (https://www.cancer.gov/tcga), the GEO Databases: GSE103091,^76^ GSE31448,^77^ and GSE76124,^78^ and the KM Plotter database.^79^ This paper does not report original code. Uncropped WBs have been deposited at Mendeley (Mendeley Data: https://doi.org/10.17632/44vvkgby2v.1). Any additional information required to reanalyze the data reported in this work is available from the lead contact upon request.

## Acknowledgments

We thank the members of Ozgur Sahin laboratory for their discussion and advice. Metabolic profiling was performed by Metabolomics Core Facility at Robert H. Lurie Comprehensive Cancer Center (NCI CCSG P30 CA060553) of Northwestern University. We also thank Dr. Robert P. Mecham for the LOX^ko/ko^ mice.^30^ This work was supported by research funding from the 10.13039/100000002NIH (R01CA267101 to O. Sahin) and in part from the NIH (R01CA251374 to O. Sahin), SmartState Endowment in Lipidomics and Drug Discovery (O. Sahin), the NIH (R01DE016572 to B.O.), and 10.13039/100006806Hollings Cancer Center, MUSC (Postdoctoral Fellowship to O. Saatci and M.C.). The animal core facility was supported by the NIH (C06 RR015455). The Biostatistics Shared Resource, Hollings Cancer Center was supported by the NIH (P30 CA138313). The Zeiss 880 microscope was funded by a Shared Instrumentation grant (S10 OD018113). The proteomics was performed in the MUSC Mass Spectrometry Facility and supported, in part, by NIH grants S10 OD028692, P30 CA138313, and P20 GM103542.

## Author contributions

Conceptualization, O. Saatci, B.U., M.C., A.H.R., and O. Sahin; experiments, O. Saatci, B.U., and M.C.; methodology, O. Saatci, B.U., H.K., C. Martinez, E.P., N.O., M.T.S., K.S., A.H.R., C.N.R., C. McInnes, M.A., and O. Sahin; investigation, O. Saatci, B.U., Y.R., H.S.N., J.J.L., B.O., M.P., and O. Sahin; *in vivo* experiments, O. Saatci, B.U., and O.S.S.; data analyses, O. Saatci, B.U., A.N., A.H.-C., E.Z., and J.R.B.; statistical analysis, J.-S.A. and E.G.H.; writing – original draft, O. Saatci and B.U.; writing – review and editing, L.E.B., M.B., Y.R., H.S.N., J.J.L., B.O., M.P., and O. Sahin; funding acquisition, O. Sahin; resources, H.K. A.A.-M., M. Buchanan, M. Basik, C. Martinez, E.P., N.O., K.S., G.D., C.N.R., C. McInnes, M.P., and O. Sahin.

## Declaration of interests

O. Sahin is the co-founder and manager of OncoCube Therapeutics LLC, founder and president of LoxiGen, and member of scientific advisory board of Coiled Therapeutics plc. O. Saatci, M.C., K.S., A.-H.R., C.N.R, C. McInnes, and O. Sahin are inventors on patents related to LXG6403.

## STAR★Methods

### Key resources table

**Table:** 

REAGENT or RESOURCE	SOURCE	IDENTIFIER
**Antibodies**		

Please see Table S1 for the dilutions		
GLUT1	Cell signaling	Cat#12939: RRID: AB_2687899
HIF-1α	Abcam	Cat#179483: RRID: AB_2732807
His-Tag	Invitrogen	Cat#MA1-21315MG; RRID: AB_2536982
HA	Invitrogen	Cat#26183: RRID: AB_10978021
PARKIN	Proteintech	Cat#14060-I-AP: RRID: AB_2882028
PARKIN	Santa Cruz	Cat#sc-32282: RRID: AB_628104
β-ACTIN	Sigma	Cat#A2228; RRID: AB_476697
COXIV	Cell Signaling	Cat#4850; RRID: AB_2085424
ACO2	Cell Signaling	Cat#6922: RRID: AB_10828218
LC3	Cell signaling	Cat#12741: RRID: AB_2617131
p62	Cell signaling	Cat#39749: RRID: AB_2799160
HSP90	Santa Cruz	Cat#sc-13119: RRID: AB_675956
TOM20	Cell signaling	Cat#42406: RRID: AB_2687663
TOM20	Santa Cruz	Cat#sc-136211: RRID: AB_2207538
OPA1	Cell signaling	Cat#80471: RRID: AB_2734117
VDAC1	Proteintech	Cat#10866-1-AP: RRID: AB_2257153
IP3R	Santa Cruz	Cat#sc-377518: RRID: AB_2637028
MFF	Cell Signaling	Cat#84580: RRID: AB_2728769
DRP1	Cell Signaling	Cat#8570: RRID: AB_10950498
HSPA5	Santa Cruz	Cat#sc-166490: RRID: AB_2264290
KEAP1	Cell Signaling	Cat#8047: RRID: AB_10860776
FSP1	Cell Signaling	Cat#24972: RRID: AB_3090192
GPX4	Proteintech	Cat#67763-1-IG: RRID: AB_10898356
Ubiquitin	Cell Signaling	Cat#58395: RRID: AB_3075532
DHODH	Proteintech	Cat#14877-1-AP: RRID: AB_2091723
MFN1	Cell Signaling	Cat#14739: RRID: AB_2744531
p-RB	Cell Signaling	Cat#8516: RRID: AB_11178658
LOX	Cell Signaling	Cat#39774: RRID: AB_3741737
VHL	Cell Signaling	Cat#68547: RRID: AB_2716279
Phospho PARKIN (S65)	Cell Signaling	Cat#36866: RRID: AB_3741738
PINK1	Cell Signaling	Cat#6946: RRID: AB_11179069
GLUT4	Abcam	Cat#ab33780: RRID: AB_2191441
TFAP2	Abcam	Cat#ab108311: RRID: AB_10861200
xCT	Novus	Cat#NBP3-33104: RRID: AB_3741739
VGLL3	Thermo Scientific	Cat#PA570096: RRID: AB_2689281
V5 Tag	Invitrogen	Cat#R960-25: RRID: AB_2556564
TOM20	AvesLabs	Cat#TOM20-00100: RRID: AB_3751517
EEA1	Abcam	Cat# ab206280: RRID: AB_3665450
LAMP1	Abcam	Cat#ab24871: RRID: AB_2134490
TUBA4A	Origene	Cat#AB0046-200: RRID: AB_3751516
PACS2	Thermo Scientific	Cat#PA5-72866: RRID: AB_2718720
MFN2	Cell Signaling	Cat#11925: RRID: AB_2750893
GRP75	Proteintech	Cat# 14887-I-AP: RRID: AB_2934906
ORP5	Proteintech	Cat# 67913-I-Ig: RRID: AB_3085041
HRP-linked anti-mouse IgG	Cell Signaling	Cat#7076: RRID: AB_330924
HRP-linked Anti-rabbit IgG	Cell Signaling	Cat#7074: RRID: AB_2099233
Alexa Fluor 488 anti-mouse	Life Technologies	Cat#A-11001: RRID: AB_2534069
Alexa Fluor 488 anti-rabbit	Life Technologies	Cat#A-11034: RRID: AB_2576217
Alexa Fluor 647 anti-mouse	Life Technologies	Cat#A-31571: RRID: AB_162542
Alexa Fluor 647 anti-rabbit	Life Technologies	Cat#A-31573: RRID: AB_2536183
Donkey anti-Goat IgG H&L (Alexa Fluor® 488)	Abcam	Cat#ab150169: RRID: AB_2636803
Goat Anti-Chicken IgY H&L (Alexa Fluor® 488)	Abcam	Cat#ab150129: RRID: AB_2687506

**Bacterial and virus strains**		

CMV-Mito4x-GCaMP6f	Addgene	Cat#127870: RRID: Addgene_127870
ER-GCamP6-150	Addgene	Cat#86918: RRID: Addgene_86918
GCaMP6F	Addgene	Cat#163045: RRID: Addgene_163045
MCS-BioID2-HA	Addgene	Cat#74224: RRID: Addgene_74224
SPLICS Mt-ER Long P2A	Addgene	Cat#164107: RRID: Addgene_164107
SPLICS Mt-ER Short P2A	Addgene	Cat#164108: RRID: Addgene_164108
p(HA)HIF1alpha (P402A,P564A)	Addgene	Cat#52636: RRID: Addgene_52636
pCherry.90alpha Vector	Addgene	Cat#108222: RRID: Addgene_108222
eGFP-pk-S65A	Addgene	Cat#115619: RRID: Addgene_115619
pEGFP-parkin WT	Addgene	Cat#45875: RRID: Addgene_45875
psPAX2	Addgene	Cat#12260: RRID: Addgene_12260
pMD2.G	Addgene	Cat#12259: RRID: Addgene_12259
LOX-BioID	In this paper	In this paper
pcDNA-proLOX-His-V5	In this paper	In this paper
pcDNA-matureLOX-His-V5	In this paper	In this paper
pcDNA-LOX-BMP1-His-V5	In this paper	In this paper
pcDNA-SP-LOX-His	In this paper	In this paper
pCMV-proLOX-FLAG	In this paper	In this paper
pCMV-Δ1-56proLOX-FLAG	In this paper	In this paper
pCMV-Δ1-112proLOX-FLAG	In this paper	In this paper
pCMV-Δ1-168proLOX-FLAG	In this paper	In this paper

**Biological samples**		

Human TNBC patient tumor samples	Grusso et al.^71^	Recruited at McGill University Health Center
TM00089 Patient-derived xenograft (PDX)	Jackson Laboratory	https://tumor.informatics.jax.org/mtbwi/pdxDetails.do?modelID=TM00089
TM01278 Patient-derived xenograft (PDX)	Jackson Laboratory	https://tumor.informatics.jax.org/mtbwi/pdxDetails.do?modelID=TM01278
T830 TNBC Patient-derived xenograft (PDX)	In this paper	Jewish General Hospital, McGill University

**Chemicals, peptides, and recombinant proteins**		

LXG6403	Cetin et al.^28^	N/A
Leflunomide	MedChemExpress	Cat#HY-B0083
Brequinar	MedChemExpress	Cat#HY-108325
Liproxstatin-1	MedChemExpress	Cat#HY-12726
MG132	MedChemExpress	Cat#HY-13259
DMOG	MedChemExpress	Cat#HY-15893
MLN4924	MedChemExpress	Cat#HY-70062
TAK-981	MedChemExpress	Cat#HY-111789
17-AAG	MedChemExpress	Cat#HY-10211
Tetracycline	Sigma	Cat#T7660
ER-Tracker Blue-White DPX	MedChemExpress	Cat#HY-D1429
MitoTracker Red CMXRos	Fisher	Cat#M7512
Recombinant Human HSP90-alpha/HSP90AA1 Protein, HPLC-verified	Abcam	Cat#ab318253
Mature LOX rec	Origene	Cat#AR51651U-N
Parkin Recombinant, active	Sigma-Aldrich	Cat#23-048-M
ProLOX	Origene	Cat#TP313323
PINK1 Human recombinant protein	Origene	Cat#TP762427
Heat inactivated Fetal Bovine Serum	Thermo Fisher Scientific	Cat#16140071
Bovine Serum Albumin	Fisher	Cat#BP1600-1
DMSO	Sigma	Cat#317275
Trypsin	Fisher	Cat#25051Cl
DMEM	Fisher	Cat#10-014CV
100X Penicillin/Streptomycin	Fisher	Cat#15-140-122
Sodium Pyruvate	Thermo Fisher Scientific	Cat#11360070
Sulforhodamine B sodium salt	Fisher	Cat#AAA1476914
Acetic acid glacial	Spectrum	Cat#A1010
PageRuler Plus Prestained Protein ladder	Fisher	Cat#26619
Pierce BCA protein quantification	Thermo Fisher Scientific	Cat#23224
cOmplete™ ULTRA Tablets, Mini, EDTA-free, EASYpack Protease Inhibitor Cocktail	Roche	Cat#4693159001
Phosstop	Roche	Cat#4693159001
EZ-link Biotin Hydrazide	Thermo Fisher Scientific	Cat#21339

**Critical commercial assays**		

Seahorse XFe96/XF Pro FluxPak Mini	Agilent	Cat#103793-100
3D CellTiter-Glo	Promega	Cat#G9683
Lipofectamine 2000	Invitrogen	Cat#11668019
MitoSOX Red	Thermo Fisher Scientific	Cat#M36008
Image-iT Lipid Peroxidation Kit	Thermo Fisher Scientific	Cat#C10445
ROS-Glo H_2_O_2_ Assay	Promega	Cat#G8820
α-Ketoglutarate Detection Assay Kit	Cayman	Cat#701350
ENLITEN ATP Assay System	Promega	Cat#FF2000
GSSG/GSH Quantification Kit	Dojindo	Cat#50-190-3718
DuoLink *In Situ* Red Mouse/Rabbit	Sigma-Aldrich	Cat#DUO92101-1KT

**Deposited data**		

Raw RNA-seq data	This paper	Database: GSE312563 https://www.ncbi.nlm.nih.gov/geo/query/acc.cgi?acc=GSE312563
Raw Proteomics data	This paper	Database: PXD071741 https://www.ebi.ac.uk/pride/archive/projects/PXD071741
Raw Spatial Transcriptomics data	This paper	Database: GSE338674 https://www.ncbi.nlm.nih.gov/geo/query/acc.cgi?acc=GSE338674
Raw Metabolic Profiling data	This paper	Study ID: ST005017, Project doi:10.21228/M8BG4Bhttps://doi.org/10.21228/M8BG4B
RNA-seq data of breast cancer tumor samples	Sabatier R. et al.^77^	Database: GSE31448
RNA-seq data of TNBC tumors	Burstein MD. et al.^78^	Database: GSE76124
KM Plotter, basal breast cancer RNA-seq	Posta M. et al.^79^	Database: KM Plotter
Expression profiling by array TNBC tumors	Jezequel et al.^76^	Database: GSE103091
METABRIC	Curtis et al.^75^	Database: METABRIC https://ega-archive.org/studies/EGAS00000000083
TCGA	TCGA Research Network	https://www.cancer.gov/tcga
Mendeley data containing western blot images	This paper	Mendeley data: https://doi.org/10.17632/44vvkgby2v.1

**Experimental models: cell lines**		

MDA-MB-231	ATCC	Cat#HTB-26, RRID:CVCL_0062
MDA-MB-436	ATCC	Cat#HTB-130, RRID:CVCL_0623
BT-549	ATCC	Cat#HTB-122, RRID:CVCL_1092
HS578-T	ATCC	Cat#HTB-126, RRID:CVCL_0332
HEK293T	ATCC	Cat#CRL-3216, RRID:CVCL_0063
HCC1143	ATCC	Cat#CRL-2321, RRID:CVCL_1245
MDA-MB-468	ATCC	Cat#HTB-132, RRID:CVCL_0419
HCC70	ATCC	Cat#CRL-2315, RRID:CVCL_1270
HCC1806	ATCC	Cat#CRL-2335, RRID:CVCL_1258
4T1	ATCC	Cat#CRL-2539, RRID:CVCL_0125

**Experimental models: organisms/strains**		

NSG-NOD.Cg-Prkdcscid Il2rgtm1Wjl/SzJ	Jackson Labs	Cat#005557, RRID:IMSR_JAX:005557
B6.Cg-Loxem1Mech/J	Lee et al.^30^	N/A
BALB/cJ	Jackson Labs	Cat#00651, RRID:IMSR_JAX:000651

**Oligonucleotides**		

Please see Table S2 for primers	This paper	N/A
AllStar Negative Control siRNA	Qiagen	Cat#1027280
VHL siRNA sequence #1; CCGUAUGGCUCAACUUCGA	Dharmacon	Cat#D-003936-01-0002
VHL siRNA sequence #2; AGGCAGGCGUCGAAGAGUA	Dharmacon	Cat#D-003936-02-0002
LOX siRNA sequence #1; CACAGGACAUCAUGCGUAU	Dharmacon	Cat#D-009810-04-0005
LOX siRNA sequence #2; CAAUUUCACCGUAUUAGAA	Dharmacon	Cat#D-009810-17-0005
PARKIN siRNA sequence #1; GGAGUGCAGUGCCGUAUUU	Dharmacon	Cat#D-003603-01-0002
PARKIN siRNA sequence #2; UUAAAGAGCUCCAUCACUU	Dharmacon	Cat#D-003603-03-0002

**Software and algorithms**		

Seahorse Wave Desktop Software 2.6	Agilent	N/A
Graphpad Prism 10.0	Dotmatics	N/A
ImageJ (Fiji) 2.0	NIH	https://imagej.net/ij/
Image Lab 3.0	BioRad	N/A
iBright	Invitrogen	https://www.thermofisher.com
Xcalibur 4.1 software	Invitrogen	https://www.thermofisher.com
Tracefinder 4.1 software	Invitrogen	https://www.thermofisher.com
R Studio 4.3.0	R Core Team	https://www.rstudio.com/
Biorender (Agreement Number: DZ26UFS116)	Biorender	https://www.biorender.com/

### Experimental model and study participant details

#### Human tissue specimens

Primary TNBC tumor specimens were obtained from 37 female patients undergoing breast surgery at the McGill University Health Center (MUHC) between 1999 and 2012, as previously described.^80^ The mean age at surgery was, 58 ± 15 years (range, 34–90 years). Tumors were treatment-naive at surgical excision and clinically classified as lacking estrogen receptor and progesterone receptor expression and HER2 overexpression or amplification. The study was approved under MUHC Research Ethics Board protocols SDR-99-780 and SDR-00-966, and all participants provided written informed consent for the use of their data and biological specimens. Specimens were included according to tissue availability and assay-specific quality requirements, and the numbers included in individual analyses are reported in the corresponding figure legends. Gender identity, ancestry, race, ethnicity, and socioeconomic status were not available and were not included in the analyses. Since the cohort included only female participants, sex-based comparative analyses could not be performed.

#### Human datasets

De-identified transcriptomic and clinical data were obtained from The Cancer Genome Atlas (TCGA) (breast cancer patients with mean age 58, range: 26–90), METABRIC (breast cancer patients with mean age 61, range: 22–96), Gene Expression Omnibus datasets GSE103091 (TNBC patients with mean age 57, range: 28–85), GSE31448 (female breast cancer patients with invasive adenocarcinoma with 37% ≤ age of 50 and 63% > age of 50), and GSE76124 (TNBC patients with 42% < age of 50 and 58% ≥ age of 50, 96% Caucasian and 4% Asian/Pacific Islander), and the European Genome-Phenome Archive dataset EGAS00000000122, as listed in the key resources table. The single-cell RNA-seq analysis used patient-matched pre-treatment and post-treatment tumors from six patients, comprising three patients with clonal extinction and three with clonal persistence following neoadjuvant chemotherapy. No participants were recruited or prospectively assigned to groups for these secondary analyses; samples were categorized using the treatment-response annotations in the source studies. Sex-stratified analysis of the public datasets was not performed. Demographic metadata were incomplete across datasets; therefore, the influence of sex, gender, ancestry, race, and ethnicity could not be comprehensively evaluated, which limits the generalizability of these analyses.

#### Experimental animals

Female NSG mice (6–8 weeks old; Jackson lab, Strain #:005557) were used for the TNBC PDX and MDA-MB-231 xenograft studies. Female BALB/c mice (6–8 weeks old; Jackson lab, Strain #:000651) were used for the syngeneic 4T1 model. Animals were housed under standard conditions with *ad libitum* access to food and water. All animal procedures were conducted at the Medical University of South Carolina in accordance with an institutionally approved Institutional Animal Care and Use Committee (IACUC) protocol (IACUC-2022-01408). Only female mice were used because these experiments modeled TNBC of the mammary gland, a disease that predominantly affects women. Sex was therefore not included as an experimental variable, and sex-based comparisons were not performed. Whether the observed LOX-dependent metabolic and therapeutic effects differ in male animals remains unknown and limits the generalizability of the *in vivo* findings.

#### TNBC patient-derived xenograft models

Human TNBC PDX models TM01278, TM00089, and T830 were propagated in female NSG mice. TM01278 and T830 were used for *in vivo* efficacy studies, whereas TM01278 and TM00089 were used to establish PDX-derived organoids. All animal procedures were performed as described in Method Details. The TM00089 model was established from a treatment-naïve primary invasive ductal carcinoma of the breast (AJCC stage IIA, grade 3) obtained from a 68-year-old Asian or Pacific Islander, non-Hispanic female patient. The TM01278 model was established from a primary invasive ductal carcinoma of the breast (grade 3) obtained from a 35-year-old female patient who had received prior treatment before tissue collection. The patient’s race and ethnicity were not reported. T830 TNBC PDX tumor was established from a recurrent primary breast tumor of a TNBC patient previously treated with chemotherapy. The patient’s race and ethnicity were not reported.

#### Cell lines, culture conditions, and drugs

Human breast cancer cell lines, MDA-MB-231 (51-year-old Caucasian female patient with a metastatic mammary adenocarcinoma, derived specifically from a pleural effusion), BT549 (72-year-old Caucasian female patient diagnosed with an invasive breast cancer), Hs578T (74-year-old Caucasian female. The primary patient tumor was classified as a ductal carcinosarcoma (breast carcinoma) of the mammary gland), HCC1143 (52-year-old White female patient diagnosed with a primary, invasive ductal carcinoma of the breast), MDA-MB-468 (pleural effusion of a 51-year-old Black female patient with metastatic adenocarcinoma of the breast), MDA-MB-436 (43-year-old White female patient diagnosed with TNBC adenocarcinoma), HCC70 (49-year-old Black female patient with triple-negative breast cancer (TNBC). The tumor source is a poorly differentiated, grade 3 primary invasive ductal carcinoma), and HCC1806 (60-year-old Black female patient. Key clinical details include an acantholytic squamous cell carcinoma diagnosis, Stage IIB classification, and grade 2 tumor differentiation), as well as the mouse mammary tumor cell line 4T1 and the human embryonic kidney-derived HEK293T cell line were purchased from ATCC. Cells were cultured in DMEM with 10% FBS, 50 U/mL penicillin/streptomycin, 1% nonessential amino acids (Gibco, NY, USA). Cells were routinely tested for mycoplasma contamination using MycoAlert detection kit (Lonza) and were authenticated by STR sequencing.

LXG6403 was synthesized in-house as described in.^28^ Brequinar, leflunomide, liproxstatin-1, MG132, CoCl_2_, DMOG, MLN4924, TAK-981 and 17-AAG, were purchased from MedChemExpress (NJ, USA). DMOG was purchased from Tocris (UK). Ferrostatin-1 and mdivi-1 were purchased from Cayman (MI, USA). Cycloheximide, oligomycin, tetracycline, α-Ketoglutaric acid, and 2-DG were purchased from Sigma (MO, USA). Glucose was purchased from Gibco (NY, USA). MDA-MB-231 and Hs 578T derivatives expressing tetracycline-inducible shRNA targeting LOX were generated by lentiviral transduction as described in method details. The breast cancer cell lines and 4T1 were derived from female donors or female BALB/c mice. Cell-based experiments were not designed to compare donor sex, and the absence of a male-derived breast cancer model limits assessment of sex-associated effects *in vitro*.

#### Mouse embryonic fibroblasts

Mouse embryonic fibroblasts (MEFs). Wild-type and Lox knockout (Lox^ko/ko^) mouse embryonic fibroblasts (MEFs) were generously provided by Dr. Robert P. Mecham and were derived from the Lox knockout mouse model originally described in^30^ (B6.Cg-Loxem1Mech/J). Embryo sex was not determined. MEFs were cultured in high-glucose DMEM supplemented with 10% fetal bovine serum, 100 U/mL penicillin, 100 μg/mL streptomycin, and 2 mM L-glutamine. Cells were maintained under standard humidified conditions (37°C, 5% CO_2_). The Lox genotype was confirmed by PCR genotyping as previously described for the original mouse model.^30^

### Method details

#### Experimental design, replication and randomization

The numbers of independent biological/technical replicates, animals, human specimens, fields, regions of interest, and technical replicates are reported in the corresponding figure legends. Unless otherwise indicated, all *in vitro* experiments were independently repeated at least twice. Mice were enrolled after tumors reached the predetermined starting volume and were randomly assigned to treatment groups. Image acquisition settings and analysis thresholds were held constant across conditions within each experiment.

#### TNBC PDX models

2-3 mm^3^ of PDX tumor pieces from the TNBC PDX model TM01278 and T830 were transplanted to the flank or mammary fat pad region of 6–8 weeks old female NSG mice. When the tumors reached 50 mm^3^, mice were randomly distributed to 2 groups and treated with vehicle or LXG6403 for 5 days to prime the tumors. Vehicle- and LXG6403-pretreated mice were then re-randomized into treatment groups. For the TM01278 model, mice were treated with leflunomide (10 mg/kg, QD), LXG6403 (75 mg/kg, BID), or the combination. For the T830 model, mice were treated with leflunomide (10 mg/kg, QD), LXG6403 (75 mg/kg, BID), doxorubicin (2 mg/kg, weekly, i.v.), or the indicated combinations. All treatments were administered by oral gavage except doxorubicin, which was administered intravenously, for 25 days. Tumor volumes were measured twice weekly using digital calipers and calculated using the formula: volume = (length × width^2^)/2. Body weights were recorded twice weekly to monitor toxicity. At the experimental endpoint, mice were euthanized, and tumors were harvested, weighed, and processed for downstream analysis.

#### 4T1 syngeneic TNBC model

4T1 murine TNBC (1 × 10^5^) cells were orthotopically implanted into the mammary fat pad of 6–8 weeks old female BALB/c mice. When tumors reached approximately 50mm^3^, mice were randomly distributed to 2 groups and treated with vehicle or LXG6403 (75 mg/kg, BID) for 5 days to prime the tumors. Vehicle- and LXG6403-pretreated mice were then re-randomized into treatment groups and treated with leflunomide (10 mg/kg, QD), LXG6403 (75 mg/kg, BID), or the combination. All treatments were administered by oral gavage for 25 days. Tumor volumes were measured twice weekly using digital calipers and calculated using the formula: volume = (length × width^2^)/2. Body weights were recorded twice weekly to monitor toxicity. At the experimental endpoint, mice were euthanized, and tumors were harvested, weighed, and processed for downstream analysis.

#### Tetracycline-inducible shLOX xenograft model

5 × 10^6^ cells of MDA-MB-231 cells expressing tetracycline-inducible shRNA targeting LOX (shLOX) were implanted into the mammary fat pad region of 6–8 weeks old female NSG mice. Upon tumor establishment, mice were randomized into experimental groups, and tetracycline was administered via drinking water to induce LOX knockdown. After 5 days of tetracycline induction, mice were further randomized into two groups and treated with vehicle or leflunomide (50 mg/kg, QD). All treatments were administered by oral gavage for 21 days. Tumor volumes were measured twice weekly using digital calipers and calculated using the formula: volume = (length × width^2^)/2. Body weights were recorded twice weekly to monitor toxicity. At the experimental endpoint, mice were euthanized, and tumors were harvested, weighed, and processed for downstream analysis.

#### PDX-derived TNBC organoids

TNBC PDX organoids were established as previously described^26^ from freshly frozen surgical tissue by cutting the TM01278 or TM00089 PDX tumors into small pieces and incubating in collagenase A solution with ROCK inhibitor on a shaker at 37°C for 30 min. The collagenase activity was inhibited by adding FBS, and pipetting was done to ensure the formation of almost a single-cell solution. After several washes with PBS, the cell pellet was dissolved in matrigel. Breast organoid media containing ROCK and GSK inhibitors was added after the matrigel solidified. For drug testing studies, organoids were disrupted into single cells by digesting at 37°C for 30 min with TrypLE (Gibco, NY, USA) in the presence of 10 μM Rock inhibitor (Selleckchem, TX, USA). Organoids were then plated into wells of 96-well plates on a Matrigel: collagen I (1:1) coated surface with media containing 2% matrigel (Corning, NY, USA). Drugs were added 72 h after plating. Organoids were grown in the presence of drugs for 5 days. Organoid viability was measured by using the 3D Cell Titer Glo (Promega, WI, USA, Cat#G9683).

#### Proximity ligation assay

For proximity ligation assays (PLA), cells were seeded on coverslips and subjected to the indicated genetic manipulations (e.g., LOX or HSP90 overexpression, or co-expression) and/or pharmacologic treatments (e.g., LXG6403). Cells were fixed with 4% paraformaldehyde for 15 min, and PLA was performed using the DuoLink PLA kit (Sigma, MO, USA, Cat#DUO92101-1KT) according to the manufacturer’s instructions, as previously described.^81^ Antibody pairs targeting the indicated protein interactions (e.g., PARKIN-LOX, LOX-HSP90, VDAC1-IP3R) were used, and PLA signals were visualized and quantified.

For PLA localization studies of internalized LOX, cells were incubated with conditioned medium containing C-terminal His-V5-tagged proLOX for 4 h. After incubation, cells were washed with cold PBS and briefly acid-washed to remove surface-bound/non-internalized LOX. Cells were then fixed, permeabilized, and subjected to PLA using antibody pairs against V5/His and the indicated interacting proteins (PINK1 and PARKIN). Following PLA amplification, cells were immunostained with antibodies against EEA1, LAMP1, TOM20, or α-tubulin to assess localization of PLA signals relative to endosomal, lysosomal, mitochondrial, or cytoskeletal compartments, respectively. Images were acquired by confocal microscopy under identical settings across conditions, and colocalization between PLA puncta and compartment markers was quantified using the JACoP plugin in ImageJ/Fiji.^82^

#### LOX internalization assay and endocytosis inhibition

Cells were seeded on coverslips or culture plates and allowed to attach overnight. Prior to LOX treatment, cells were pre-treated with Pitstop, Dynasore, cytochalasin D or EIPA for 45 min at 37°C. Cells were then incubated with recombinant mature LOX and proLOX or conditioned medium containing His-V5-tagged mature LOX or proLOX in the continued presence of inhibitors for 4 h. Following incubation, cells were washed with cold PBS and briefly acid-washed to remove surface-bound/non-internalized LOX. Cells were then washed again with cold PBS and processed with IF staining.

#### DHODH activity

To measure DHODH activity, BT549 cells were treated with LXG6403 for 24 h and lysed in phosphatase buffer containing 0.1% Triton X-100. Similarly, TNBC PDX tumors or inducible shLOX-expressing MDA-MB-231 xenograft tumors were harvested and homogenized in phosphatase buffer containing 0.1% Triton X-100. Lysates were incubated with the mixture of D-Hydroorotic acid (DHO), decylubiquione, and 2,6-Dichloroindophenol sodium salt hydrate (DCPIP) in phosphatase buffer for 30 min at 37°C, followed by measurement of absorbance at 600 nm.

#### Stable transfections using lentiviral vectors

Lentivirus was produced in HEK293T cells by co-transfecting the transfer vector with psPAX2 and pMD2.G at a 4:3:2 mass ratio using Lipofectamine. Supernatants were collected at 48 and 72 h, filtered (0.45 μm), and used fresh or stored at −80°C. Target cells were transduced with viral particles in complete medium containing polybrene (10 μg/mL) for 48 h then replaced with fresh medium. Stable populations were selected for 3–7 days with puromycin (1–2 μg/mL) and maintained at half the selection dose thereafter. Expression/knockdown was verified by immunoblotting and/or qPCR.

#### Seahorse assay for determining ECAR and OCR

TNBC cell lines (MDA-MB-231) and mouse embryonic fibroblasts (MEFs; wildtype and LOX^ko/ko^) were seeded at 2 × 10^4^ cells/well in XF96 cell culture microplates (Agilent) in RPMI-1640 medium with 10% FBS and cultured overnight at 37°C with 5% CO_2_. TNBC cells were treated with LXG6403 (15 μM) or vehicle (DMSO, <0.1% v/v) for 24 h. MEFs were assayed without treatment to compare LOX^wt/wt^ vs. LOX^ko/ko^. Prior to the assay, the medium was replaced with XF base medium (Agilent) supplemented with 10 mM glucose, 1 mM pyruvate, and 2 mM glutamine (pH 7.4), and cells were incubated for 1 h at 37°C in a non-CO_2_ incubator. ECAR and OCR were measured using the Seahorse XF96 Analyzer (Agilent) with the XF Cell Mito Stress Test and Glycolysis Stress Test kits. For OCR, sequential injections of oligomycin (1 μM), FCCP (1 μM), and rotenone/antimycin A (0.5 μM) assessed basal respiration, ATP production, maximal respiration, and non-mitochondrial respiration. For ECAR, injections of glucose (10 mM), oligomycin (1 μM), and 2-deoxyglucose (50 mM) evaluated glycolysis, glycolytic capacity, and non-glycolytic acidification. Data were normalized to cell numbers using a CyQUANT assay (Thermo Fisher, MA, USA) and analyzed with Wave software (Agilent, CA, USA). Experiments were performed in 5–17 replicates, and statistical significance was determined using one-way ANOVA with Dunnett’s post-hoc test (*p* < 0.05) in GraphPad Prism.

#### Metabolic profiling

MDA-MB-231 cells were seeded at 1 × 10^6^ cells per 60-mm dish in RPMI-1640 medium supplemented with 10% FBS and cultured at 37°C with 5% CO_2_. After 24 h, cells were treated with LXG6403 (15 μM or vehicle (DMSO, <0.1% v/v) for 24 h. Cells were washed with ice-cold PBS, quenched with 80% methanol at −80°C, and scraped into 1 mL of extraction solvent (80:20 methanol:water with internal standards, e.g., 13C-labeled glutamine). Metabolites were extracted by three freeze-thaw cycles, centrifuged at 14,000 × g for 10 min at 4°C, and the supernatant was dried under nitrogen. Samples were analyzed by High-Performance Liquid Chromatography and High-Resolution Mass Spectrometry and Tandem Mass Spectrometry (HPLC-MS/MS). Specifically, system consisted of a Thermo Q-Exactive in line with an electrospray source and an Ultimate3000 (Thermo) series HPLC consisting of a binary pump, degasser, and auto-sampler outfitted with a Xbridge Amide column (Waters; dimensions of 3.0 mm × 100 mm and a 3.5 μm particle size). The mobile phase A contained 95% (vol/vol) water, 5% (vol/vol) acetonitrile, 10 mM ammonium hydroxide, 10 mM ammonium acetate, pH = 9.0; B was 100% Acetonitrile. The gradient was as following: 0 min, 15% A; 2.5 min, 64% A; 12.4 min, 40% A; 12.5 min, 30% A; 12.5–14 min, 30% A; 14–21 min, 15% A with a flow rate of 150 μL/min. The capillary of the ESI source was set to 275°C, with sheath gas at 35 arbitrary units, auxiliary gas at 5 arbitrary units and the spray voltage at 4.0 kV. In positive/negative polarity switching mode, an m/z scan range from 60 to 900 was chosen and MS1 data was collected at a resolution of 70,000. The automatic gain control (AGC) target was set at 1 × 10^6^ and the maximum injection time was 200 ms. The targeted ions were subsequently fragmented, using the higher energy collisional dissociation (HCD) cell set to 30% normalized collision energy in MS2 at a resolution power of 17,500. Besides matching m/z, target metabolites are identified by matching either retention time with analytical standards and/or MS2 fragmentation pattern. Data acquisition and analysis were carried out by Xcalibur 4.1 software and Tracefinder 4.1 software, respectively (both from Thermo Fisher Scientific). Significance was determined using Student’s *t* test (*p* < 0.1).

#### Whole-transcriptome sequencing (RNA-seq) and data analysis

RNA-seq of total RNAs isolated from TNBC cells (MDA-MB-231, Hs578-T and MDA-MB-436) treated with LXG6403 for 24 h were performed in duplicates using an Illumina NovaSeq 6000 platform at McGill University Genome Center as described previously.^83^ Library preparation was performed using NEB rRNA-depleted (HMR) stranded library preparation kit according to manufacturer’s instructions. Raw paired reads were used to quantify transcript abundances using Kallisto v0.46.1 (parameters: –-rf-stranded).^84^ Transcript information was obtained from Ensembl (release 111). Transcript-level abundance estimates were collapsed to gene-level counts using the ‘‘tximport’’ and count matrices were created, genes with a mean smaller than 30 counts were removed.^85^ Differentially expressed genes were identify using DESeq2 (shrinkage type = apeglm), *p*-values were adjusted for multiple testing using the Independent Hypothesis Weighting procedure.^86^ Gene set enrichment analysis was performed with the R package fgsea and the Hallmark gene collection.

#### GeoMx spatial profiling

Spatially resolved transcriptome data of a TNBC PDX was generated using the GeoMx - Digital Spatial Profiler - Human Whole Transcriptome Atlas at NanoString lab as previously described.^87^ To visualize GLUT1 vs. GS-rich regions of the tumor, antibodies against CD31, GS and GLUT1 were used. Regions of interests (ROIs) were selected based on GLUT1 vs. GS positivity in four regions for each group. Data acquisition and analysis were performed following NanoString guidelines as previously described.^87^

#### scRNA-seq data analysis

Single-cell RNA-seq data was obtained from,^54^ corresponding to patient-matched pre-treatment and post-treatment tumors from three patients that showed clonal extinction of tumor cells after neoadjuvant chemotherapy and three patients that showed clonal persistence. Counts were converted to transcript-per-million (TPM) for each cell. LOX+ cells were defined as those showing expressing LOX gene at levels; 10 TPM. A multivariate logistic regression was used to examine the relationship between the proportion of LOX+ cells and tumor characteristics, in the form of: where t is the treatment status of the tumor (0: pre-treatment; 1: post-treatment), r is the chemoresistance status (0: clonal extinction; 1: clonal persistence), and n is a covariate accounting for gene coverage (i.e., overall number of detected genes per cell). The regression revealed no difference between pre-treatment extinction and pre-treatment persistence groups (*p* = 0.26) but suggests a significant increase in the LOX+ cells in the persistence group after treatment (*p* < 10^−15^). To calculate gene-gene correlations, TPM values were converted to log-scale after adding a pseudocount of 0.01. Pairwise Pearson correlations were calculated for each sample separately. For each sample, the top 500 genes with the highest correlation with LOX were used for pathway enrichment analysis, using Hallmark pathways from MSigDB.^88^ Fisher’s exact test was used to evaluate overlap between genes of each pathway and the top 500 genes in each sample, followed by FDR adjustment.

#### Western blotting

Protein isolation and WB were performed as previously described.^55^^,^^83^^,^^89^^,^^90^^,^^91^ Briefly, RIPA buffer was used to isolate total protein lysate in the presence of protease and phosphatase inhibitor cocktails, and protein concentrations were measured using the BCA Protein Assay Reagent Kit (Thermo Fisher Scientific, MA, USA). For extracting proteins from collagen-embedded cells, cells were treated with 1.5 mg/mL of collagenase solution for 5 min at 37°C. Equal amounts of protein were separated using 8–10% SDS-PAGE gel. Separated proteins were transferred to PVDF membranes (Bio-Rad, CA, USA) using a Trans-Blot turbo transfer system (Bio-Rad, CA, USA) and incubated with primary antibodies listed in key resources table at the indicated dilutions (Table S1). Horseradish peroxidase-conjugated anti-mouse or anti-rabbit antibodies (Cell Signaling Technology, MA, USA) were used as secondary antibodies (key resources table, Table S1), and signals were detected by enhanced chemiluminescence (Thermo Fisher Scientific, MA, USA). Images were acquired using Image Lab Software (Bio-Rad, CA, USA) or iBright Software (Invitrogen).

#### Quantitative RT-PCR analysis

Total RNA was extracted from cultured cells using Quick-RNA Miniprep Kit (Zymo) or from polysomes using Trizol LS (ThermoScientific) and cDNAs were generated using RevertAid RT Reverse Transcription Kit (Life Technologies). qRT-PCR analysis was performed with gene-specific primers listed in Table S2 using LightCycler 480 SYBR Green I Master kit (Roche). *HPRT1* and *ACTB* were used as housekeeping genes. The average Ct value was calculated from triplicates of each sample, and the relative mRNA expression was determined.

#### Transient transfection with siRNAs and overexpression reporters

Transfections were carried out by seeding cells in media containing no antibiotics, and changing media with growth factor-reduced Optimem (Gibco, MA, USA) prior to transfection with Lipofectamine 2000 (Invitrogen, CA, USA) in 6-well or 96-well format. siRNA transfections (key resources table) were performed at 80 nM concentrations, and RNA or protein was isolated after 24 h of transfection. Transfection with overexpression vectors listed in key resources table, was performed at 500 ng in 6-well format. Hypoxia induction was performed using hypoxia chamber with 1% oxygen and 5% carbon dioxide or using 200 μM of CoCl_2_ or DMOG with overnight incubation at 37°C.

#### Co-immunoprecipitation

To analyze the binding of PARKIN with LOX, PINK1, and HIF-1α, MDA-MB-231 TNBC cells were treated with LXG6403 for 6 h. To analyze the binding of different forms of LOX, HEK293T cells were transfected with proLOX, mature LOX, and BMP1 cleavage-resistant LOX mutant vectors. V5-tagged proLOX and mature LOX were cloned from 4T1 cells while BMP1 cleavage-resistant LOX mutant vector was generated from proLOX by site-directed mutagenesis. Cells were lyzed in lysis buffer (50 mM TrisHCl pH 7.0, 150 mM NaCl, 0.2% NP40, 7.5% glycerol, protease and phosphatase inhibitor cocktail) and clarified by centrifugation. 1 mg protein for each condition was incubated with Dynabeads Protein G (Invitrogen) coated with PARKIN (Proteintech, Cat#14060-I-AP, RRID: AB_2882028) or V5 (Invitrogen, Cat#R960-25, RRID: AB_2556564) antibodies at the indicated dilutions (Table S1) overnight at +4°C. Beads were washed and resuspended in 60 μL 1X SDS sample loading buffer, boiled for 10 min at 70°C and loaded onto a polyacrylamide gel. WB was done using antibodies provided in key resources table at the indicated dilutions (Table S1).

#### Recombinant protein pulldown assay

Recombinant LOX (Origene, Cat#TP313323), PINK1 (Origene, Cat#TP762427), PARKIN (Sigma-Aldrich, Cat#23-048-M), and HSP90 (Abcam, Cat#ab318253) proteins were incubated in binding buffer (50 mM Tris-HCl pH 7.4, 150 mM NaCl, 0.2% NP-40) at 4°C for 2–4 h. Complexes were captured using tag-specific or antibody-conjugated beads, washed extensively, and analyzed by SDS-PAGE followed by immunoblotting.

#### Lysyl oxidase activity

Lysyl oxidase activity within cells or within tumors was measured using fluorometric Lysyl Oxidase Activity Assay Kit (Abcam, MA, USA) according to manufacturer’s instructions. Briefly, serially diluted replicates of tumor lysates or cell supernatants were incubated with reagent mix and subsequently measured in fluorescence microplate reader at Ex/Em = 540/590 nm wavelength. Tumor lysates were prepared by sonicating tissue samples in extraction buffer (6 M urea, 10 mM Tris pH 7.4 and protease inhibitors) followed by centrifugation.

#### *In vitro* oxidation assay

Recombinant PINK1 protein (Origene, Cat#TP762427) and bead-bound VGLL3 (positive control) were incubated with recombinant LOX (Origene, Cat#TP313323) in oxidation buffer (50 mM HEPES, 10 mM CaCl_2_, pH 8.0) at 37°C for 1 h in the presence or absence of LXG6403. Following incubation, protein carbonyl groups generated by oxidation were derivatized using biotin hydrazide (Thermo Fisher Scientific, Cat#21339) for 1 h at room temperature, as previously described.^92^ Excess reagent was removed, and biotin-labeled proteins were captured using streptavidin-conjugated beads. Bound proteins were eluted and analyzed by immunoblotting.

#### Immunofluorescence staining

Cell fixation was performed with 4% paraformaldehyde for 15 min, permeabilized and blocked in 3% BSA-PBS. Cells were incubated in the primary antibodies listed in key resources table at the indicated dilutions (Table S1), overnight and secondary Alexa Fluor 647 or 488-labeled antibodies (Table S1) for 2 h at 37°C. Cells were also counterstained with DAPI for 5 min. Images were acquired using ZEN Black LSM 880 airyscan (Carl Zeiss, DE) or Leica Stellaris 8 TauSTED Xtend. Images were analyzed using the ImageJ (Fiji) software.

Human tumors from^80^ were first heated in 60°C oven for 20 min. Deparaffinization was done in Xylene for 15 min two times, followed by washing two times in 100% and once in 70% ethanol, sequentially. Antigen retrieval was done in Tris-EDTA (pH 9.0) buffer for 1 h, and slides were incubated with antibodies listed in key resources table at the indicated dilutions (Table S1) in 5% BSA-PBS for overnight at +4°C, followed by 2 h incubation with secondary antibodies at 1:1000 dilution at 37°C. DAPI solution was prepared in 5% BSA-PBS at a dilution 1:1000 was used for nuclear counterstaining for 5 min at room temperature. Imaging was performed using Zeiss LSM880 NLO with Airscan Confocal Microscopy or Leica Stellaris 8 TauSTED Xtend, and quantification of staining intensity was done using Fiji (ImageJ) software.

#### SPLICS reporter assay

ER-mitochondria contact sites were assessed using SPLICS short and long reporters as previously described.^41^ Cells were seeded in ibidi μ-Slide chambers and transfected with SPLICS short (Addgene, Cat#164108, RRID: Addgene_164108) and long (Addgene, Cat#164107, RRID: Addgene_164107) constructs (0.5 μg DNA per well) and imaged by confocal microscopy. Fluorescent puncta corresponding to ER-mitochondria sites were quantified using ImageJ. For live-cell imaging, cells were incubated with ER-Tracker (Cat#HY-D14290 and MitoTracker (Cat#M7512) dyes according to the manufacturer’s instructions prior to imaging.

#### Calcium flux measurement

ER, mitochondrial, and cytosolic Ca^2+^ levels were measured using ER-GCaMP6-150 (Addgene, Cat#86918, RRID: Addgene_86918), Mito4x-GCaMP6f (Addgene, Cat#127870, RRID: Addgene_127870), and GCaMP6F (Addgene, Cat#163045, RRID: Addgene_163045), respectively. Cells were seeded in 96-well plates and transfected with genetically encoded Ca^2+^ sensors (250 ng DNA per well), and fluorescence intensity was measured using a plate reader in kinetic mode (Ex/Em: 488/510 nm). Time-resolved fluorescence intensity was recorded at 30 s intervals, and Ca^2+^ dynamics were quantified as changes in fluorescence over baseline.

#### Mitochondrial and cytosolic ROS measurement

Hs578T TNBC cells were seeded on black 96-well plates. Cells were transfected with WT or S65A mutant PARKIN vectors or the LOX ORF (50 ng/well) for 24 h followed by tetracycline administration to induce LOX knockdown. Next day, cells were treated with LXG6403 (30 μM) or CCCP (2 μM) for 24 h. For mitochondrial ROS measurement, the MitoSOX Red Mitochondrial Superoxide Indicator (Thermo Fisher Scientific, Cat#M36008) was utilized based on manufacturer’s instructions, as previously described.^28^ For cytosolic ROS, the ROS-Glo H2O2 Assay kit (Promega, Cat#G8820) was utilized based on the manufacturer’s instructions.

#### ATP measurement

Cellular ATP content was measured in lysed cells with the Cell Titer Glo assay (Promega, Cat#FF2000) in 96-well plates according to the manufacturer’s specifications as in.^93^^,^^94^

#### TMRE staining

For mitochondrial membrane potential quantification, TMRE staining (1:1000) was performed. Cover slips were mounted with ProLong Glass Antifade Mountant (ThermoScientific). Imaging was done using Zeiss LSM880 NLO with Airscan Confocal Microscopy or Leica Stellaris 8 TauSTED Xtend, and quantification of staining intensity was done using Fiji (ImageJ) software.

#### Lipid peroxidation-C11 staining

To assess lipid peroxidation, MDA-MB-231 cells were seeded at 2 × 10ˆ5 cells/well in 6-well plates and treated with LXG6403 (15 μM) or brequinar (50 μM) or their combination for 2 h. Lipid peroxidation was measured using the Image-iT lipid peroxidation (Thermo Fisher Scientific, Cat#C10445) assay using a plate reader. Data were background-subtracted and quantified as the mean fluorescence intensity ratio of oxidized to non-oxidized signal, normalized to untreated controls.

#### BioID protein-proximity labeling

Cells were seeded at 40–50% confluency and transfected the next day with HA-tagged LOX–BioID2 (LOX-ID) or HA–BioID2 control plasmids using Lipofectamine (Thermo Fisher Scientific). At 24 h post-transfection, culture media were supplemented with D-biotin (50 μM; Thermo Fisher Scientific) and cells were incubated for 18 h under hypoxia (e.g., 1% O_2_). Cells were washed with ice-cold PBS and lysed on ice in stringent lysis buffer (50 mM Tris-HCl pH 7.4, 500 mM NaCl, 0.4% SDS, 1% Triton X-100, 1 mM EDTA, 1 mM DTT) containing protease inhibitor cocktail (Roche) and phosphatase inhibitors (Roche). Lysates were briefly sonicated on ice and clarified at 16,000 × g for 15 min at 4°C; supernatants were incubated with pre-washed streptavidin magnetic beads (Thermo Fisher Scientific) overnight at 4°C. Beads were then subjected to six sequential high-stringency washes (5 min each, rotation): (1) 2% SDS in water; (2) lysis buffer; (3) 50 mM Tris-HCl pH 7.4, 500 mM NaCl, 1% Triton X-100, 0.1% sodium deoxycholate; (4) 10 mM Tris-HCl pH 7.4, 250 mM LiCl, 0.5% NP-40, 0.5% sodium deoxycholate; (5) 50 mM Tris-HCl pH 7.4, 50 mM NaCl; and (6) 50 mM Tris-HCl pH 7.4. For mass spectrometry analysis, the beads were subjected to on-bead trypsin digestion.

#### Mass spectrometry

Proteins were digested directly off the streptavidin magnetic beads. Beads were washed twice with 50 mM ammonium bicarbonate and the enriched proteins were reduced with 4.1 mM dithiothreitol and alkylated with 8.3 mM iodoacetamide. Trypsin/LysC (200 ng) was added to each sample and incubated overnight at 37°C with shaking at 300 rpm. The digestion was terminated with the addition of formic acid to final concentration of 1%. The supernatant containing the tryptic peptides was desalted with C18 Stage Tips and dried under vacuum.

Peptides were separated and analyzed on an EASY nLC 1200 System in-line with the Orbitrap Exploris 480 Mass Spectrometer (Thermo Scientific). Two μg of peptides were pressure loaded onto a C18 reversed phase column (Acclaim PepMap RSLC, 75 μm × 25 cm (2 μm, 100 Å) Thermo Scientific). The peptides were separated using a gradient of 0–35% B in 180 min (Solvent A: 5% acetonitrile, 0.1% formic acid; Solvent B: 80% acetonitrile, 0.1% formic acid) at a flow rate of 300 nL/min. Spray voltage was set at 2.2 kV and ion transfer tube temperature at 300°C. Mass spectra were acquired in data-dependent mode with a high resolution (60,000) full scan, mass range of m/z 375–1575, with an automatic gain control target value of 300% and a maximum injection time of 25 ms. Monoisotopic peak determination was enabled. The AGC target value for fragment spectra is set at 100%. Cycle time for MS2 scans was 3 s. The resolution was set at 15,000 with a max injection time of 40 ms. An HCD collision energy of 33% was used for peptide fragmentation. Dynamic exclusion of precursors was set to 20 s.

The raw files were searched in Max Quant v2.4.2.0 (Max Planck Institute) against the reviewed human protein database. The search parameters allowed for two missed cleavages, fragment mass tolerances of 20 ppm, and a minimum of seven amino acids per peptide. Carbamidomethylation of cysteine was set as a fixed modification. Methionine oxidation and acetylation of protein N-termini were set as variable modifications. The false discovery rates for peptide spectral matches and protein identification were set to 1% using a reversed database decoy strategy. The search results were further analyzed and filtered in Perseus v. 1.6.15.0 (Max Planck Institute). Reversed database matches and potential contaminants including streptavidin were removed. Only proteins with >2 peptides including a unique peptide were retained. The LFQ and iBAQ protein intensities were log2 transformed. To identify proteins with changes in relative abundance between conditions, a log2 intensity cut-off of 1.5 was selected.

#### Bioinformatics analysis

Publicly available patient data including The Cancer Genome Atlas (TCGA) (https://www.cancer.gov/tcga), as well as Gene Expression Omnibus (GEO) databases listed in key resources table (GSE103091,^76^ GSE31448,^77^ GSE76124^78^) were analyzed. Survival analyses were performed using the KM Plotter database^79^ and METABRIC.^75^ Transcriptomic data retrieved from the NCBI GEO database and Molecular Taxonomy of Breast Cancer International Consortium project data from EMBL European Genome-Phenome Archive^75^ (http://www.ebi.ac.uk/ega/) with an accession number EGAS00000000122. Pathway enrichment analysis was performed using STRING database.^95^ The network analysis of the metabolic profiling data was performed using the Pathway enrichment function in MetaboAnalyst^96^ (https://www.metaboanalyst.ca/).

### Quantification and statistical analysis

All the results are represented as mean ± standard deviation (SD) or mean ± standard error of the mean (SEM), as indicated in the figure legends. Differences were assessed using the two-tailed unpaired or paired Student’s *t* test. For comparisons involving more than two groups, one-way analysis of variance (ANOVA) followed by Dunnett’s post-hoc test was used, as indicated in the figure legends. *In vivo* tumor volume data was analyzed using longitudinal linear mixed-effects (LME) models, with the natural logarithm of tumor volume as the outcome variable. Predictor variables included the natural logarithm of baseline tumor volume, time (treated as a categorical variable), treatment group, and their interaction. Tumor weights at the end of the experiment were summarized by treatment group using means, standard errors, and 95% confidence intervals. Pairwise comparisons between the Combo group and each treatment group were performed using the Wilcoxon rank-sum test. *p*-values were adjusted for multiple comparisons using Holm’s method for both tumor volume and tumor weight analyses. Statistical analyses were conducted using GraphPad Prism Software and R version 4.3.0 using the packages nlme (v3.1-164), emmeans (v1.10.4), survival (v3.7–0), and survminer (v0.4.9). IF staining of cells and patient tumor samples was quantified using ImageJ software by measuring fluorescence intensity or counting positive cells per field, as specified in the corresponding figure legends. Unless otherwise indicated, all *in vitro* experiments were independently repeated at least twice. For microscopy-based analyses, multiple randomly selected fields per sample were quantified as indicated in the figure legends. The differences were considered statistically significant if *p* < 0.05 and indicated by ∗ or *p* < 0.01 and indicated by ∗∗ in the figure legends.

## Supplemental information

Document S1. Figures S1–S14 and Tables S1 and S2

Document S2. Article plus supplemental information
