# Supplementary material for: Lysyl oxidase inhibition disrupts mitochondrial homeostasis to create vulnerability to ferroptosis in TNBC
Source: Cell Rep Med. 2026 Aug 31;7(9):103015. doi: 10.1016/j.xcrm.2026.103015 (PMC13589490; doi:10.1016/j.xcrm.2026.103015)
Supplement: Supplementary file 1 [file mmc1.pdf]

Cell Reports Medicine, Volume *7*

Supplemental information

Lysyl oxidase inhibition disrupts mitochondrial homeostasis to create vulnerability to ferroptosis in TNBC

Ozge Saatci, Burge Ulukan, Metin Cetin, Hellen Kuasne, Constanza Martinez, Elif Percin, Natalia Oleinik, Matthew T. Savoca, Ali Nehme, Aldo Hernández-Corchado, Evelyn Zavacky, Kukkamudi Sreenivas, Abdol H. Rezaeian, Jennifer R. Bethard, Jean-Sebastien Anoma, Ozlem Sener Sahin, Adriana Aguilar-Mahecha, Marguerite Buchanan, Mertkaya Aras, Genevieve DeBlois, Mark Basik, Elizabeth G. Hill, Lauren E. Ball, Chintada Nageswara Rao, Campbell McInnes, Yasser Riazalhosseini, Hamed S. Najafabadi, John J. Lemasters, Besim Ogretmen, Morag Park, and Ozgur Sahin

SUPPLEMENTAL FIGURES

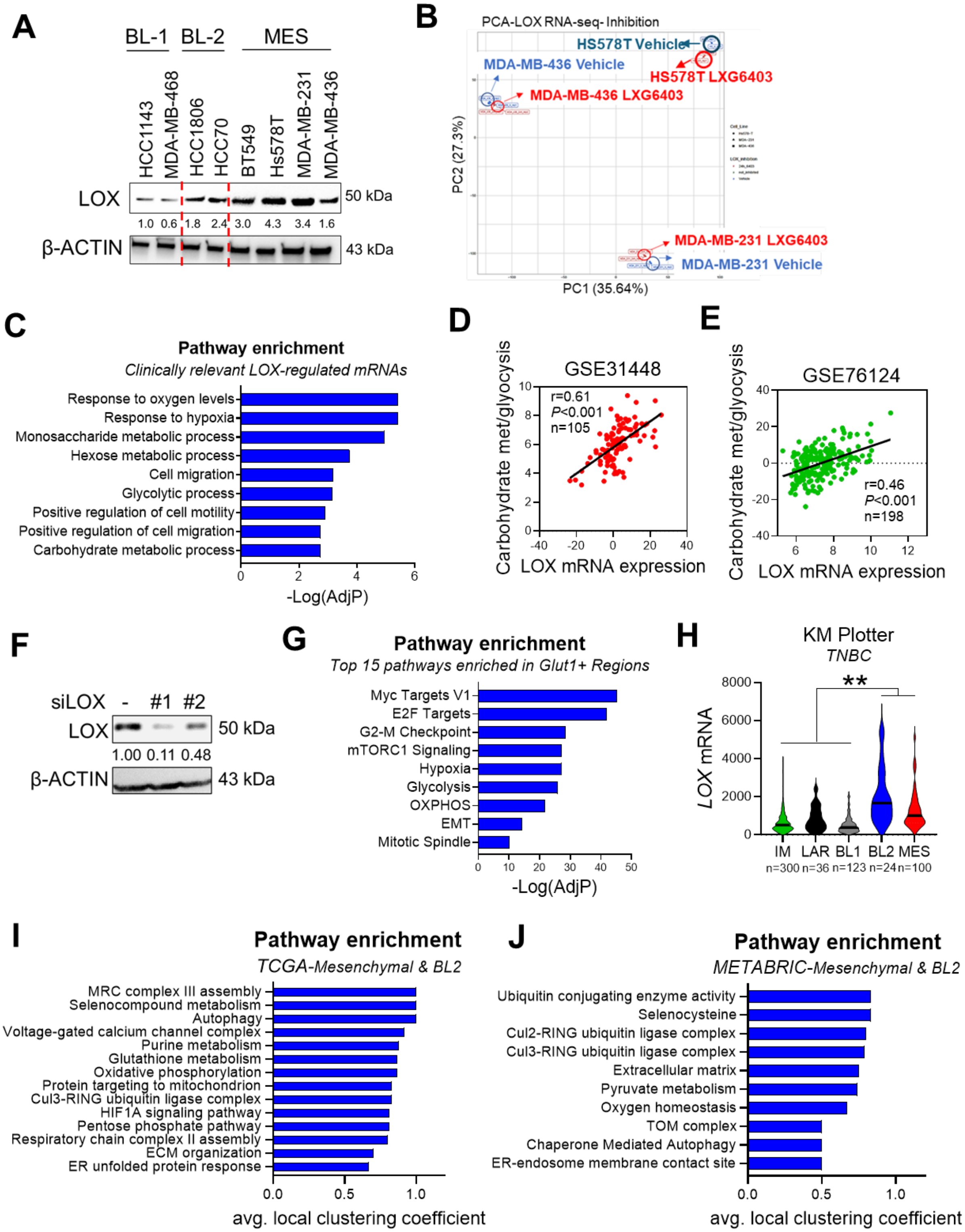

Figure S1. LOX expression, LOX-correlated gene signatures, and pathway enrichment analyses in TNBC cells and genes enriched in GLUT1+ regions in GeoMx spatial profiling of TNBC PDX tumors. Related to Figure 1. (A) WB of LOX in TNBC cell lines belonging to different subtypes. β-ACTIN was used as the loading control here and in all WB subfigures. The relative band intensities normalized to loading control based on densitometry analysis are provided below the respective blots here and in all WB subfigures. (B) Principal component analysis (PCA) of vehicle and LXG6403-treated TNBC cells (n=2 biological replicates). (C) Pathways enriched among clinically relevant LOX-regulated genes in TNBC tumors from GSE10391. (D, E) Pearson correlation between LOX and carbohydrate/glycolysis metabolism signature scores in two different TNBC patient datasets. (GSE31448; n=105 patients, GSE76124; n=198 patients). (F) WB of LOX in siLOX-transfected BT549 cells. (G) Pathways enriched among genes highly expressed in GLUT1-rich regions identified by GeoMx spatial profiling of TNBC PDX tumors. (H) LOX mRNA expression in TNBC patients stratified into immunomodulatory (IM) (n=300 patients), luminal androgen receptor (LAR) (n=36 patients), basal-like 1 (BL1) (n=123 patients), basal-like 2 (BL2) (n=24 patients), and mesenchymal (MES) (n=100 patients) subtypes. (I, J) Clustering analysis of genes correlating with LOX in MES and BL2 patient tumors from TCGA (I) and METABRIC (J). Statistical significance was determined using Pearson correlation analysis (D, E) and *P*-value was calculated with unpaired, two-tailed Student’s t test (H). **, *P*-value < 0.01.

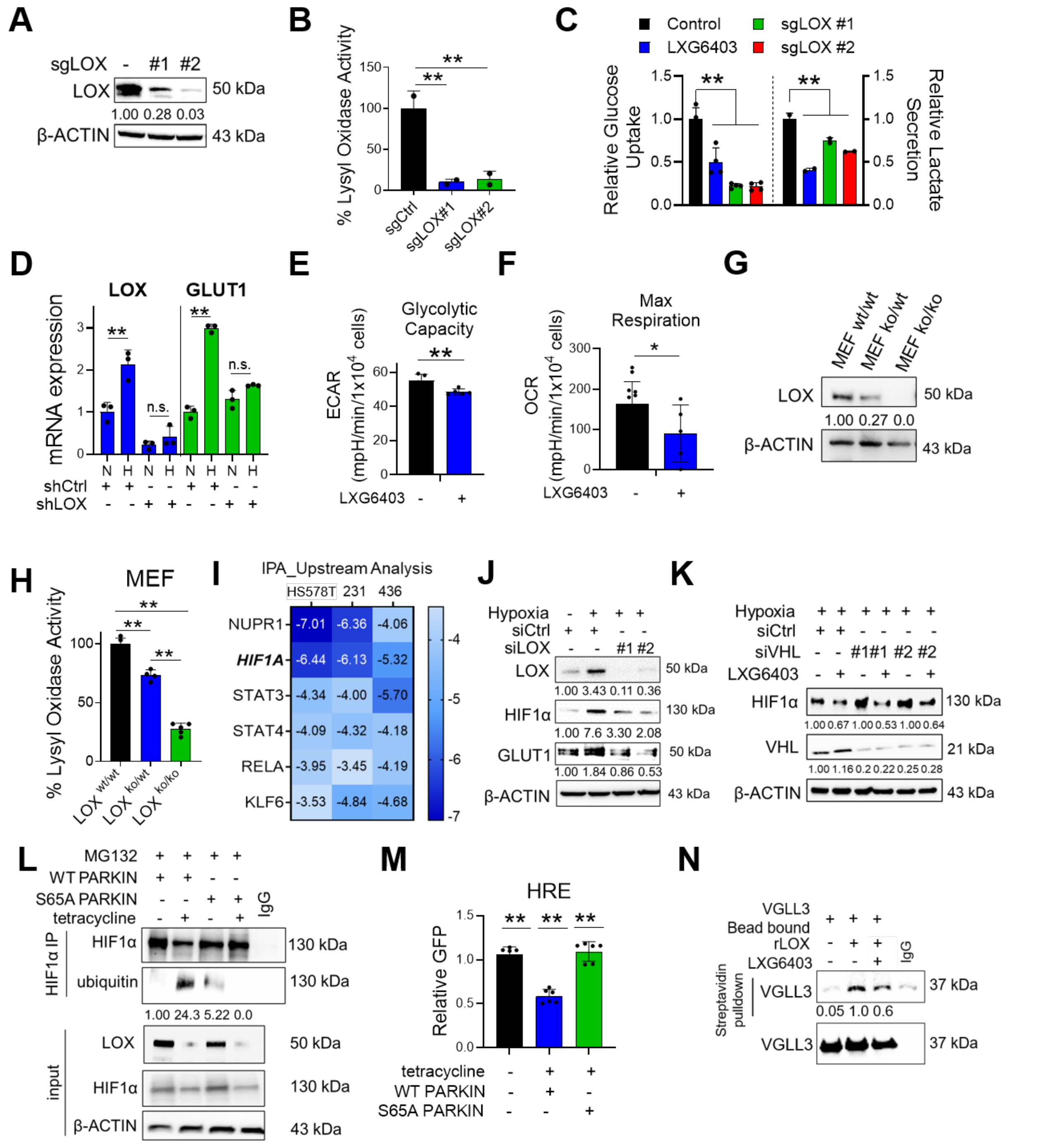

Figure S2. Validation of LOX knockdown/knockout with sgRNAs and MEFs isolated from LOX$^{wt/wt}$, LOX$^{ko/wt}$, and LOX$^{ko/ko}$ transgenic mice and the effects of LOX inhibition on glycolysis blockage via HIF-1α ubiquitination and degradation. Related to Figures 2, 3. (A, B) WB of LOX (A) and LOX enzymatic activity (B) in MDA-MB-231.sgLOX cells (n=2 technical

replicates). (C) Levels of glucose uptake and lactate secretion upon LOX inhibition by LXG6403 or sgLOX (n=4 technical replicates). (D) qRT-PCR of LOX and GLUT1 in shCtrl vs. shLOX cells in normoxia (N) vs. hypoxia (H) (n=3 biological replicates). (E, F) Seahorse analyses in MDA-MB-231 cells treated with LXG6403 (15 μM) for 24 hours, showing glycolytic capacity (ECAR) (E) and maximal respiration (OCR) (F) (n=5-12 technical replicates). (G) WB of LOX in MEFs isolated from $LOX^{wt/wt}$, $LOX^{ko/wt}$, and $LOX^{ko/ko}$ transgenic mice. (H) LOX enzymatic activity in MEF cells from (C) (n=4 technical replicates). (I) Heatmap showing the transcription factors based on the upstream analysis of genes downregulated by LOX inhibition using LXG6403 across three TNBC cell lines. (J) WB of LOX, HIF-1α, and GLUT1 in two different siLOX-transfected cells under hypoxia. (K) WB of HIF-1α and VHL in MDA-MB-231 cells transfected with siVHL and treated with LXG6403 (15μM for 18 hours) under hypoxia. (L) Ubiquitination of HIF-1α in cells expressing WT or S65A PARKIN following LOX inhibition using tetracycline-inducible shLOX. MG132 (10 uM) was utilized to inhibit proteasomal degradation. (M) HRE reporter activity in cells expressing WT or S65A PARKIN following tetracycline-inducible LOX knockdown (n=6 technical replicates). (N) WB of VGLL3 oxidation using bead-bound VGLL3 and recombinant LOX proteins. Oxidized VGLL3 is enriched by streptavidin pulldown, and this signal is reduced upon LOX inhibition. Data are presented as mean ± SD. *P*-values for the bar plots graph were calculated with the unpaired, two-tailed Student's t test (B, C, D, E, F, H and M) *, *P*-value < 0.05; **, *P*-value < 0.01.

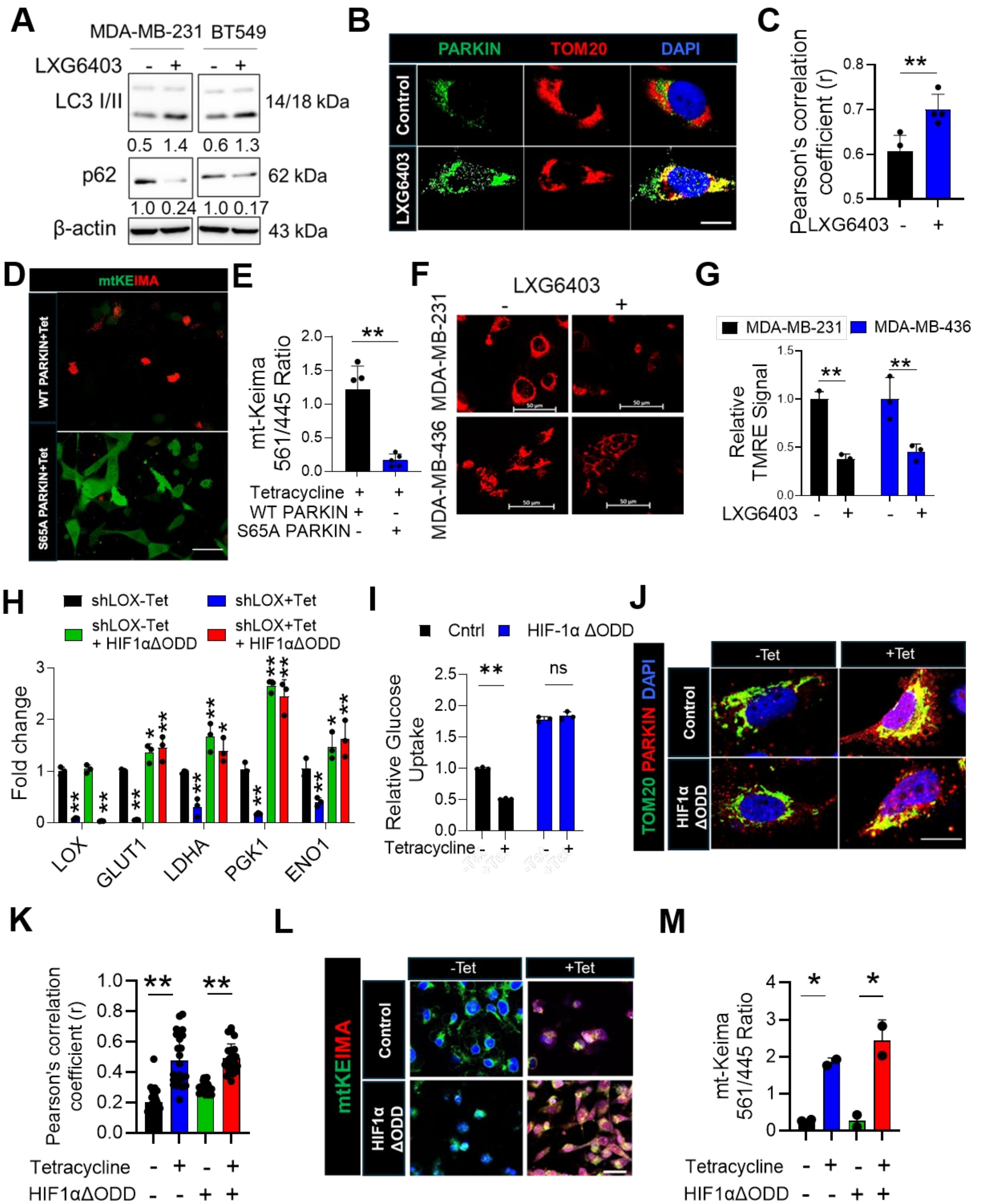

Figure S3. LOX inhibition disrupts mitochondrial dynamics and induces PINK1-dependent PARKIN activation to drive mitophagy independently of HIF-1α-mediated glycolysis.

Related to Figures 3, 4. (A) WB of LC3 I/II and p62 upon LXG6403 treatment (15μM for 18 hours) of MDA-MB-231 and BT549 cells. (B) IF staining of PARKIN, TOM20, and DAPI in MDA-MB-231 cells treated with LXG6403 (15 μM) for 1 hour. Scale bar: 10 μm. (C) Quantification of the co-localization between PARKIN and TOM20 from (B) (n=4 images). (D, E) Assessment of mitophagy using mitoKeima reporter assays (D) and its quantification (E) (n=5 images). Scale bar: 50 μm. (F, G) Representative TMRE images (F) and its quantification (G) in MDA-MB-231 and MDA-MB-436 cell lines upon LXG6403 treatment (15μM for 1 hour) (n=3 images). Scale bar: 50 μm. (H) qRT-PCR of *LOX*, *HIF-1A* and targets of *HIF-1A* involved in glycolysis in tetracycline-inducible shLOX-expressing MDA-MB-231 cells upon HIF1αΔODD overexpression. (n=3 technical replicates). (I) Rates of glucose uptake in tetracycline-inducible shLOX-expressing MDA-MB-231 cells upon HIF1αΔODD overexpression. (n=3 technical replicates). (J, K) Co-localization of PARKIN and TOM20 (J) and its quantification (K) (n=25 cells). Scale bar: 10 μm. (L, M) Mitophagy analysis using mitoKeima reporter assays (L) and quantification (M) (n=2 images). Scale bar: 10 μm. Data are presented as mean ± SD. *P*-values for the bar plots graph were calculated with the unpaired, two-tailed Student's t test (C, E, G, H, I, K and M) *, P-value < 0.05; **, P-value < 0.01.

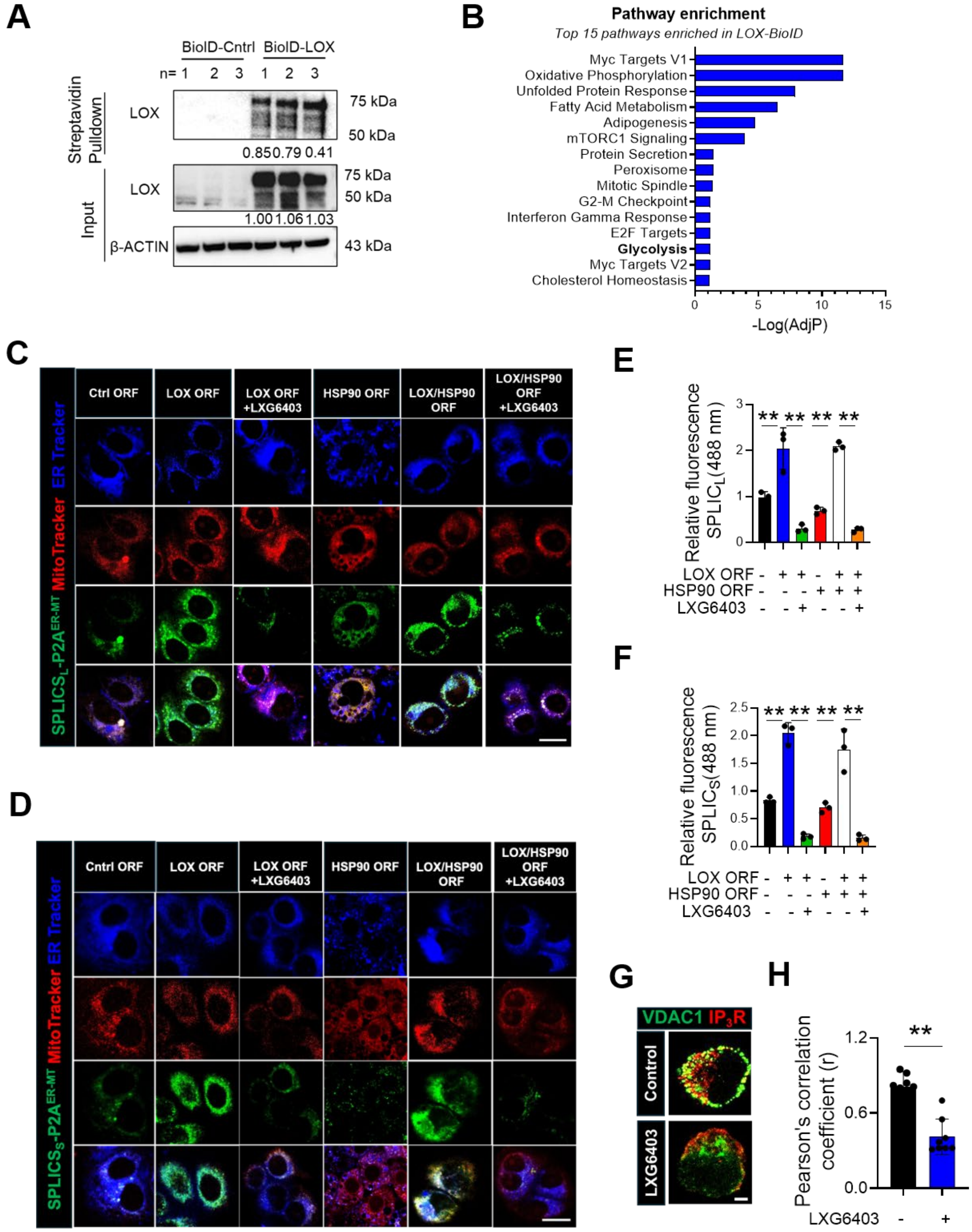
A
BioID-Cntrl BioID-LOX
n= 1 2 3 1 2 3
Streptavidin Pulldown
LOX
75 kDa
50 kDa
0.85 0.79 0.41
Input
LOX
75 kDa
50 kDa
1.00 1.06 1.03
β-ACTIN
43 kDa
B
Pathway enrichment
Top 15 pathways enriched in LOX-BioID
Myc Targets V1
Oxidative Phosphorylation
Unfolded Protein Response
Fatty Acid Metabolism
Adipogenesis
mTORC1 Signaling
Protein Secretion
Peroxisome
Mitotic Spindle
G2-M Checkpoint
Interferon Gamma Response
E2F Targets
Glycolysis
Myc Targets V2
Cholesterol Homeostasis
-Log(AdjP)
C
Ctrl ORF
LOX ORF
LOX ORF +LXG6403
HSP90 ORF
LOX/HSP90 ORF
LOX/HSP90 ORF +LXG6403
SPLICSL-P2A ER-MT MitoTracker ER Tracker
D
Cntrl ORF
LOX ORF
LOX ORF +LXG6403
HSP90 ORF
LOX/HSP90 ORF
LOX/HSP90 ORF +LXG6403
SPLICSS-P2A ER-MT MitoTracker ER Tracker
E
Relative fluorescence SPLICL(488 nm)
LOX ORF - + + - + +
HSP90 ORF - - - + + +
LXG6403 - - + - - +
F
Relative fluorescence SPLICS(488 nm)
LOX ORF - + + - + +
HSP90 ORF - - - + + +
LXG6403 - - + - - +
G
VDAC1 IP3R
Control
LXG6403
H
Pearson's correlation coefficient (r)
LXG6403 - +

Figure S4. Pathway enrichment analysis of the LOX interactome and effects of LOX-HSP90 axis on MERCs. Related to Figure 5. (A) Streptavidin pulldown followed by WB of LOX in MDA-MB-468 cells overexpressing BioID-Cntrl vs. BioID-LOX vector (n=3 biological replicates). (B) Pathway enrichment analysis of LOX interactors identified by BioID coupled to mass spectrometry. (C, D). Representative images from SPLICS reporters measuring long-distance (C) and short-distance (D) MERCs in low LOX/HSP90-expressing cells overexpressing LOX with or without HSP90 ORF and treated with LXG6403. Scale bar: 25 µm. (E, F) Quantification of SPLICS signals corresponding to long-range (E) and short-range (F) MERCs shown in (C) and (D), respectively (n=3 images). (G) IF staining of VDAC1 (mitochondria) and IP_3R (ER) in BT549 cells treated with LXG6403 (15 µM) for 12 hours. Scale bar: 10 µm. (H) Quantification of VDAC1-IP_3R co-localization from G (n=8 images). Data are presented as mean ± SD. *P*-values for the bar plots graph were calculated with the unpaired, two-tailed Student's t test (E, F, H). **, *P*-value < 0.01.

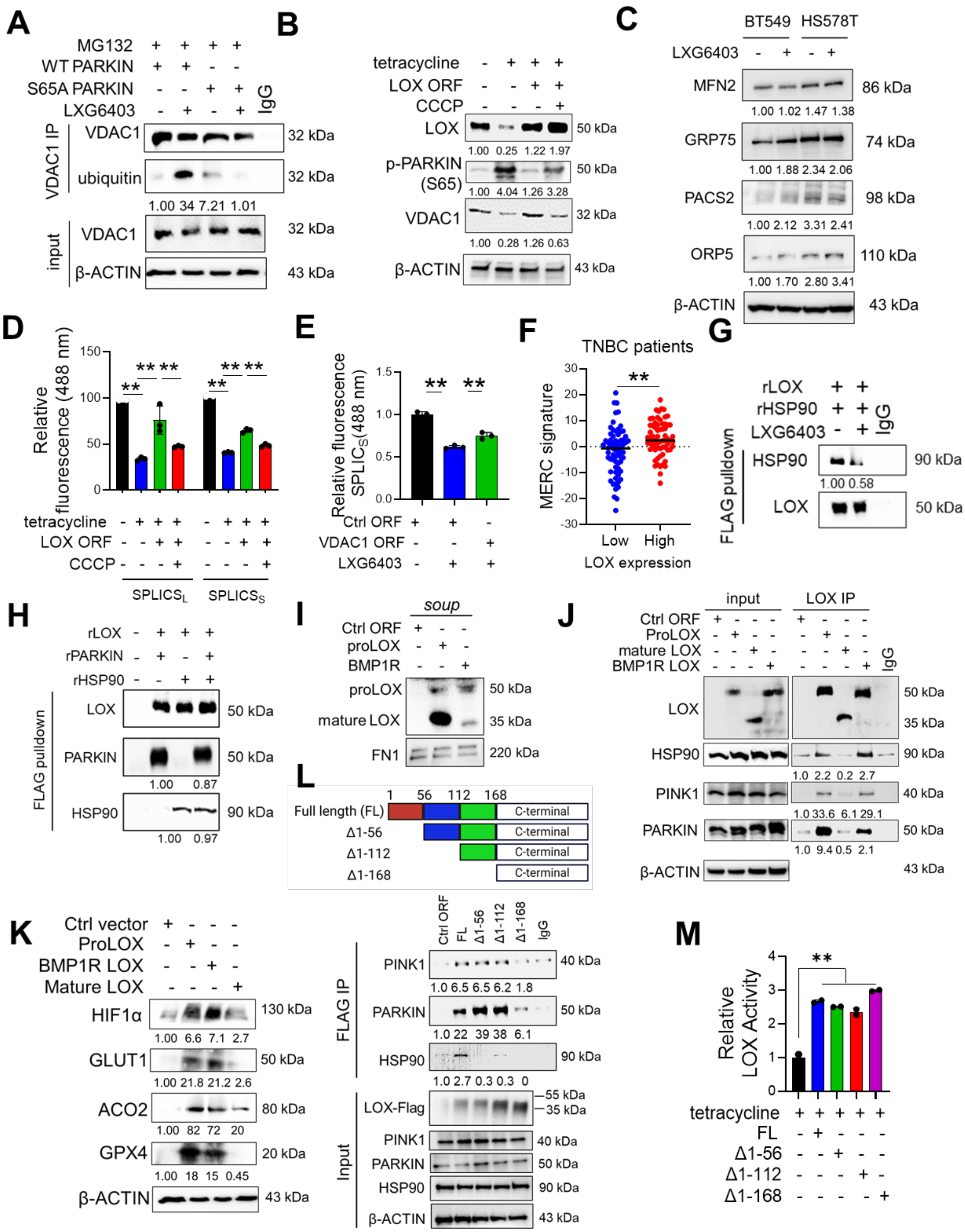

Figure S5. LOX inhibition-mediated PARKIN activation promotes VDAC1 ubiquitination and disrupts MERCs in an HSP90-independent manner and proLOX interacts with PINK1/PARKIN and HSP90. Related to Figure 5. (A) Ubiquitination of VDAC1 in cells expressing WT or S65A PARKIN following LOX inhibition using LXG6403 (30 μM) for 6 hours. MG132 (10 μM) was utilized to inhibit proteasomal degradation. (B) WB of LOX, P-PARKIN (S65), and VDAC1 in tetracycline inducible shLOX-expressing cells with or without LOX ORF. Mitophagy inducer, CCCP (2 μM) is used to re-activate mitophagy in the presence of LOX ORF as a control. (C) WB of MERC-associated proteins (MFN2, GRP75, PACS2, and ORP5) in vehicle- and LXG6403-treated TNBC cells. (D) Quantification of MERCs using $SPLICS_L$ (long MERCs) and $SPLICS_S$ (short MERCs) vectors in cells upon tetracycline-inducible LOX knockdown with or without LOX reconstitution in the presence or absence of CCCP (2 μM) (n=3 technical replicates). (E) Quantification of MERC formation in Ctrl- or VDAC1 ORF-transfected cells treated with vehicle or LXG6403 (n=3 technical replicates). (F) Levels of MERC signatures in low vs high LOX-expressing TNBC patients (n=200 patients). (G) Recombinant protein pull-down assay showing direct interaction between LOX and HSP90. (H) Recombinant protein pull-down assay with proLOX incubated with HSP90 and PARKIN separately or together. (I) WB of proLOX, and mature LOX in the soup of MDA-MB-231 cells overexpressing proLOX or BMP1R LOX. Fibronectin is used as a loading control. (J) co-IP showing binding of proLOX, mature LOX or BMP1R LOX to HSP90, PINK1, and PARKIN. (K) WB of HIF-1α, GLUT1, ACO2, and GPX4 in HEK293T cells expressing proLOX, mature LOX or BMP1R LOX. (L) co-IP of N-terminal proLOX deletion mutants and LOX-interacting partners. (M) LOX activity assay from cells in (L) (n=2 technical replicates). Data are presented as mean ± SD. *P*-values for the bar graphs or box plots were calculated with the unpaired, two-tailed Student's t test (D, E, F and M). **, *P*-value < 0.01.

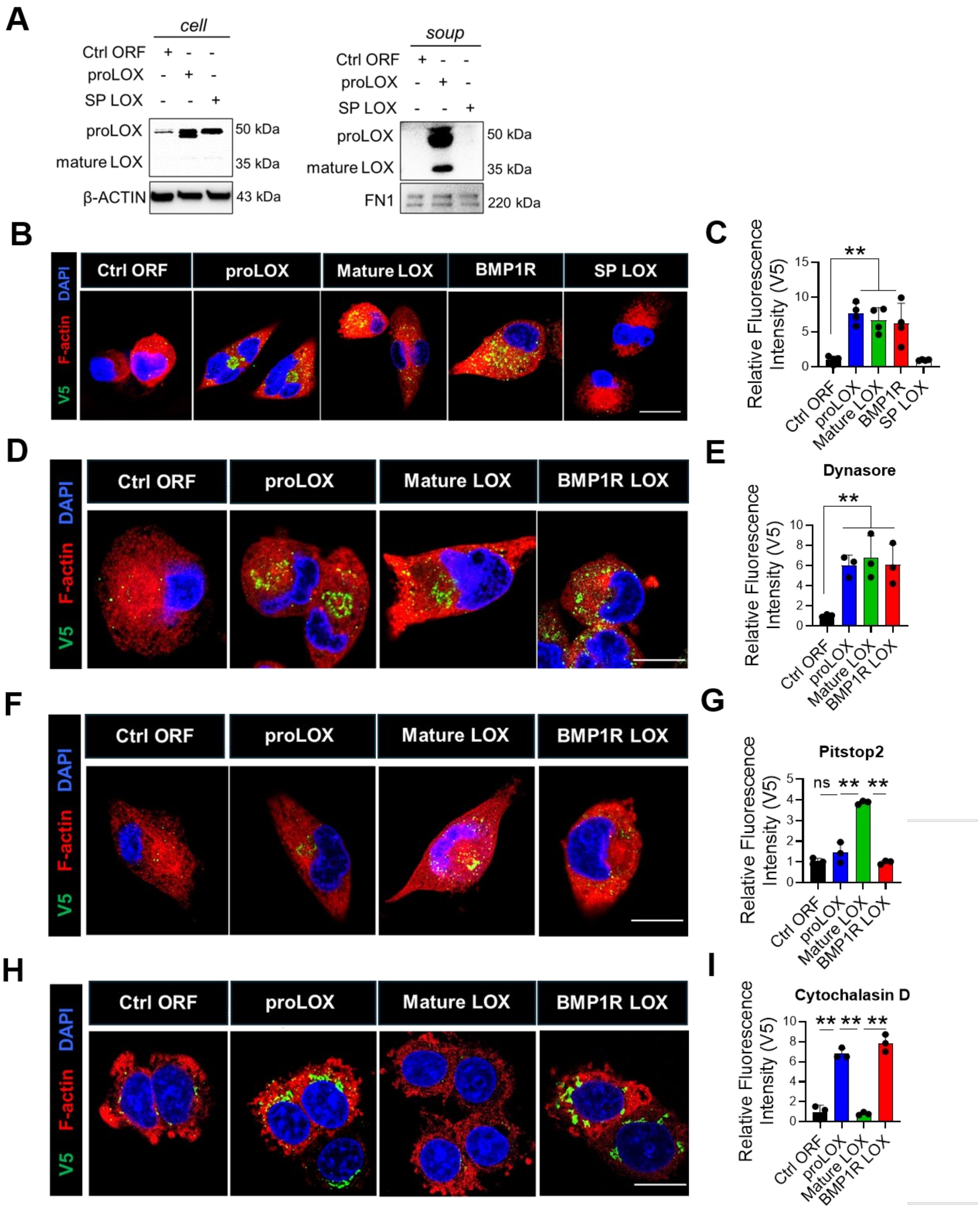
A
cell
Ctrl ORF + - -
proLOX - + -
SP LOX - - +
proLOX
mature LOX
β-ACTIN
50 kDa
35 kDa
43 kDa
soup
Ctrl ORF + - -
proLOX - + -
SP LOX - - +
proLOX
mature LOX
FN1
50 kDa
35 kDa
220 kDa
B
Ctrl ORF
proLOX
Mature LOX
BMP1R
SP LOX
DAPI
F-actin
V5
C
Relative Fluorescence Intensity (V5)
**
Ctrl ORF
proLOX
Mature LOX
BMP1R
SP LOX
D
Ctrl ORF
proLOX
Mature LOX
BMP1R LOX
E
Dynasore
**
Relative Fluorescence Intensity (V5)
F
G
Pitstop2
ns
**
H
I
Cytochalasin D
**

Figure S6. Identification of internalization mechanisms of different LOX forms and validation of BMP1R LOX and SP LOX constructs. Related to Figure 5. (A) WB of LOX inside the cells (left) or in the supernatant (right) of MDA-MB-231 cells expressing proLOX or SP LOX. (B, C) Internalization of proLOX, mature LOX, BMP1R LOX, and signal peptide defective SP LOX (B) and their quantification (C) (n=4 images). Scale bar: 10 μm. (D) Internalization of proLOX, mature LOX, and BMP1-resistant proLOX in the presence of the dynamin inhibitor Dynasore. Scale bar: 10 μm. (E) Quantification of (D) (n=3 images). (F) Internalization of proLOX, mature LOX, and BMP1-resistant proLOX in the presence of the clathrin/AP-2 inhibitor Pitstop2. Scale bar: 10 μm. (G) Quantification of (F) (n=3 images). (H) Internalization of proLOX, mature LOX, and BMP1R LOX in the presence of the actin polymerization inhibitor cytochalasin D. Scale bar: 10 μm. (I) Quantification of (H) (n=3 images). Data are presented as mean ± SD. *P*-values for bar plots were calculated using an unpaired, two-tailed Student's t test (C, E, G, I). **, *P*-value < 0.01, n.s.: not significant.

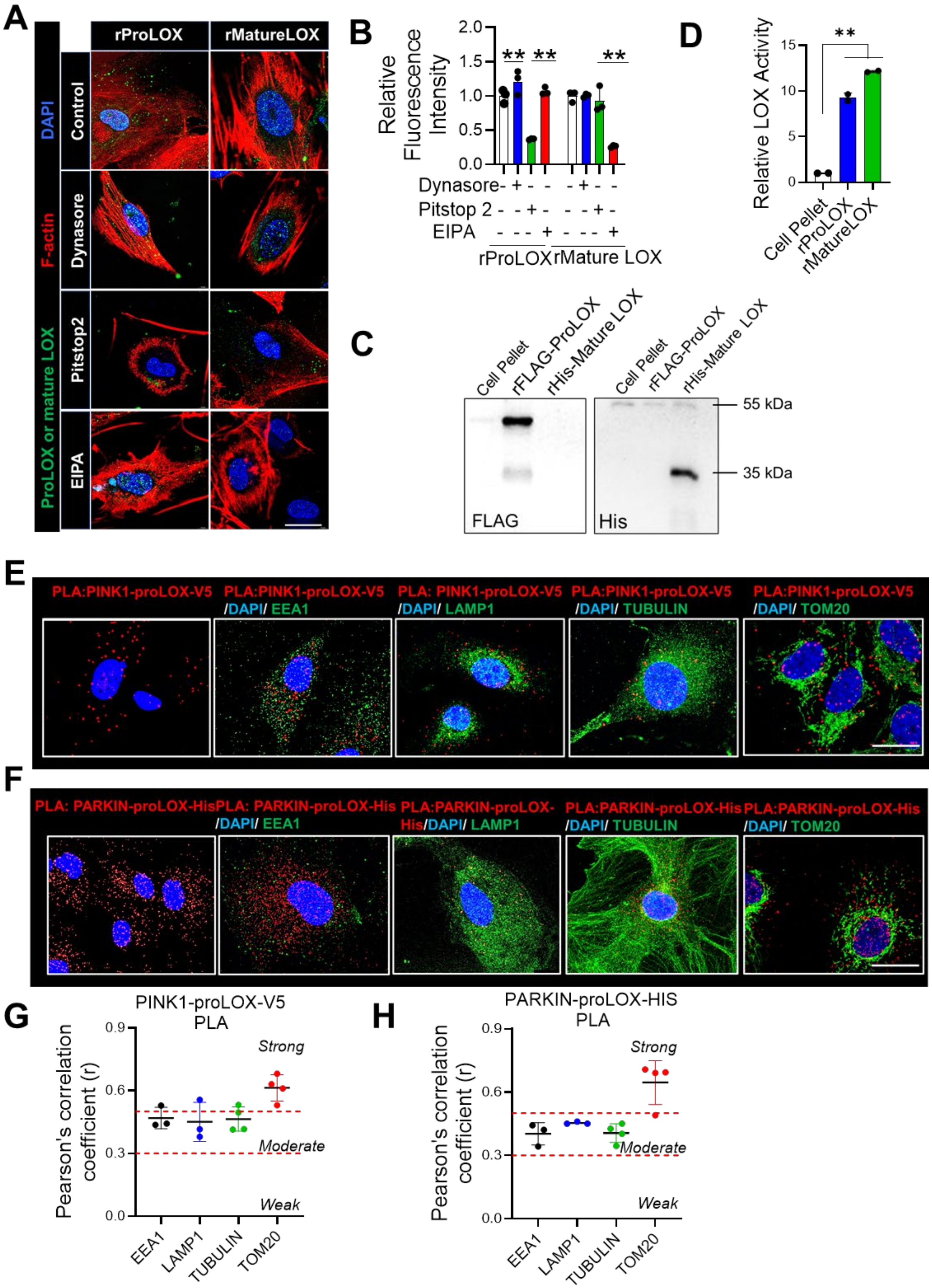
A
rProLOX
rMatureLOX
DAPI
F-actin
ProLOX or mature LOX
Control
Dynasore
Pitstop2
EIPA
B
Relative Fluorescence Intensity
Dynasore
Pitstop 2
EIPA
rProLOX
rMature LOX
C
Cell Pellet
rFLAG-ProLOX
rHis-Mature LOX
55 kDa
35 kDa
FLAG
His
D
Relative LOX Activity
Cell Pellet
rProLOX
rMatureLOX
E
PLA:PINK1-proLOX-V5
PLA:PINK1-proLOX-V5 /DAPI/ EEA1
PLA: PINK1-proLOX-V5 /DAPI/ LAMP1
PLA:PINK1-proLOX-V5 /DAPI/ TUBULIN
PLA:PINK1-proLOX-V5 /DAPI/ TOM20
F
PLA: PARKIN-proLOX-His
PLA: PARKIN-proLOX-His /DAPI/ EEA1
PLA:PARKIN-proLOX-His/DAPI/ LAMP1
PLA:PARKIN-proLOX-His /DAPI/ TUBULIN
PLA:PARKIN-proLOX-His /DAPI/ TOM20
G
PINK1-proLOX-V5 PLA
Pearson's correlation coefficient (r)
Strong
Moderate
Weak
EEA1
LAMP1
TUBULIN
TOM20
H
PARKIN-proLOX-HIS PLA
Pearson's correlation coefficient (r)
Strong
Moderate
Weak
EEA1
LAMP1
TUBULIN
TOM20

Figure S7. Recombinant LOX proteins are internalized and functionally active in recipient cells. Related to Figure 5. (A) Internalization of recombinant proLOX and mature LOX by Hs578T cells upon treatment with different endocytosis inhibitors. Scale bar: 10 µm. (B) Quantification of (A) (n=3 images). (C) WB of recombinant proLOX and mature LOX confirming the identity and integrity of the purified proteins used in internalization assays. (D) LOX enzymatic activity in recipient cell pellets following treatment with recombinant proLOX or mature LOX (n=2 technical replicates). (E, F) Representative images of PLA followed by immunofluorescence staining showing colocalization of proLOX-PINK1 (E) or PARKIN (F) PLA signals with the endosomal marker EEA1, lysosomal marker LAMP1, cytoskeleton marker TUBULIN, and mitochondria marker TOM20 in TNBC cells incubated with conditioned media containing His-V5-tagged proLOX. Scale bar: 10 µm. (G, H) Quantification of colocalization between proLOX with (V5)-PINK1 (G) or PARKIN (H) PLA signals and EEA1, LAMP1, TUBULIN or TOM20 by Pearson correlation analysis (n=3-4 images). Data are presented as mean ± SD. *P*-values for the bar plots graph were calculated with the unpaired, two-tailed Student's t test (B, D). Statistical significance was determined using Pearson correlation analysis (G, H). **, *P*-value < 0.01.

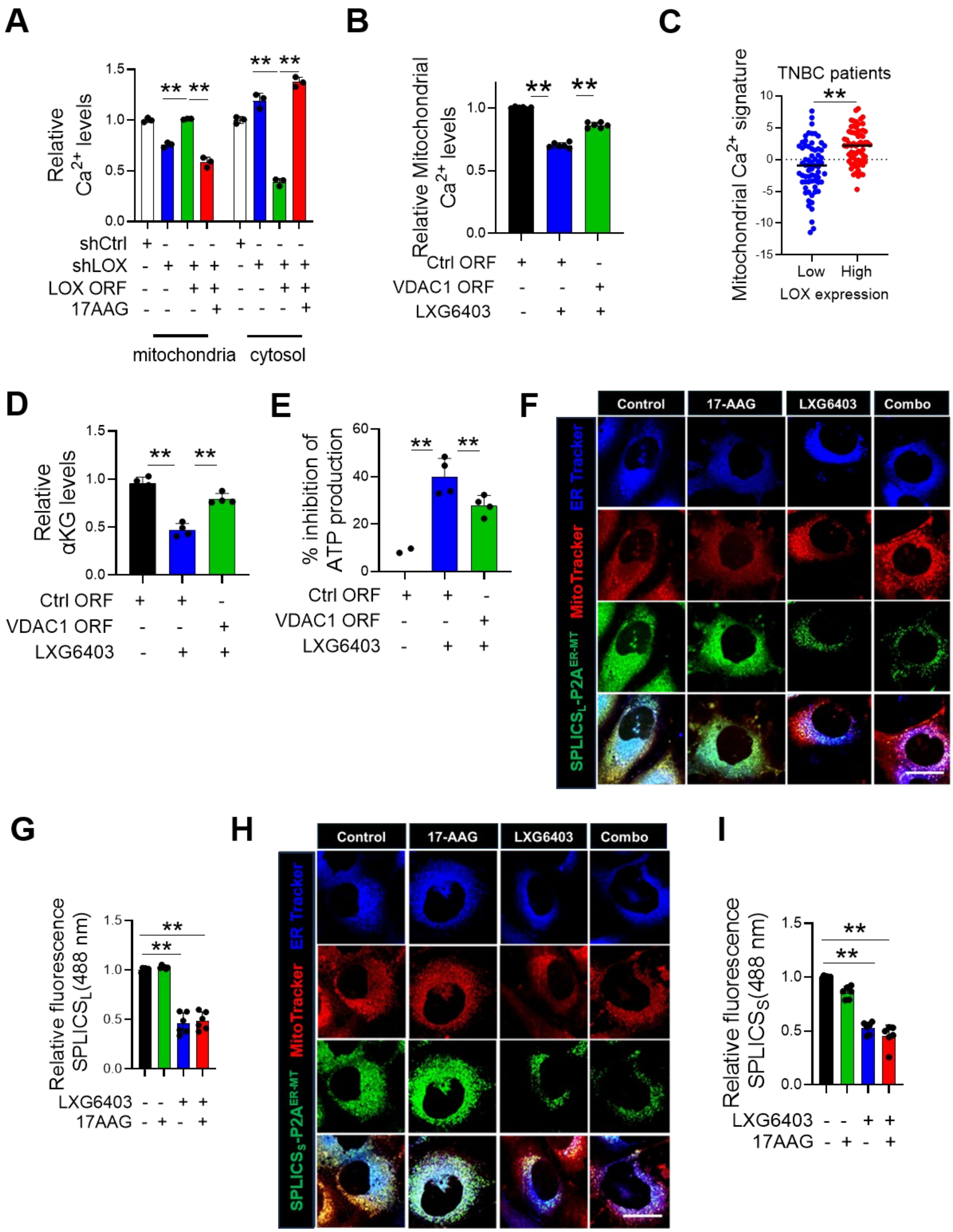

A
Relative Ca2+ levels
shCtrl
shLOX
LOX ORF
17AAG
mitochondria
cytosol
B
Relative Mitochondrial Ca2+ levels
Ctrl ORF
VDAC1 ORF
LXG6403
C
TNBC patients
Mitochondrial Ca2+ signature
Low
High
LOX expression
D
Relative αKG levels
Ctrl ORF
VDAC1 ORF
LXG6403
E
% inhibition of ATP production
Ctrl ORF
VDAC1 ORF
LXG6403
F
Control
17-AAG
LXG6403
Combo
ER Tracker
MitoTracker
SPLICSL-P2AER-MT
G
Relative fluorescence SPLICSL(488 nm)
LXG6403
17AAG
H
Control
17-AAG
LXG6403
Combo
ER Tracker
MitoTracker
SPLICSS-P2AER-MT
I
Relative fluorescence SPLICSS(488 nm)
LXG6403
17AAG

Figure S8. LOX-HSP90 signaling sustains mitochondrial Ca^{2+}, α-ketoglutarate, and ATP production, and LOX is associated with mitochondrial Ca^{2+} signatures in TNBC patients, and LOX inhibition-mediated disruption of MERCs is not further enhanced by HSP90 inhibition. Related to Figure 5. (A) Relative levels of mitochondrial and cytosolic Ca^{2+} in MDA-MB-231 cells upon inducible LOX knockdown with tetracycline with or without LOX reconstitution in the presence or absence of HSP90 inhibitor 17AAG (n=3 technical replicates). (B) Quantification of mitochondrial Ca^{2+} levels in Ctrl or VDAC1 ORF-transfected cells treated with LXG6403 (n=2 biological and n=6 technical replicates). (C) Levels of mitochondrial Ca^{2+} signature in low vs high LOX-expressing TNBC patients (n=200 patients). (D) Relative α-ketoglutarate (α-KG) levels in Ctrl or VDAC1 ORF-transfected cells treated with LXG6403 (n=4 technical replicates). (E) ATP levels in Ctrl or VDAC1 ORF-transfected cells treated with LXG6403 (n=4 technical replicates). (F, H) Representative images from SPLICS reporters measuring long-range (F) and short-range (H) MERCs in high LOX/HSP90-expressing cells treated with the HSP90 inhibitor (17AAG) and LXG6403. Scale bar: 25 µm. (G, I) Quantification of SPLICS signals corresponding to long-range (G) and short-range (I) MERCs shown in (F) and (H), respectively (from n=6 images). Data are presented as mean ± SD. *P*-values were calculated using an unpaired, two-tailed Student's t test (A-E, G, I). **, *P*-value < 0.01.

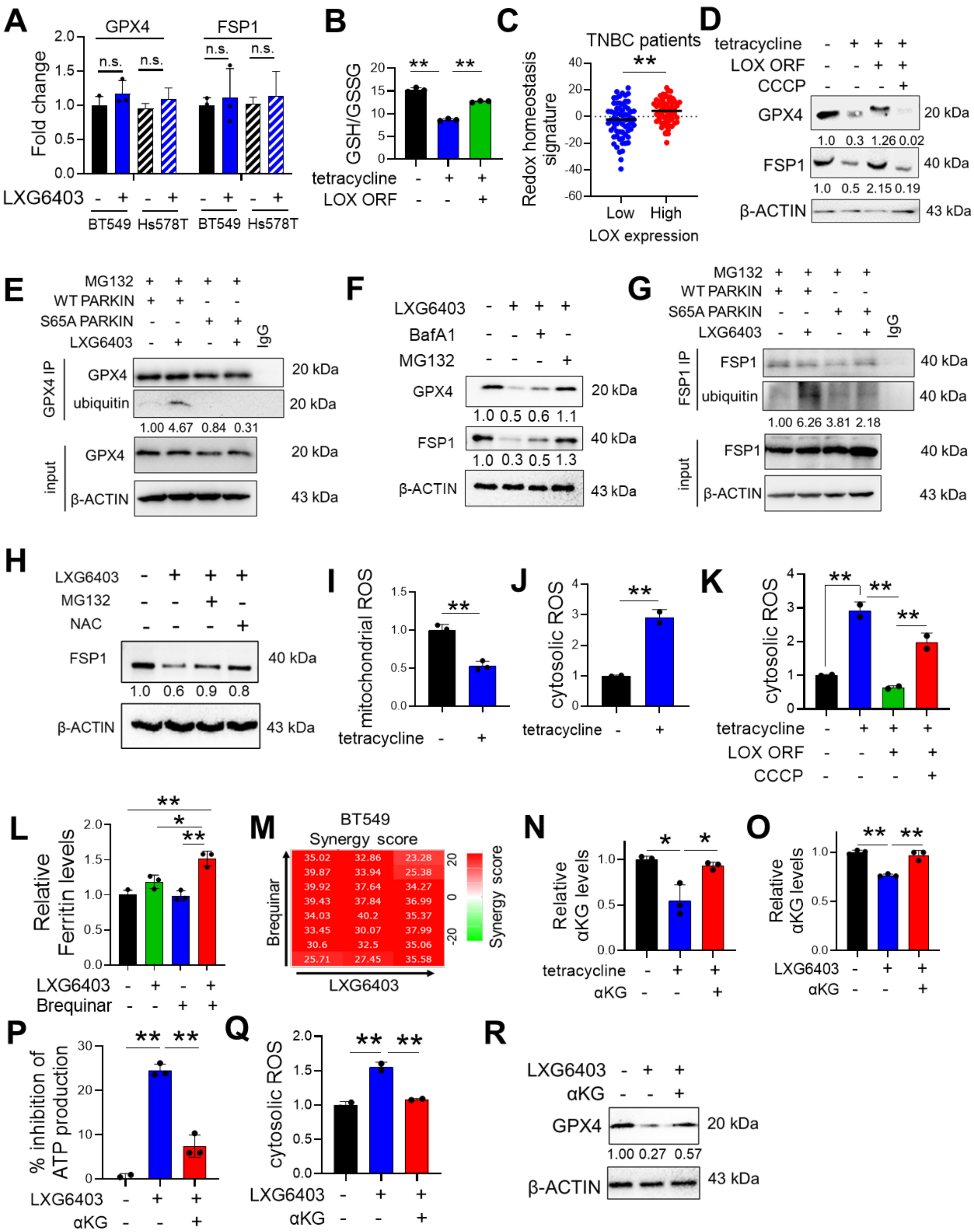
A
GPX4
FSP1
n.s.
Fold change
2.0
1.5
1.0
0.5
0.0
LXG6403 - + - + - + - +
BT549 Hs578T BT549 Hs578T
B
GSH/GSSG
**
tetracycline - + +
LOX ORF - - +
C
TNBC patients
Redox homeostasis signature
Low High
LOX expression
D
tetracycline - + + +
LOX ORF - - + +
CCCP - - - +
GPX4
20 kDa
1.0 0.3 1.26 0.02
FSP1
40 kDa
1.0 0.5 2.15 0.19
β-ACTIN
43 kDa
E
MG132 + + + +
WT PARKIN + + - -
S65A PARKIN - - + +
LXG6403 - + - +
IgG
GPX4 IP
GPX4
ubiquitin
1.00 4.67 0.84 0.31
input
β-ACTIN
F
LXG6403 - + + +
BafA1 - - + -
MG132 - - - +
GPX4
1.0 0.5 0.6 1.1
FSP1
1.0 0.3 0.5 1.3
β-ACTIN
G
MG132 + + + +
WT PARKIN + + - -
S65A PARKIN - - + +
LXG6403 - + - +
IgG
FSP1 IP
FSP1
ubiquitin
1.00 6.26 3.81 2.18
input
β-ACTIN
H
LXG6403 - + + +
MG132 - - + -
NAC - - - +
FSP1
1.0 0.6 0.9 0.8
β-ACTIN
I
mitochondrial ROS
tetracycline - +
J
cytosolic ROS
tetracycline - +
K
cytosolic ROS
tetracycline - + + +
LOX ORF - - + +
CCCP - - - +
L
Relative Ferritin levels
LXG6403 - + - +
Brequinar - - + +
M
BT549
Synergy score
35.02 32.86 23.28
39.87 33.94 25.38
39.92 37.64 34.27
39.43 37.84 36.99
34.03 40.2 35.37
33.45 30.07 37.99
30.6 32.5 35.06
25.71 27.45 35.58
Brequinar
LXG6403
Synergy score
N
Relative αKG levels
tetracycline - + +
αKG - - +
O
Relative αKG levels
LXG6403 - + +
αKG - - +
P
% inhibition of ATP production
LXG6403 - + +
αKG - - +
Q
cytosolic ROS
LXG6403 - + +
αKG - - +
R
LXG6403 - + +
αKG - - +
GPX4
20 kDa
1.00 0.27 0.57
β-ACTIN
43 kDa

Figure S9. LOX inhibition induces mitophagy-dependent, GPX4 and FSP1 degradation, ROS accumulation, and disruption of redox homeostasis, leading to DHODH dependency. Related to Figure 6. (A) Relative GPX4 and FSP1 mRNA expression levels following LXG6403 treatment, showing no significant changes at the transcriptional level (n=3 technical replicates). (B) Intracellular GSH/GSSG ratio following tetracycline-inducible LOX knockdown with or without LOX ORF (n=3 technical replicates). (C) Levels of redox homeostasis signature in low vs high LOX-expressing TNBC patients (n=200 patients). (D) WB of GPX4, and FSP1 upon tetracycline-inducible shLOX expression with or without LOX reconstitution. Mitophagy inducer, CCCP is used to re-activate mitophagy in the presence of LOX ORF as a control. (E) WB of GPX4 ubiquitination in TNBC cells transfected with WT or S65A PARKIN and treated with LXG6403. (F) WB of GPX4 and FSP1 upon treatment with LXG6403 in the presence or absence of the proteasome inhibitor MG132 or the lysosomal inhibitor bafilomycin A1 (BafA1). (G) WB of FSP1 ubiquitination in TNBC cells transfected with WT or S65A PARKIN and treated with vehicle or LXG6403. (H) WB of FSP1 upon 24 hours of LXG6403 treatment (15 μM) with or without MG132 (10 μM) and the ROS scavenger NAC (1 mM) added at the last 6 hours of treatment. (I, J) Mitochondrial (I) and cytosolic (J) ROS levels upon tetracycline-inducible LOX knockdown for 24 hours (n= 2-3 technical replicates). (K) Cytosolic ROS levels in cells from (D) (n=2 technical replicates). (L) Relative ferritin levels in MDA-MB-231 cells treated with the combination of LXG6403 (15 μM) and the DHODH inhibitor brequinar (50 μM) for 2 hours (n=3 technical replicates). (M) Synergy score of BT549 cells treated with multiple doses of brequinar in combination with LXG6403 for 3 days (n=3 technical replicates). (N, O) Relative αKG levels in 231.shLOX cells upon tetracycline inducible LOX knockdown (N) or LXG6403 (O) -/+ αKG. (P) ATP levels upon LXG6403 (15 μM) treatment -/+ αKG (n=3 technical replicates). (Q) Cytosolic ROS levels upon LXG6403 (15 μM) treatment -/+ αKG (n=3 technical replicates). (R) WB of GPX4 upon LXG6403 (15 μM) treatment -/+ αKG. Data are presented as mean ± SD. *P*-values for the bar plots graph were calculated with

the unpaired, two-tailed Student's t test (A-C, I-Q) *, *P*-value < 0.05; **, *P*-value < 0.01; n.s., not significant.

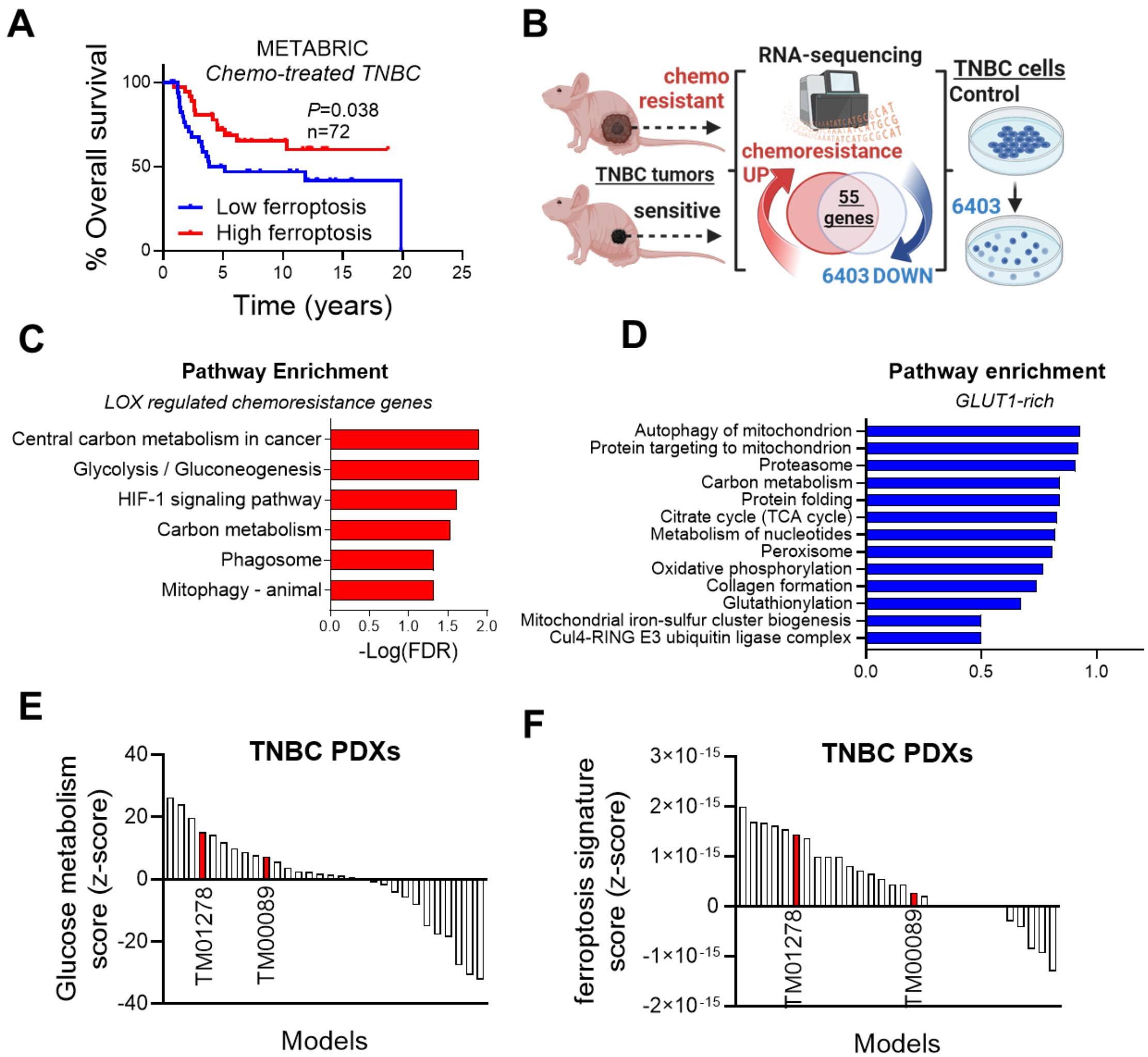

Figure S10. Clinical relevance of a ferroptosis signature in chemo-treated TNBCs, gene set enrichment analysis of LOX-regulated chemoresistance gene signature and selection of TNBC PDXs based on glucose metabolism and ferroptosis sensitivity. Related to Figure 7. A) Kaplan-Meier survival analyses in chemo-treated TNBC patients from METABRIC stratified by ferroptosis signature scores (n=72 patients). (B) Schematic summary of the pipeline used to determine LOX-regulated chemoresistant genes. (C) Pathway enrichment analysis among LOX-

regulated chemoresistant genes. (D) Clustering analysis in genes highly expressed in GLUT1-rich regions of TNBC tumors. (E, F) Glucose metabolism (E) and ferroptosis signature (F) scores in TNBC PDXs. Significance for the Kaplan-Meier survival curves was calculated with Log-rank test (A).

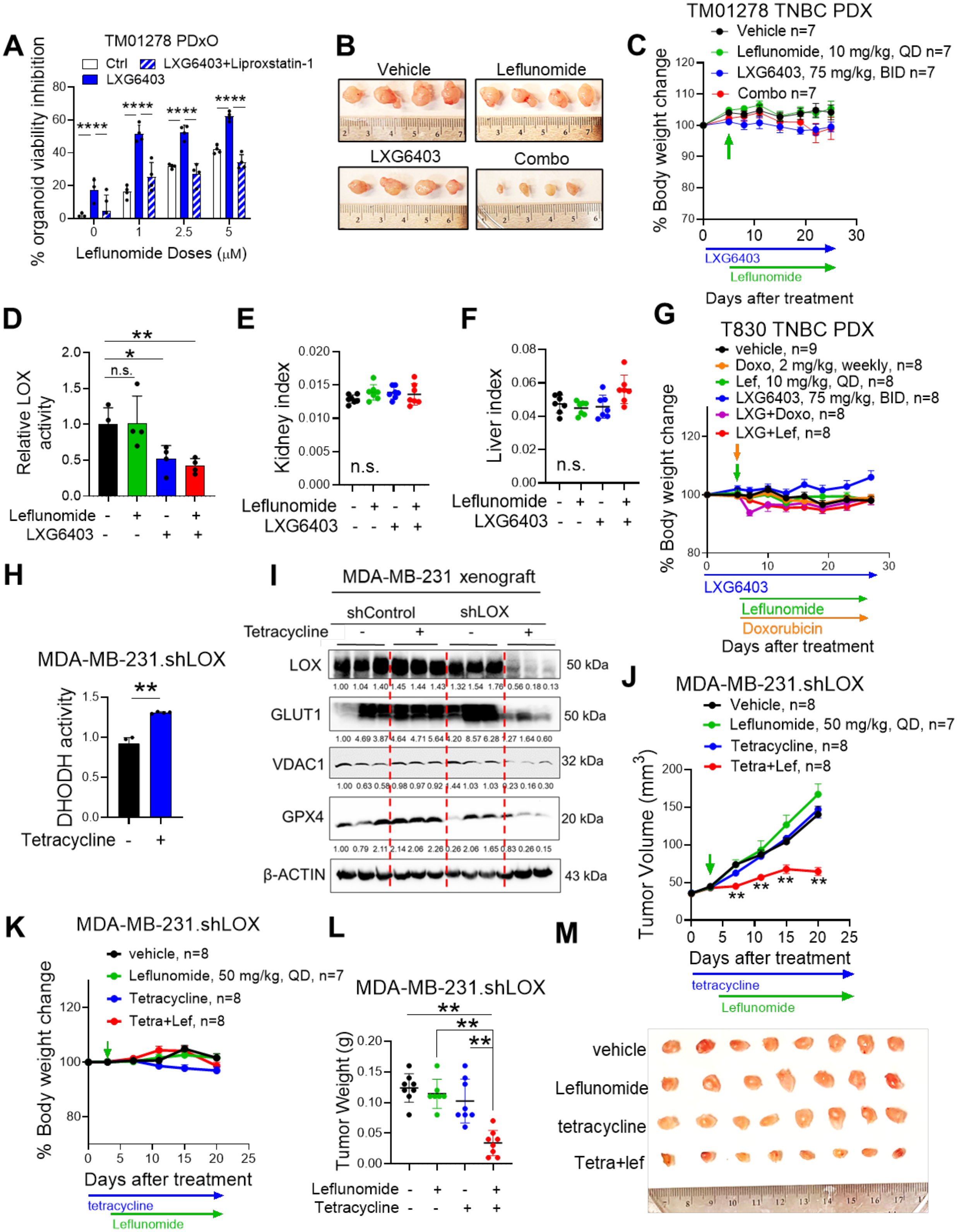
A
TM01278 PDxO
Ctrl
LXG6403+Liproxstatin-1
LXG6403
% organoid viability inhibition
Leflunomide Doses (μM)
B
Vehicle
Leflunomide
LXG6403
Combo
C
TM01278 TNBC PDX
Vehicle n=7
Leflunomide, 10 mg/kg, QD n=7
LXG6403, 75 mg/kg, BID n=7
Combo n=7
% Body weight change
LXG6403
Leflunomide
Days after treatment
D
Relative LOX activity
n.s.
Leflunomide
LXG6403
E
Kidney index
n.s.
Leflunomide
LXG6403
F
Liver index
n.s.
Leflunomide
LXG6403
G
T830 TNBC PDX
vehicle, n=9
Doxo, 2 mg/kg, weekly, n=8
Lef, 10 mg/kg, QD, n=8
LXG6403, 75 mg/kg, BID, n=8
LXG+Doxo, n=8
LXG+Lef, n=8
% Body weight change
LXG6403
Leflunomide
Doxorubicin
Days after treatment
H
MDA-MB-231.shLOX
DHODH activity
Tetracycline
I
MDA-MB-231 xenograft
shControl
shLOX
Tetracycline
LOX
50 kDa
GLUT1
50 kDa
VDAC1
32 kDa
GPX4
20 kDa
β-ACTIN
43 kDa
J
MDA-MB-231.shLOX
Vehicle, n=8
Leflunomide, 50 mg/kg, QD, n=7
Tetracycline, n=8
Tetra+Lef, n=8
Tumor Volume (mm3)
Days after treatment
tetracycline
Leflunomide
K
MDA-MB-231.shLOX
vehicle, n=8
Leflunomide, 50 mg/kg, QD, n=7
Tetracycline, n=8
Tetra+Lef, n=8
% Body weight change
Days after treatment
tetracycline
Leflunomide
L
MDA-MB-231.shLOX
Tumor Weight (g)
Leflunomide
Tetracycline
M
vehicle
Leflunomide
tetracycline
Tetra+lef

Figure S11. Combination of genomic and pharmacological LOX inhibition with DHODH inhibitor leflunomide suppresses tumor growth in chemoresistant TNBC models without detectable toxicity. Related to Figure 7. (A) Quantification of organoid viability in TM01278 PDxOs treated with leflunomide (1, 2.5, and 5 µM) and LXG6403 (10 µM) for 5 days (n=4 technical replicates). (B, C) Representative tumor images (B) and % body weight change (C) in TM01278 PDXs treated with vehicle, leflunomide (10 mg/kg, daily, p.o.), LXG6403 (75 mg/kg, bidaily, p.o.) or the combination for 25 days (n=7 mice per group). (D) LOX activity in TNBC PDXs treated with LXG6403, leflunomide or their combination (n=4 mice per group). (E, F) Kidney (E) and liver indices (F) (organ weight/body weight) in TNBC PDXs treated with LXG6403, leflunomide or their combination (n=7 mice per group). (G) % Body weight change of T830 TNBC PDX tumors derived from a patient with recurrent disease following multi-agent chemotherapy, treated with vehicle, LXG6403 (75 mg/kg, bidaily, p.o.), leflunomide (10 mg/kg, daily, p.o.), doxorubicin (2 mg/kg, weekly, i.v.) or the combination with LXG6403 for 28 days (n=8-9 mice per group). (H) DHODH activity in shLOX tumors upon tetracycline-inducible LOX knockdown. (I) WB of LOX, GLUT1, VDAC1, and GPX4 upon tetracycline inducible LOX knockdown in shCtrl- vs. shLOX-expressing MDA-MB-231 tumors (n=3 tumors). (J, K) Tumor volume (J) and body weight change (%) (K) of MDA-MB-231.shLOX xenografts upon tetracycline-inducible LOX knockdown in combination with leflunomide (50 mg/kg, daily, p.o.) (n=7-8 mice per group). (L, M) End point tumor weight (L) and tumor images (M) from mice in (J) (n=7-8 mice per group). Data are presented as mean values ± SD for (A, D, E, F, H and L) and as mean values ± SEM for (C, G, J and K). Significance for the tumor volume and body weight graphs were calculated using the Wilcoxon rank-sum test (C, G, J, K). *P*-values for the dot plots and bar graphs were calculated with the unpaired, two-tailed Student's t test (A, D, E, F, H, L) *, *P*-value < 0.05; **, *P*-value < 0.01; n.s., not significant.

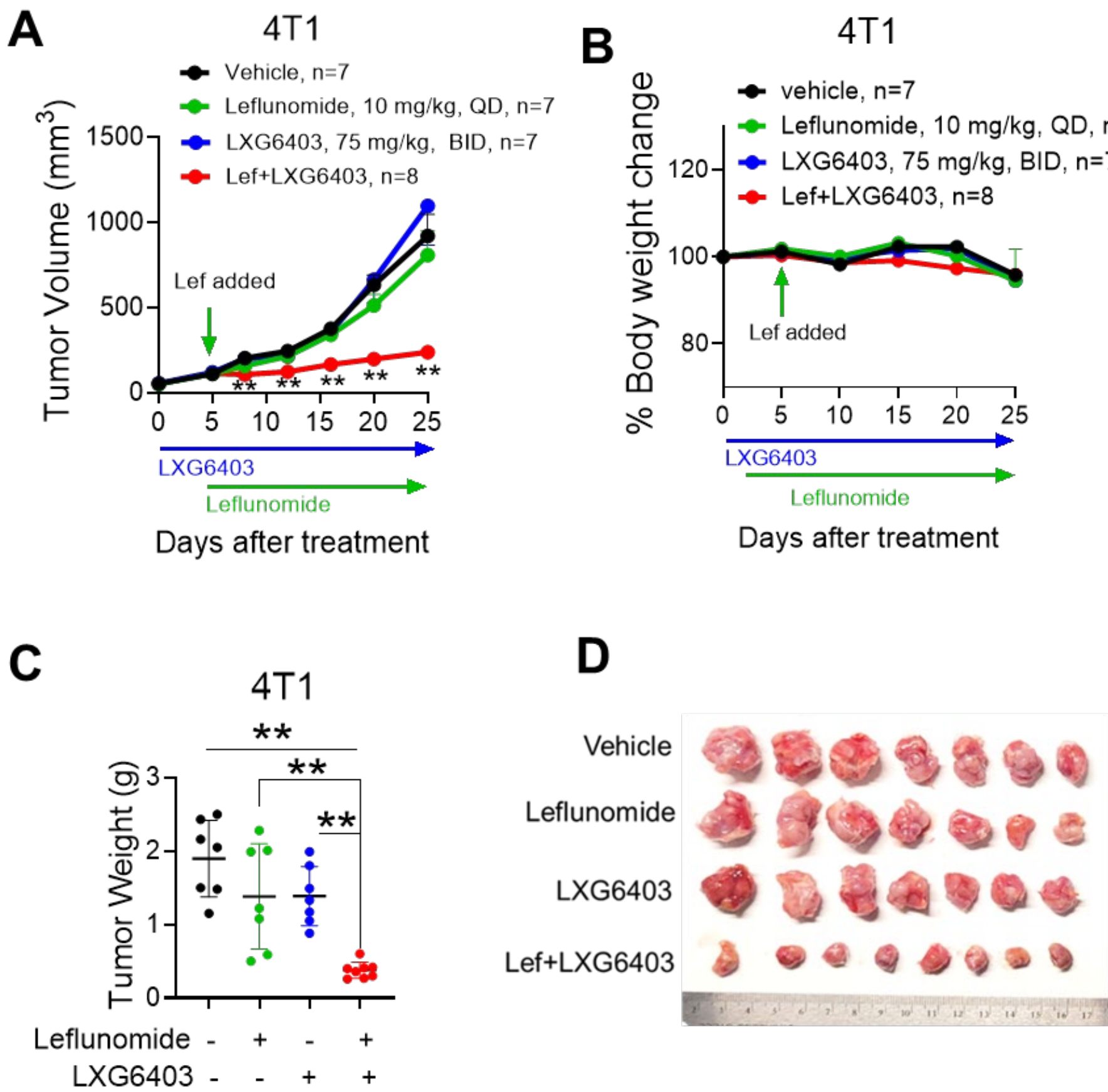

Figure S12. LOX inhibition in combination with DHODH suppresses tumor growth in an immunocompetent TNBC model. Related to Figure 7. (A-C) Tumor volume (A), body weight change (B), and endpoint tumor weights (C) of 4T1 TNBC tumors injected in BALB/c mice treated with vehicle, LXG6403 (75 mg/kg, bidaily, p.o), leflunomide (10 mg/kg, daily, p.o) or the combination for 25 days (n=7-8 mice per group). (D) Images of tumors collected at the endpoint. Data are presented as mean values ± SD for (C) and as mean values ± SEM for (A and B). Significance for the tumor volume and body weight graphs were calculated using the Wilcoxon rank-sum test (A and B). *P*-values for the dot plots graph were calculated with the unpaired, two-tailed Student's t test (C). **, *P*-value < 0.01.

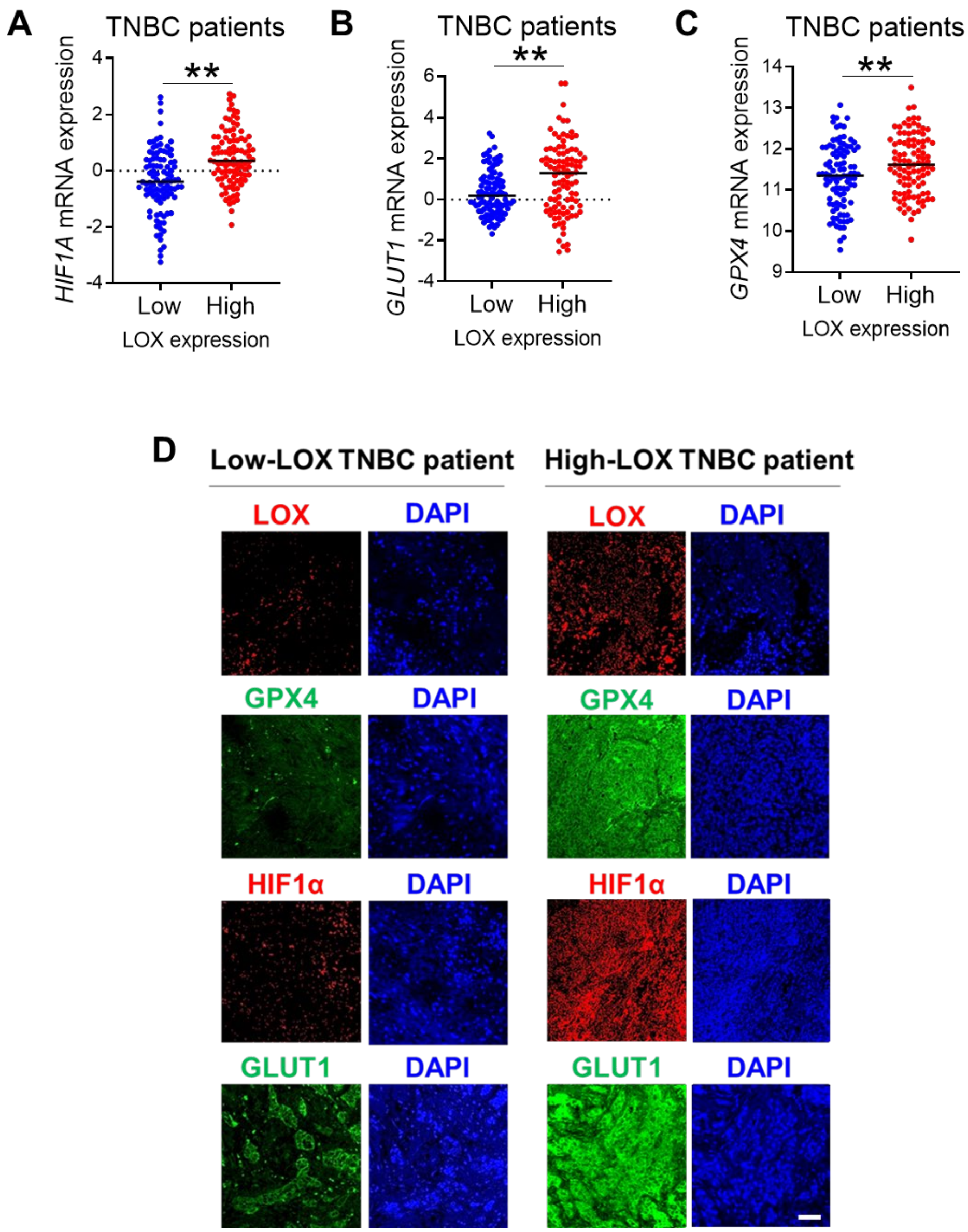

294

Figure S13. Levels of HIF-1α, GLUT1, and GPX4 correlate with LOX in TNBC patient datasets and representative immunofluorescence staining in TNBC patient tumors. Related to Figure 7. (A-C) mRNA expression of *HIF-1A* (A), *GLUT1* (B), *GPX4* (C) in low vs. high LOX-expressing TNBC patients from METABRIC (A, B, n=200 patients) or GSE76124 (n=198 patients). (D) Representative images of immunofluorescence staining of LOX, GPX4, HIF-1α, and GLUT1 in TNBC patient tumors. Scale bar: 200 μm. *P*-values for the dot plots graph were calculated with the unpaired, two-tailed Student's t test (A-C). **, *P*-value < 0.01.

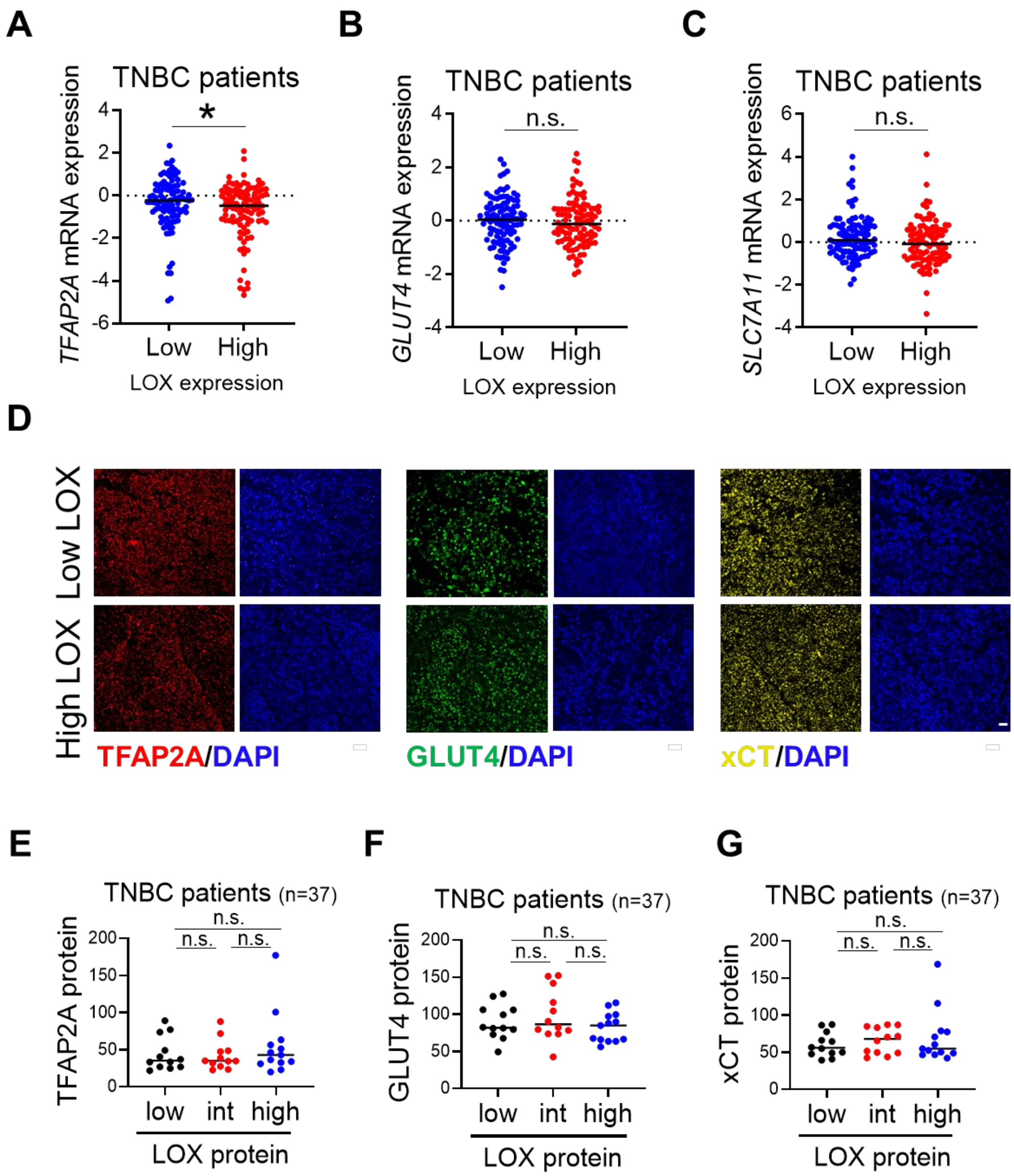

Figure S14. mRNA and protein levels of GLUT4, xCT, and TFAP2A do not correlate with LOX in TNBC patients. Related to Figure 7. (A-C) Levels of *TFAP2A*, *GLUT4*, and *SLC7A11* mRNA levels in low vs/ high-LOX expressing TNBC tumors from METABRIC (n=200 patients). (D) Representative immunofluorescence staining of GLUT4, xCT, and TFAP2A in TNBC. Scale bar: 200 µm. (E-G) The mean intensities of GLUT4, xCT, and TFAP2A proteins in TNBC patient tumors (n=37 patients) were stratified into three groups based on LOX expression (low,

intermediate, and high). *P*-values for the dot plots graph were calculated with the unpaired, two-tailed Student's t test (A-C, E-G) *, *P*-value < 0.05; n.s., not significant.

SUPPLEMENTAL TABLES

Table S1. Related to Figures 1-7. List of antibodies and their dilutions used in WB, IP, and IF.

Antibody	WB dilution	IP dilution	IF dilution
**GLUT1**	1:1000		1:100
**HIF-1α**	1:1000		1:100
**His-Tag**	1:7500		
**HA**	1:7500		
**V5**		1:100	1:50
**PARKIN**	1:1000		1:50
**PARKIN**	1:1000	1:50	
**p-PARKIN (S65)**	1:1000		
**PINK1**	1:1000		1:50
**β-ACTIN**	1:10000		
**COXIV**	1:1000		
**ACO2**	1:1000		
**LC3**	1:1000		
**p62**	1:1000		
**HSP90**	1:1000		1:50
**TOM20**			1:50
**OPA1**			1:50
**VDAC1**	1:1000		1:50
**IP3R**			1:50
**MFF**			1:50
**DRP1**			1:50
**HSPA5**	1:1000		
**KEAP1**	1:1000		
**FSP1**	1:1000		1:100
**GPX4**	1:1000		1:250
**Ubiquitin**	1:2000		
**DHODH**	1:1000		
**MFN1**	1:1000		
**pRB**	1:1000		
**LOX**	1:1000	1:250	1:250
**VHL**	1:1000		
**TFAP2A**			1:100
**xCT**			1:100
**GLUT4**			1:250
**EEA1**			1:100
**LAMP1**			1:100
**TUBA4A**			1:100

**PACS2**	1:1000		
**MFN2**	1:1000		
**GRP75**	1:1000		
**ORP5**	1:1000		
**HRP-linked anti-mouse IgG**	1:1000		
**HRP-linked anti-rabbit IgG**	1:1000		
**Alexa Fluor 488 anti-mouse**			1:1000
**Alexa Fluor 488 anti-rabbit**			1:1000
**Alexa Fluor 647 anti-mouse**			1:1000
**Alexa Fluor 647 anti-rabbit**			1:1000
**Alexa Fluor 488 anti-goat**			1:1000
**Anti Fluor 488 anti-chicken**			1:1000

Table S2. Related to Figures 1 and 2. Primer sequences used in qRT-PCR.

**Primer**	**Sequence**
**LOX forward primer**	GGATACGGCACTGGCTACTT
**LOX reverse primer**	GACGCCTGGATGTAGTAGGG
**GLUT1 forward primer**	AGAAGGTGATCGAGGAGTTCTA
**GLUT1 reverse primer**	CACAGAGAAGGAGCCAATCAT
**LDHA forward primer**	GGATCTCCAACATGGCAGCCTT
**LDHA reverse primer**	AGACGGCTTTCTCCCTCTTGCT
**PGK1 forward primer**	CCGCTTTCATGTGGAGGAAGAAG
**PGK1 reverse primer**	CTCTGTGAGCAGTGCCAAAAGC
**ENO1 forward primer**	CTGACCAAGACTCCAGCCAGAA
**ENO1 reverse primer**	GAGTCTGCGTTGCCTCAGTTGT
**SLC16A3 forward primer**	CCACAAGTTCTCCAGTGCCATTG
**SLC16A3 reverse primer**	CGCCAGGATGAACACGTACATG
**PKM forward primer**	CGGAAGGACACGGCATCAAGAT
**PKM reverse primer**	GAGCCAGGAAAACCTTCTCTGC
**HK2 forward primer**	GAGCCACCACTCACCCTACT
**HK2 reverse primer**	CCAGGCATTCGGCAATGTG
**FN1 forward primer**	CTGGCCGAAAATACATTGTAAA

**FN1 reverse primer**	CCACAGTCGGGTCAGGAG
**ACTB forward primer**	CCAACCGCGAGAAGATGA
**ACTB reverse primer**	CCAGAGGCGTACAGGGATAG
**HPRT forward primer**	TGACCTTGATTTATTTTGCATACC
**HPRT reverse primer**	CGAGCAAGACGTTCAGTCCT
